# Diketopiperazine Derivatives from the Marine-Derived Actinomycete *Streptomyces* sp. FXJ7.328

**DOI:** 10.3390/md11041035

**Published:** 2013-03-28

**Authors:** Pei Wang, Lijun Xi, Peipei Liu, Yi Wang, Wei Wang, Ying Huang, Weiming Zhu

**Affiliations:** 1 Key Laboratory of Marine Drugs, Ministry of Education of China, School of Medicine and Pharmacy, Ocean University of China, Qingdao 266003, China; E-Mails: wangpei850212@163.com (P.W.); liupeipei@ouc.edu.cn (P.L.); wangyi0213@hotmail.com (Y.W.); wwwakin@ouc.edu.cn (W.W.); 2 State Key Laboratory of Microbial Resources, Institute of Microbiology, Chinese Academy of Sciences, Beijing 100101, China; E-Mail: xilijun1002@163.com

**Keywords:** *Streptomyces*, diketopiperazine derivatives, antivirus activity, H1N1

## Abstract

Five new diketopiperazine derivatives, (3*Z*,6*E*)-1-*N*-methyl-3-benzylidene-6-(2*S*-methyl-3-hydroxypropylidene)piperazine-2,5-dione (**1**), (3*Z*,6*E*)-1-*N*-methyl-3-benzylidene-6-(2*R*-methyl-3-hydroxypropylidene)piperazine-2,5-dione (**2**), (3*Z*,6*Z*)-3-(4-hydroxybenzylidene)-6-isobutylidenepiperazine-2,5-dione (**3**), (3*Z*,6*Z*)-3-((1*H*-imidazol-5-yl)-methylene)-6-isobutylidenepiperazine-2,5-dione (**4**), and (3*Z*,6*S*)-3-benzylidene-6-(2*S*-but-2-yl)piperazine-2,5-dione (**5**), were isolated from the marine-derived actinomycete *Streptomyces* sp. FXJ7.328. The structures of **1**–**5** were determined by spectroscopic analysis, CD exciton chirality, the modified Mosher’s, Marfey’s and the C_3_ Marfey’s methods. Compound **3** showed modest antivirus activity against influenza A (H1N1) virus with an IC_50_ value of 41.5 ± 4.5 μM. In addition, compound **6** and **7** displayed potent anti-H1N1 activity with IC_50_ value of 28.9 ± 2.2 and 6.8 ± 1.5 μM, respectively. Due to the lack of corresponding data in the literature, the ^13^C NMR data of (3*Z*,6*S*)-3-benzylidene-6-isobutylpiperazine-2,5-dione (**6**) were also reported here for the first time.

## 1. Introduction

**Figure 1:**
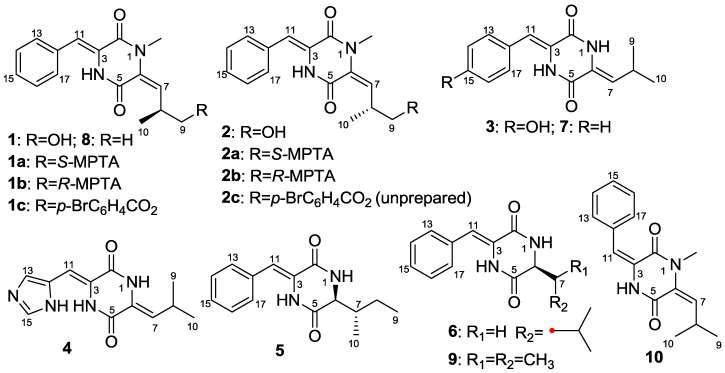
Chemical structures of compounds **1**–**10** from *Streptomyces* sp. FXJ7.328.

Marine actinomycetes are a rich source of bioactive compounds with new structures [1], e.g., the cytotoxic thiocoraline from *Micromonospora* sp. L-13-ACM2-092 [2], and the anti-inflammatory cyclomarins A–C from *Streptomyces* sp. CNB-382 [3]. As one of the most important classes of bioactive compounds, alkaloids have received much attention. As part of our ongoing research on bioactive alkaloids with new structures from marine-derived actinomycetes [4,5,6,7], *Streptomyces* sp. FXJ7.328 was isolated from marine sediment, and was found to produce alkaloids by TLC visualizing with Dragendorff’s reagent in a saline culture. The ethyl acetate extract of fermentation broth exhibited antivirus activity against H1N1 influenza virus at 100 μg/mL and displayed a series of peaks with UV absorptions at 220 and 340 nm similar to those of diketopiperazine derivatives such as albonoursin and (3*Z*,6*S*)-3-benzylidene-6-isobutylpiperazine-2,5-dione by HPLC-UV analysis [8]. Chemical investigation of the extract resulted in the isolation and identification of five new diketopiperazine derivatives (Figure 1), namely (3*Z*,6*E*)-1-*N*-methyl-3-benzylidene-6-(2*S*-methyl-3-hydroxypropylidene)-piperazine-2,5-dione (**1**), (3*Z*,6*E*)-1-*N*-methyl-3-benzylidene-6-(2*R*-methyl-3-hydroxypropylidene)-piperazine-2,5-dione (**2**), (3*Z*,6*Z*)-3-(4-hydroxybenzylidene)-6-isobutylidenepiperazine-2,5-dione (**3**), (3*Z*,6*Z*)-3-((1*H*-imidazol-5-yl)methylene)-6-isobutylidenepiperazine-2,5-dione (**4**), and (3*Z*,6*S*)-3-benzylidene-6-(2*S*-but-2-yl)piperazine-2,5-dione (**5**). In addition, five known analogues were also isolated and their structures were identified as (3*Z*,6*S*)-3-benzylidene-6-isobutylpiperazine-2,5-dione (**6**) [8], albonoursin (**7**) [8,9,10], (3*Z*,6*E*)-1-*N*-methyl-3-benzylidene-6-isobutylidenepiperazine-2,5-dione (**8**) [9,11], (3*Z*,6*S*)-3-benzylidene-6-isopropylpiperazine-2,5-dione (**9**) [12], and (3*E*,6*E*)-1-*N*-methyl-3-benzylidene-6-isobutylidenepiperazine-2,5-dione (**10**) [13], respectively, by comparing their NMR data and specific rotation (Supplementary Information) with those reported in the literatures. Compounds **3**, **6** and **7** displayed activity against influenza A (H1N1) virus with the IC_50_ values of 41.5 ± 4.5, 28.9 ± 2.2 and 6.8 ± 1.5 μM, respectively. 

## 2. Results and Discussion

### 2.1. Structure Elucidation

The ethyl acetate extract of the fermentation broth of *Streptomyces* sp. FXJ7.328 was subjected to extensive chromatographic separations over silica gel, Sephadex LH-20 and by HPLC to yield the new compounds **1**–**5** and the known analogues **6**–**10**. 

**Figure 2:**
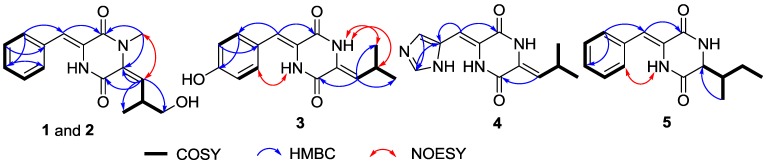
Selected 2D NMR correlations for **1**–**5**.

**Figure 3:**
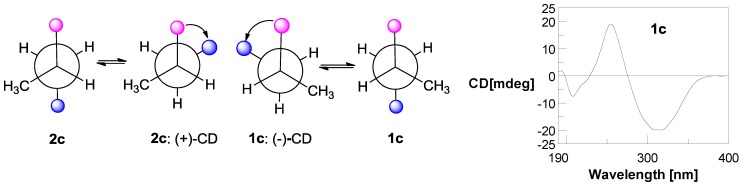
The stable conformers of **1c** and **2c** and the measured CD curve for *p*-bromobenzoate **1c**.

Compounds **1** and **2** were at first isolated as a racemic mixture with an excess of one enantiomer and thought to be a pure compound according to their identical NMR data and a small negative specific rotation ([α]_D_ −5.8). When (*R*)-Mosher’s acyl chloride was used to determine the absolute configuration, the Mosher’s ester was found to be a separable mixture according to ^1^H NMR spectrum and HPLC profile. A chiral HPLC column was used to separate the enantiomeric mixture of **1** and **2**, which yielded compounds **1** and **2** in an approximate ratio of 2:1 (Figure S37). The planar structures of **1** and **2** were established based on MS and NMR data of the racemic mixture. HRESIMS gave an ion peak at *m*/*z* 287.1387 [M + H]^+^ (calcd. for C_16_H_19_N_2_O_3_ 287.1390), corresponding to the molecular formula C_1__6_H_1__8_N_2_O_3_. Analysis of the 1D NMR data (Table 1) revealed one methyl (δ_H/C_ 1.01/17.9), one *N*-methyl (δ_H/C_ 3.17/31.3), one sp^3^ oxygenated methylene (δ_H/C_ 3.36/66.7), seven sp^2^ methines, one sp^3^ methine (δ_H/C_ 3.59/34.9), and two signals (δ_C_ 158.5, 158.9) interpreted as conjugated amide carbonyls. ^1^H NMR signals at δ_H_ 7.52 (2H, d, *J* = 7.7), 7.40 (2H, t, *J* = 7.7) and 7.31 (1H, t, *J* = 7.2) revealed the presence of a monosubstituted benzene nucleus that was further identified as a dehydro-phenylalanine (deh-Phe) unit from the key HMBC correlations of H-13/17 (δ_H_ 7.52) to C-11 (δ_C_ 116.1) and C-15 (δ_C_ 128.7), and of H-11 (δ_H_ 6.75) to C-13 (δ_C_ 129.9) and C-2 (δ_C_ 158.5). ^1^H-^1^H COSY correlations of H-7/H-8/H-9 and H-8/H-10 combined with the key HMBC correlations from H-7 (δ_H_ 5.56) to C-5 (δ_C_ 158.9) and C-6 (δ_C_ 130.4), and from N-CH_3_ (δ_H_ 3.17) to C-6 (δ_C_ 130.4) suggested another amino acid unit, *N*-methyl dehydro-5-hydroxyleucine. The key HMBC correlation of N-CH_3_ to C-2 finally established the conjugate diketopiperazine (DKP) structure of **1** and **2** (Figure 2). The NOE correlation from N-CH_3_ to H-7 indicated the *E*-configuration of the Δ^6^ double bond. The *Z*-configuration of Δ^3,11^ double bond could be deduced from the relative downfield shift of H-11 because of the deshielding effect of the 2-carbonyl group, as e.g., δ_H__-11_ 6.85, 6.40 and 6.74 for (3*Z*,6*E*)-1-*N*-methyl-3-benzylidene-6-isobutylidenepiperazine-2,5-dione [11], (3*E*,6*E*)-1-*N*-methyl-3-benzylidene-6-isobutylidenepiperazine-2,5-dione [13], and (3*Z*,6*Z*)-3-benzylidene-6-isobutylidenepiperazine-2,5-dione [10], respectively. The *S*-configuration of **1** was determined by analysis of exciton chirality CD of its *p*-bromobenzoate (**1c**) [14]. The stable conformers of the *p*-bromobenzoates of **1c** and its enantiomer **2c** (unprepared) were obtained by HyperChem Release 7.5 software [4] (Figure 3). The transition dipole orientations of two chromophores, the conjugated DKP core and the *p*-bromobenzoate in **1c** and **2c**, were oriented in counterclockwise and clockwise manners, which should result in negative and positive Cotton effects at long wavelength, respectively. The measured negative CD effect of **1c** at λ_ext_ 313 nm (Δε −5.2) indicates the *S*-configuration of **1c** (Figure 3). This deduction was further validated by modified Mosher’s method for primary alcohols [15,16]. When **1** reacted with *R*- and *S*-MTPA chloride, the *S*- (**1a**) and *R*-MTPA esters (**1b**) were obtained, respectively. The chemical shift difference between two methylene protons of C-9 in *S*-MTPA ester **1a** is larger than that in *R*-MTPA ester **1b** (Δδ 0.08 *vs*. 0.01), indicating *S*-configuration of C-8 in **1**. Thus, the structure of **1** was clearly elucidated as (3*Z*,6*E*)-1-*N*-methyl-3-benzylidene-6-(2*S*-methyl-3-hydoxypropylidene)-piperazine-2,5-dione. Compound **2** showed the opposite specific rotation and the opposite chemical shift difference between the two methylene protons of C-9 in *S*- and *R*-MTPA esters (**2a** and **2b**), indicating *R*-configuration of **2**. So compound **2** was identified as (3*Z*,6*E*)-1-*N*-methyl-3-benzylidene-6-(2*R*-methyl-3-hydroxypropylidene)piperazine-2,5-dione.

**Table 1:** ^1^H and ^13^C NMR Data for **1**–**5** (600 and 150 MHz, DMSO-*d*_6_, δ values).

Position	1 and 2		3		4		5	
	δ_C_, type	δ_H_, mult. (*J* in Hz)	δ_C_, type	δ_H_, mult. (*J* in Hz)	δ_C_, type	δ_H_, mult. (*J* in Hz)	δ_C_, type	δ_H_, mult. (*J* in Hz)
1	31.3, NCH_3_	3.17, s		10.24, s				8.47, s
2	158.5, qC		158.0, qC		157.8, qC		161.0, qC	
3	126.5, qC		125.3, qC		125.2, qC		127.3, qC	
4				9.82, s				9.93, s
5	158.9, qC		157.4, qC		156.8, qC		166.8, qC	
6	130.4, qC		125.2, qC		125.9, qC		60.2, CH	3.86, t, (3.3)
7	129.7, CH	5.56, d, (9.9)	125.1, CH	5.66, d, (10.4)	125.5, CH	5.68, d, (9.9)	40.9, CH	1.80, m
8	34.9, CH	3.59, m	23.9, CH	2.93, m	24.4, CH	2.95, m	24.8, CH_2_	1.46, m; 1.18, m
9	66.7, CH_2_	3.36	22.2, CH_3_	0.96, d, (6.5)	22.8, CH_3_	0.97, d, (6.6)	15.3, CH_3_	0.91, d, (7.1)
10	17.9, CH_3_	1.01, d, (7.1)	22.2, CH_3_	0.96, d, (6.5)	22.8, CH_3_	0.97, d, (6.6)	12.1, CH_3_	0.86, t, (7.7)
11	116.1, CH	6.75, s	115.0, CH	6.66, s	105.1, CH	6.60, s	114.6, CH	6.66, s
12	133.9, qC		123.9, CH		137.0, qC		133.9, qC	
13/17	129.9, CH	7.52, d, (7.7)	130.9, CH	7.36, d, (8.5)	119.8, CH	7.52, s	129.3, CH	7.45, d, (7.7)
14/16	129.2, CH	7.40, t, (7.7)	115.6, CH	6.79, d, (8.5)			129.7, CH	7.39, t, (7.7)
15	128.7, CH	7.31, t, (7.2)	157.5, qC		137.1, CH	7.94, s	128.5, CH	7.29, t, (7.7)

The molecular formula of compound **3** was determined to be C_1__5_H_16_N_2_O_3_ based on HRESIMS with a peak at *m*/*z* 273.1231 [M + H]^+^ (calcd. for C_15_H_17_N_2_O_3_ 273.1234). The similarity of the 1D NMR spectra to those of albonoursin (**7**) [8,9] indicated a conjugated DKP as well. The difference of ^1^H and ^13^C NMR data between **3** and **7** pointed to a *p*-hydroxy substituted phenyl system in **3** instead of the benzene ring in **7**, which explains the obvious upfield shifts of H-13/17 (δ_H_ 7.36), H-14/16 (δ_H_ 6.79) and C-14/16 (δ_C_ 115.6) by the electron-donor effect of the hydroxy group. ^1^H-^1^H COSY correlations between H-13/17 and H-14/16 and the key HMBC correlations from H-11 (δ_H_ 6.66) to C-13/17 (δ_C_ 130.9) and C-2 (δ_C_ 158.0), from H-13/17 to C-15 (δ_C_ 157.5), and from H-14/16 to C-12 (δ_C_ 123.9) further supported the existence of a dehydro-tyrosine (deh-Tyr) unit. The ^1^H-^1^H COSY correlations from H-7 (δ_H_ 5.66) to H-9/10 (δ_H_ 0.96) through H-8 (δ_H_ 2.93) along with the HMBC correlations from H-7 to C-5 (δ_C_ 157.4) supported the existence of a dehydro-leucine (deh-Leu) unit. The NOE correlation between H-4 (δ_H_ 9.82) and H-13/17 indicated the *Z*-configuration of the Δ^3,11^ double bond. The NOE correlations of H-1 (δ_H_ 10.24) to H-8 and H-9/10 combined with the relative downfield shift of H-7 revealed *Z*-configuration of the Δ^6 ^ double bond. Therefore, compound **3** was determined to be (3*Z*,6*Z*)-3-(4-hydroxybenzylidene)-6-isobutylidenepiperazine-2,5-dione. 

Compound **4** was found to have the molecular formula of C_12_H_14_N_4_O_2_ from the HRESIMS peak at *m*/*z* 247.1189 [M + H]^+^ (calcd. for C_12_H_15_N_4_O_2_ 247.1190). 1D NMR (Table 1) and 2D NMR (Figure 2) data disclosed the same *Z*-deh-Leu unit as in compound **3**. The remainder C_6_H_5_N_3_O displayed three sp^2^ methine signals at δ_H__/__C_ 6.60/105.1, 7.94/137.1 and 7.52/119.8, two sp^2^ quaternary carbon signals at δ_C_ 125.2 and 125.9, and one amide carbonyl signal at δ_C_ 157.8. The HMBC correlations of H-15 (δ_H_ 7.94) to C-13 (δ_C_ 119.8) and C-12 (δ_C_ 137.0), of H-13 (δ_H_ 7.52) to C-15 (δ_C_ 137.1), and of H-11 (δ_H_ 6.60) to C-2 (δ_C_ 157.8) and C-12 suggested a dehydro-histidine (deh-His) unit. The *Z*-configurations of both Δ^3,11^ and Δ^6^ double bonds were deduced from the relative downfield shifts of H-11 and H-7 consistent with those of (3*Z*,6*R*)-3-((1*H*-imidazol-5-yl)methylene)-6-isopropylpiperazine-2,5-dione (δ_H_ 6.51) [17] and compound **3**, respectively. Compound **4** was therefore elucidated as (3*Z*,6*Z*)-3-((1*H*-imidazol-5-yl)methylene)-6-isobutylidenepiperazine-2,5-dione. 

The molecular formula of C_15_H_18_N_2_O_2_ was assigned to **5** according to the HRESIMS peak at *m*/*z* 259.1439 [M + H]^+^ (calcd. for C_15_H_19_N_2_O_2_ 259.1441), indicating an isomer of **6**. The 1D NMR (Table 1) spectra were very similar to those of **6** [8] except for the leucine moiety signals, suggesting that a deh-Phe unit was also presented in the structure of **5**. The NOE correlation from H-4 to H-13/17 and the relative downfield shift of H-11 accounted for the *Z*-configuration of the Δ^3,11^ double bond [8]. The main differences of the ^1^H NMR spectra are a methyl triplet in **5** replacing the methyl doublet in **6**; further, a distinct split methylene signal in **5** substitutes the overlapped methylene proton signal in **6**. These observations combined with the separate downfield and upfield shifts of methine and methylene carbon signals revealed the existence of an isoleucine moiety in **5**, which was further confirmed by the ^1^H-^1^H COSY correlations of H-1/H-6/H-7/H-8/H-9 and H-7/H-10 (Figure 2) and the key HMBC correlation of H-10 to C-6. The absolute configuration of the isoleucine moiety was determined by Marfey’s method [18] combined with C_3_ Marfey’s method [19,20]. The 1-fluoro-2,4-dinitrophenyl-5-l-alanine amide (FDAA) derivatives of the acid hydrolysates of **5** and four authentic isoleucine samples (l-, l-*allo*-, d- and d-*allo*-) were prepared. HPLC analysis over ODS column (Figure S38) revealed that acid hydrolysates of **5** displayed the same retention time (*t*_R_ 23.06 min) as the authentic l-Ile (*t*_R_ 23.06 min) and l-*allo*-Ile (*t*_R_ 23.06 min) but were different from d-Ile (*t*_R_ 28.11 min) and d-*allo*-Ile (*t*_R_ 28.11 min). The FDAA derivatives of the acid hydrolysates of **5** and the authentic l-Ile and l-*allo*-Ile were further analyzed by C_3_ HPLC column (Figure S39). The retention time of the acid hydrolysates of **5** was the same as for the authentic l-Ile (*t*_R_ 38.50 min), but different from the authentic l-*allo*-Ile (*t*_R_ 37.49 min). Thus, the isoleucine moiety in **5** was unambiguously identified as l-Ile, and the structure of compound **5** was elucidated as (3*Z*,6*S*)-3-benzylidene-6-(2*S*-but-2-yl)piperazine-2,5-dione.

### 2.2. The Postulated Biosynthesis Pathway of Compounds **1**–**10**

**Figure 4:**
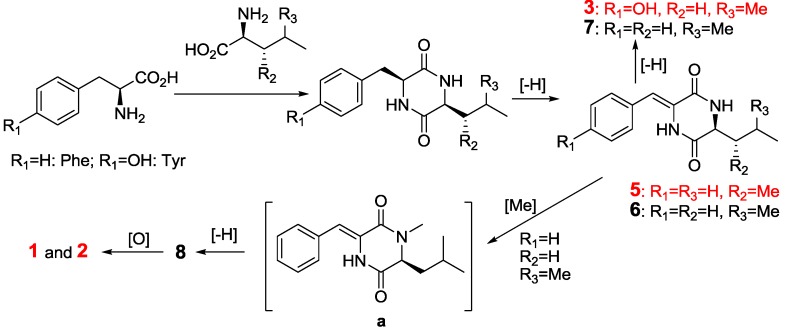
The postulated biosynthetic pathway of **1**–**3** and **5**–**8**.

Compounds **1**–**10** were postulated to be produced biogenetically from the amino acid pathway (Figure 4). Cyclic condensation between Phe and Leu formed *cyclo*(Phe-Leu) that underwent dehydrogenation in Phe moiety to form compounds **6** and **7**. The *N*-methylation of **6** produced an un-isolated intermediate (**a**) that further underwent another dehydrogenation in the Leu moiety to form compound **8**. The oxidation of **8** at the same homoallylic positions produced compounds **1** and **2**. By a similar biosynthetic pathway, compounds **3**–**5** and **9** were produced from the cyclic condensation and successive dehydration of Tyr with Leu, His with Leu, Phe with Ile, and Phe with Val, respectively. The dehydrogenation is favored to form *Z*-products because of the steric hindrance of the carbonyl oxygen, while the dehydrogenation of Leu moiety in the *N*-Me substituted compounds only produced the *E*-products due to the strong steric hindrance by *N*-Me. So, compound **10** could be an artifact produced from the photo-isomerization of compound **8** under light during the extraction and the subsequent isolation steps. 

### 2.3. The Bioactivities of Compounds **1**–**10** from *Streptomyces* sp. FXJ7.328

**Figure 5:**
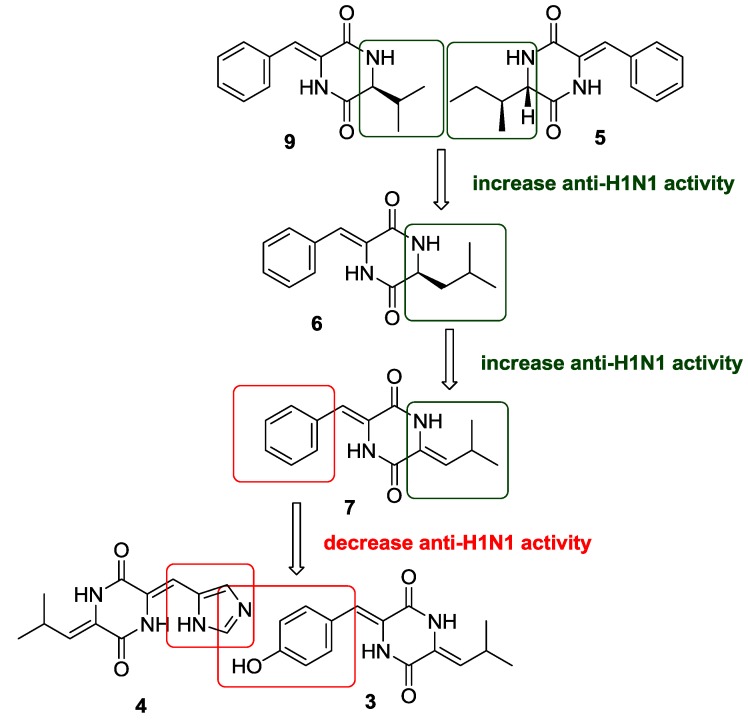
The structure-activity relationship (SAR) of compounds **3**–**7** and **9** for anti-H1N1 viral activity.

Compounds **1** and **3**–**10** were tested for antivirus effects on H1N1 by the CPE inhibition assay [21,22], separately. The IC_50_ values of **1**, **3**–**10** and ribavirin (positive control) were 75.5 ± 2.2, 41.5 ± 4.5, 62.6 ± 3.9, 106.5 ± 4.2, 28.9 ± 2.2, 6.8 ± 1.5, 94.5 ± 3.0, 113.8 ± 4.9, 156.6 ± 4.0, and 38.8 ± 1.5 μM, respectively. Except for compounds **3**, **6** and **7**, the other compounds were inactive (IC_50_ > 50 μM) against H1N1 influenza virus, indicating that both (*Z*)-deh-Phe and Leu or (*Z*)-deh-Leu moieties are necessary for anti-H1N1 activity (Figure 5). The dehydrogenation of Leu increases the activity, while the hydroxylation of deh-Phe or deh-Leu, the inversion of double bonds in deh-Phe and deh-Leu, and the *N*_1_-methylation reduce the activity. In addition, the new compounds **1**–**5** were tested for cytotoxicity against HL-60 and K562 cell lines by MTT method [23], and A549 cell lines by SRB method [24] and for anti-inflammatory effects by inhibition of LPS-mediated NF-κB transcription activity in RAW264.7 cells [25]. The antimicrobial activities of compounds **1**–**10** against *Escherichia coli*, *Enterobacter aerogenes*, *Pseudomonas aeruginosa*, *Bacillus subtilis*, *Staphylococcus aureus*, and *Candida albicans* were also evaluated by 2-fold dilution method [26]. The results (Table S1, Supplementary Information) showed that new compounds **1**–**5** did not exhibit cytotoxicity (IC_50_ > 100 μM) and anti-inflammatory effects (IC_50_ > 10 μM); compounds **1**–**10** did not show antimicrobial effects as well (MIC > 100 μg/mL). Although dehydro-DKPs have been reported to display diverse bioactivities, such as inhibition of protein tyrosine kinase [27], cell cycle arrest [28], inhibition of blood platelet aggregation [29], anti-bacteria [30], antitumor [30] and anti-inflammation [31], the antivirus effect on H1N1 was reported here for the first time. 

## 3. Experimental Section

### 3.1. General Experimental Procedures

Optical rotations were obtained on a JASCO P-1020 digital polarimeter. UV spectra were measured on a Beckman DU 640 spectrophotometer. IR spectra were recorded on a Nicolet Nexus 470 spectrophotometer as KBr disks. CD spectra were collected using a JASCO J-715 spectropolarimeter. NMR data of **1**, **2** and **4**–**6** were measured on a JEOL JNM-ECP 600 spectrometer, and chemical shifts were recorded as δ values. NMR spectra of **1a**, **1b**, **1c**, **2a**, **2b**, **3**, and NOESY spectra of all the compounds were recorded on a Bruker Avance 600 spectrometer. HRESIMS measurements were taken on a Q-TOF ULTIMA GLOBAL GAA076 LC mass spectrometer. Semipreparative HPLC was performed using an ODS column (YMC-pak ODS-A, Kyoto, Japan, 10 × 250 mm, 5 μm, 4.0 mL/min) and chiral separation was performed on chiral column (CHIRALPAK AY-H, Kyoto, Japan, 4.6 × 150 mm, 0.5 mL/min) by HPLC. Marfey’s analysis and C_3_ Marfey’s analysis were implemented using ODS column (YMC-pak ODS-A, 4.6 × 250 mm, 5 μm, 1.0 mL/min) and C_3_ column (Agilent Zorbax StableBond C_3_, Palo Alto, CA, USA, 4.6 × 150 mm, 5 μm, 1.0 mL/min), respectively. TLC and column chromatography (CC, 2.5 × 103 cm) were performed on plates precoated with silica gel GF_254_ (10–40 μm, Qingdao Marine Chemical Factory, Qingdao, China), and over Sephadex LH-20 (Amersham Biosciences, Uppsala, Sweden), respectively. Vacuum-liquid chromatography (VLC, 7 × 40 cm) utilized silica gel (200–300 mesh, Qingdao Marine Chemical Factory, Qingdao, China) and RP-18 (40–63 μm, Merck, Darmstadt, Germany). Sea salt used was made by the evaporation of seawater collected in Laizhou Bay (Weifang Haisheng Chemical Factory, Shangdong, China). Glucose (Shanghai Huixing Biochemical Reagent Co., Ltd., Shanghai, China); beef extract, yeast extract and peptone (Beinjing Shuangxuan Microbe Culture Medium Products Factory, Beijing, China); soluble starch (Beijng Aoboxing Universeen Bio-Tech Co., Ltd., Beijing, China); K_2_HPO_4_ (Tianjin Kermel Chemical Reagent Co., Ltd., Tianjin, China); MgSO_4_ (Shanghai Chemical Reagent Research Institute, Shanghai, China), and CaCO_3_ (Tianjijn Bodi Chemical Co., Ltd., Tianjin, China).

### 3.2. Actinomycete Material

The actinomycete strain *Streptomyces* sp. FXJ7.328 was isolated from coastal sediment collected at Huanghai beach (E 121.706°, N 39.007°), Dalian, China in January 2009. The sediment sample was dried at room temperature, suspended in sterile distilled water, serially diluted, heated in a water bath at 55 °C for 10 min, and spread-plated on oatmeal agar plates (ISP3, medium 3 of the International *Streptomyces* Project) [32]. After four weeks of incubation at 28 °C, the strain was purified on yeast extract-malt extract agar (ISP2, medium 2 of the International *Streptomyces* Project) [32], and was identified as a member of the genus *Streptomyces* on the basis of 16S rRNA gene sequence analysis. Genomic DNA isolation, PCR amplification of 16S rRNA gene and sequence alignment of the strain were performed as described previously [33]. Its 16S rRNA gene sequence (GenBank access No. JF346514) showed 99% similarity with type strains of *Streptomyces albus* subsp. *albus* (AB184781), *Streptomyces almquistii* (AB184258), *Streptomyces flocculus* (DQ442498), *Streptomyces gibsonii* (NR_041180) and *Streptomyces rangoonensis* (NR_041110). The producing strain was prepared on ISP3 medium and stored in Huang’s Lab at 4 °C. 

### 3.3. Fermentation and Extraction

The spores of *Streptomyces* sp. FXJ7.328 were directly cultured in 500 mL Erlenmeyer flasks containing 150 mL fermentation media consisted of 2% glucose, 0.3% beef extract, 1% yeast extract, 1% soluble starch, 1% peptone, 0.05% K_2_HPO_4_, 0.05% MgSO_4_, 0.2% CaCO_3_, and 3.3% sea salt (pH 7.0). The cultures were incubated on a rotatory shaker at 180 rpm at 28 °C for eight days. The whole fermentation broth (120 L) was divided into three equal parts that were extracted three times with equal volumes of EtOAc separately. The EtOAc solutions were combined and evaporated under reduced pressure to give a dark brown gum (32.5 g). 

### 3.4. Purification and Identification

The EtOAc extract (32.5 g) was subjected to SiO_2_ VLC eluting with CH_2_Cl_2_-petroleum ether (0%–100%), and then with MeOH-CH_2_Cl_2_ (0%–50%), to give nine fractions (Fr.1–Fr.9). Fraction 3 (3.82 g) was separated into three subfractions by gel filtration over Sephadex LH-20 with CH_3_OH/CH_2_Cl_2_ (1:1). Fraction 3-2 (442 mg) was further subjected to HPLC separation eluting with 70% MeOH to yield **7** (5.5 mg, *t*_R_ = 7.20 min, 0.046 mg/L), **8** (20 mg, *t*_R_ = 11.5 min, 0.17 mg/L), and **10** (3 mg, *t*_R_ = 9.5 min, 0.025 mg/L). Fraction 4 (3.2 g) and Fraction 5 (2.78 g) were both separated into five parts (Fr.4-1–Fr.4-5 and Fr.5-1–Fr.5-5) by RP-18 column chromatography, eluting with CH_3_OH/H_2_O (5%–100%). Fraction 4-2 (512 mg) was further separated by Sephadex LH-20 with CH_3_OH/CH_2_Cl_2_ (1:1) to give four subfractions (Fr.4-2-1–Fr.4-2-4). Compound **3** (7 mg, 0.058 mg/L) was purified from the precipitate of Fr.4-2-2 (135 mg) after washing with MeOH, and compound **9** (6 mg, *t*_R_ = 7.4 min, 0.05 mg/L) was obtained from Fr.4-2-3 (163 mg) by HPLC purification eluting with 60% MeOH. Fraction 4-4 (332.7 mg) was separated by Sephadex LH-20 with CH_3_OH/CH_2_Cl_2_ (1:1) to afford four subfractions (Fr.4-4-1–Fr.4-4-4). Fr.4-4-2 (94 mg) was also submitted to HPLC purification on ODS column eluting with 70% MeOH to yield a racemic mixture of **1**/**2** with an excess of one enantiomer (11 mg, *t*_R_ = 5.59 min, 0.092 mg/L) which showed two peaks (1:2) by HPLC analysis on a chiral column (CHIRALPAK AY-H, Figure S37, Supplementary Information). Then the chiral separation by HPLC (Amylose tris(5-chloro-2-methylphenylcarbamate)) with EtOH yielded **1** (7 mg, 12.12 min, 0.058 mg/L) and **2** (3 mg, 8.82 min, 0.025 mg/L). Then Fr.4-4-3 (120 mg) was further subjected to HPLC separation eluted with 60% MeOH to yield **5** (3 mg, *t*_R_ = 10.4 min, 0.025 mg/L) and **6** (4.7 mg, *t*_R_ = 11.64 min, 0.039 mg/L). Fraction 5-2 (245 mg) was further purified by Sephadex LH-20 with MeOH to produce four subfractions (Fr.5-2-1–Fr.5-2-4). Compound **4** (2.5 mg, *t*_R_ = 14.7 min, 0.021 mg/L) was obtained from Fr.5-2-3 (63 mg) by HPLC with 45% MeOH. 

**Compound** **1**: Yellow solid; UV (MeOH) λ_max_ (log ε): 238 (4.10), 323 (4.51) nm; [α]^25^_D_ −28 (*c* 0.05, CH_3_OH), IR (KBr) ν_max_ 3496, 3208, 3073, 3022, 1675, 1617, 1494, 1454, 1376, 1033, 997, 763, 688 cm^−1^; ^1^H and ^13^C NMR data, Table 1. 

**Compound** **2**: Yellow solid; UV (MeOH) λ_max_ (log ε): 238 (4.10), 323 (4.51) nm; [α]^25^_D_ +28 (*c* 0.05, CH_3_OH), IR (KBr) ν_max_ 3496, 3208, 3073, 3022, 1675, 1617, 1494, 1454, 1376, 1033, 997, 763, 688 cm^−1^; ^1^H and ^13^C NMR data, Table 1.

**Compound** **3**: Yellow amorphous solid; UV (MeOH) λ_max_ (log ε): 248 (3.75), 347 (4.10) nm; IR (KBr) ν_max_ 3183, 3073, 2960, 2867, 1681, 1639, 1606, 1541, 1421, 1359, 1275, 1173, 1024, 998, 829, 761 cm^−1^; ^1^H and ^13^C NMR data, Table 1; HRESIMS *m*/*z* 273.1231 [M + H]^+^ (calcd. for C_1__5_H_1__7_N_2_O_3_ 273.1234).

**Compound** **4**: Yellow solid; UV (MeOH) λ_max_ (log ε): 250 (3.71), 347 (4.25) nm; IR (KBr) ν_max_ 2973, 2937, 2879, 1708, 1638, 1577, 1459, 1379, 1273, 1122, 1092, 1025, 960 cm^−1^; ^1^H and ^13^C NMR data, Table 1; HRESIMS *m*/*z* 247.1189 [M + H]^+^ (calcd. for C_12_H_1__5_N_4_O_2_ 247.1190).

**Compound** **5**: White solid; UV (MeOH) λ_max_ (log ε): 229 (4.05), 302 (4.17) nm; [α]^25^_D_ −36 (*c* 0.66, CH_3_OH), IR (KBr) ν_max_ 3168, 3077, 3042, 2961, 2867, 1680, 1646, 1546, 1426, 1392, 1357, 1092, 933, 800, 760, 619 cm^−1^; ^1^H and ^13^C NMR data, Table 1; HRESIMS *m*/*z* 259.1439 [M + H]^+^ (calcd. for C_15_H_19_N_2_O_2_ 259.1441).

**(3*Z*,6*S*)-3-Benzylidene-6-isobutylpiperazine-2,5-dione (6)**: [α]^25^_D_ −54 (*c* 0.3, DMSO); ^1^H NMR (600 MHz, DMSO-*d*_6_): δ 8.58 (1H, s, NH-1), 9.98 (1H, s, NH-4), 3.94 (1H, m, H-6), 1.58 (2H, m, H-7), 1.80 (1H, m, H-8), 0.89 (3H, d, *J* = 6.1, H-9/10), 0.88 (3H, d, *J* = 5.5, H-9/10), 6.67 (1H, s, H-11), 7.48 (2H, d, *J* = 7.7, H-13/17), 7.39 (2H, t, *J* = 7.7, H-14/16), 7.30 (1H, t, *J* = 7.7, H-15). ^13^C NMR (150 MHz, DMSO-*d*_6_): δ 161.0 (C_q_, C-2), 127.3 (C_q_, C-3), 168.1 (C_q_, C-5), 54.2 (CH, C-6), 44.2 (CH_2_, C-7), 24.1 (CH, C-8), 23.3 (CH_3_, C-9), 22.6 (CH_3_, C-10), 114.9 (CH, C-11), 133.9 (C_q_, C-12), 129.2 × 2 (CH, C-13/17), 129.8 × 2 (CH, C-14/16), 128.5 (CH, C-15). HRESIMS *m*/*z* 259.1456 [M + H]^+^ (calcd. for C_15_H_19_N_2_O_2_ 259.1441).

### 3.5. Preparation of *p*-Bromobenzoate (**1c**) of Compound **1** [14]

Compound **1** (2 mg, 6.99 μmol) was dissolved in 1 mL of CH_2_Cl_2_, and triethylamine (10 μL) and *p*-bromobenzoyl chloride (20 mg, 92.2 μmol) were added. The mixture was stirred for 7 h at room temperature. Then 2 mL of H_2_O were added and the solution was extracted three times with CH_2_Cl_2_ (5 mL each). The CH_2_Cl_2_ solutions were combined and evaporated under reduced pressure to give a gum. *p*-Bromobenzoate **1c** (2 mg, 4.27 μmol, 7.9 min, 61% yield) was obtained by HPLC purification eluting with 85% (MeOH-H_2_O).

***p*-Bromobenzoate (1c)**: Yellow solid; [α]^25^_D_ −50 (*c* 0.1, CH_3_OH); CD (*c* 0.11, MeOH) λ_ext_ (Δε) 313 (−5.2), 254 (+4.78), 208 (−1.73). ^1^H NMR (600 MHz, DMSO-*d*_6_): δ 3.18 (3H, s, 1-NCH_3_), 5.57 (d, *J* = 9.5 Hz, H-7), 4.09 (m, H-8), 4.20 (dd, *J* = 7.3, 10.3 Hz, H-9a), 4.28 (dd, *J* = 6.0, 10.4 Hz, H-9b), 1.14 (3H, d, *J* = 6.7 Hz, CH_3_-10), 6.71 (s, H-11), 7.53 (2H, d, *J* = 7.6 Hz, H-13/17), 7.38 (2H, t, *J* = 7.5 Hz, H-14/16), 7.30 (t, *J* = 7.4 Hz, H-15), 7.86 (2H, d, *J* = 8.5 Hz, H-2′/6′ in *p*-BrC_6_H_4_CO_2_-), 7.67 (2H, d, *J* = 8.5 Hz, H-2′/6′ in *p*-BrC_6_H_4_CO_2_-). ^13^C NMR (150 MHz, DMSO-*d*_6_): δ 31.3 (CH_3_, 1-NCH_3_), 158.5 (C_q_, C-2), 126.0 (C_q_, C-3), 158.8 (C_q_, C-5), 131.1 (C_q_, C-6), 130.4 (CH, C-7), 31.4 (CH, C-8), 69.3 (CH_2_, C-9), 17.5 (CH_3_, C-10), 116.6 (CH, C-11), 133.5 (C_q_, C-12), 129.7 × 2 (CH, C-13/17), 129.1 × 2 (CH, C-14/16), 128.0 (CH, C-15), 165.6 (C_q_, -CO_2_- in *p*-BrC_6_H_4_CO_2_-), 129.4 (C_q_, C-1′ in *p*-BrC_6_H_4_CO_2_-), 132.3 × 2 (CH, C-2′/6′ in *p*-BrC_6_H_4_CO_2_-), 131.5 × 2 (CH, C-3′/5′ in *p*-BrC_6_H_4_CO_2_-), 127.9 (C_q_, C-4′ in *p*-BrC_6_H_4_CO_2_-). ESIMS *m*/*z* 469.1 and 471.1; HRESIMS *m*/*z* 469.0755 [M + H]^+^ (calcd. for C_23_H_2__2_^79^BrN_2_O_4_ 469.0758).

### 3.6. Preparation of S-MTPA and R-MTPA Esters **1a**, **1b**, **2a**, and **2b** of Compounds **1** and **2** [15,16]

Compound **1** (1 mg, 3.50 μmol) was dissolved in 500 μL of anhydrous pyridine and 4-dimethyl-aminopyridine (3 mg, 24.6 μmol) and (*R*)-MTPACl (10 μL) were added. The reaction was stirred for 12 h at room temperature. Then 1 mL of H_2_O was added, and the solution was extracted three times with CH_2_Cl_2_ (5 mL each). After removal of CH_2_Cl_2_ under reduced pressure, the residue was purified by semipreparative HPLC (70% MeOH-H_2_O) to yield (*S*)-MTPA ester **1a** (1.2 mg, 2.39 μmol, *t*_R_ = 27.11 min, 68% yield). By the same procedure, (*R*)-MTPA ester **1b** (1.1 mg, 2.19 μmol, *t*_R_ = 24.74 min, 63% yield), (*S*)-MTPA ester **2a** (0.8 mg, 1.59 μmol, *t*_R_ = 24.74 min, 45% yield) and (*R*)-MTPA ester **2b** (0.9 mg, 1.79 μmol, *t*_R_ = 27.11 min, 51% yield) were obtained from the reaction of **1** and **2** (1 mg, 3.50 μmol each) with (*S*)-MTPACl, (*R*)-MTPACl and (*S*)-MTPACl (10 μL each), respectively. 

**(*S*)-MTPA ester 1a**: White solid. ^1^H NMR (600 MHz, DMSO-*d*_6_): δ 3.07 (3H, s, 1-NCH_3_), 3.99 (m, H-8), 1.06 (3H, d, *J* = 6.8 Hz, H-10), 5.51 (d, *J* = 9.6 Hz, H-7), 6.76 (s, H-11), 4.36 (dd, *J* = 5.6, 10.5 Hz, H-9a), 4.28 (dd, *J* = 7.4, 10.4 Hz, H-9b). 7.51 (2H, d, *J* = 7.5 Hz, H-13/17), 7.41 (2H, t, *J* = 7.6 Hz, H-14/16), 7.32 (t, *J* = 7.4 Hz, H-15). HRESIMS *m*/*z* 525.1612 [M + Na]^+^ (calcd. for C_2__6_H_2__5_F_3_N_2_O_5_Na 525.1608).

**(*R*)-MTPA ester 1b**: White solid. ^1^H NMR (600 MHz, DMSO-*d*_6_): δ 3.12 (3H, s, 1-NCH_3_), 3.99 (m, H-8), 1.07 (3H, d, *J* = 6.8 Hz, H-10), 5.51 (d, *J* = 9.6 Hz, H-7), 6.76 (s, H-11), 4.33 (2H, d, *J* = 6.5 Hz, H-9), 7.50 (2H, d, *J* = 7.6 Hz, H-13/17), 7.41 (2H, t, *J* = 7.6 Hz, H-14/16), 7.32 (t, *J* = 7.4 Hz, H-15). HRESIMS *m*/*z* 525.1613 [M + Na]^+^ (calcd. for C_2__6_H_2__5_F_3_N_2_O_5_Na 525.1608). 

**(*S*)-MTPA ester 2a**: White solid. ^1^H NMR (600 MHz, DMSO-*d*_6_): δ 3.12 (3H, s, 1-NCH_3_), 3.99 (m, H-8), 1.07 (3H, d, *J* = 6.8 Hz, H-10), 5.51 (d, *J* = 9.6 Hz, H-7), 6.76 (s, H-11), 4.33 (2H, d, *J* = 6.5 Hz, H-9), 7.50 (2H, d, *J* = 7.6 Hz, H-13/17), 7.41 (2H, t, *J* = 7.6 Hz, H-14/16), 7.32 (t, *J* = 7.4 Hz, H-15). HRESIMS *m*/*z* 525.1607 [M + Na]^+^ (calcd. for C_2__6_H_2__5_F_3_N_2_O_5_Na 525.1608).

**(*R*)-MTPA ester 2b**: White solid. ^1^H NMR (600 MHz, DMSO-*d*_6_): δ 3.07 (3H, s, 1-NCH_3_), 3.98 (m, H-8), 1.06 (3H, d, *J* = 6.8 Hz, H-10), 5.51 (d, *J* = 9.6 Hz, H-7), 6.76 (s, H-11), 4.35 (dd, *J* = 5.6, 10.5 Hz, H-9a), 4.27 (dd, *J* = 7.4,10.4 Hz, H-9b), 7.50 (2H, d, *J* = 7.5 Hz, H-13/17), 7.41 (2H, t, *J* = 7.6 Hz, H-14/16), 7.32 (t, *J* = 7.4 Hz, H-15). HRESIMS *m*/*z* 525.1606 [M + Na]^+^ (calcd. for C_2__6_H_2__5_F_3_N_2_O_5_Na 525.1608). 

### 3.7. Preparation of FDAA Derivatives of the Acid Hydrolysates of **5** and Four Authentic Isoleucine Samples (l-, l-*allo*-, d- and d-*allo*-) and Marfey’s Analysis [18] and C_3_ Marfey’s Analysis [19,20]

Compound **5** (1 mg, 3.88 μmol) was dissolved in 6 M HCl (1 mL) in a sealed tube and the mixture was heated at 105 °C for 17 h. Then the solution was cooled and evaporated to dryness. The residue, l-Ile, l-*allo*-Ile and d-Ile, d-*allo*-Ile, was dissolved in H_2_O (250 μL each), respectively. 50 μL of each solution was treated with 200 μL of 1% FDAA in acetone followed by 1.0 M NaHCO_3_ (40 μL). The reaction was maintained 1 h at 45 °C and then quenched by addition of 2.0 M HCl (10 μL). The corresponding FDAA derivatives of hydrolysate of **5**, l-Ile, l-*allo*-Ile, d-Ile and d-*allo*-Ile were analyzed by ODS HPLC column maintained at 30 °C using the following programs: solvent A, H_2_O + 0.2% TFA; solvent B, MeCN; linear gradient, 0 min 25% B (75% A), 40 min 60% B (40% A), 45 min 100% B ; UV detection at 340 nm. The retention times for the FDAA derivatives of the hydrolysate of **5**, l-Ile, l-*allo*-Ile, d-Ile and d-*allo*-Ile were 23.06, 23.06, 23.06, 28.11 and 28.11 min, respectively (Figure S38). The FDAA derivatives of the hydrolysate of **5**, l-Ile, and l-*allo*-Ile were further analyzed by C_3_ HPLC column maintained at 50 ^o^C. The column was developed with a linear gradient of 15%–60% MeOH/water (+isocratic 5% of a 1% formic acid solution in MeCN) over 55 min with UV detection at 340 nm. The retention times for the FDAA derivatives of the hydrolysate of **5**, standard l-Ile, and l-*allo*-Ile, were 38.50, 38.50, and 37.49 min, respectively (Figure S39).

### 3.8. Bioassays

Cytotoxicity was assayed by the MTT [23] and SRB method [24]. In the MTT assay, HL-60 cell and K562 cell line were cultured in RPMI-1640 supplemented with 10% FBS under a humidified atmosphere of 5% CO_2_ and 95% air at 37 °C, 198 μL of cell suspensions with a density of 4.6 × 10^4^ cells mL^−1^ was plated in 96-well microtiter plates and incubated for 24 h. Then, 2 μL of the test solutions in MeOH were added to each well and further incubated for 36 h. The MTT solution (20 μL, 5 mg/mL in IPMI-1640 medium) was then added to each well and incubated for 4 h. Old medium containing MTT (150 μL) was then gently replaced by DMSO and pipetted to dissolve any formazan crystals formed. Absorbance was then determined on a Spectra Max Plus plate reader at 570 nm. In the SRB assay, 200 μL of the A549 cell suspension was plated in 96-well plates at density of 2 × 10^5^ cell mL^−1^. Then, 2 μL of the test solutions (in MeOH) were added to each well and the culture was further incubated for 24 h. The cells were fixed with 12% trichloroacetic acid and the cell layer stained with 0.4% SRB. The absorbance of the SRB solution was measured at 515 nm. Adriamycin was used as positive control (IC_50_ 0.652 μM, 0.645 μM and 0.080 μM for HL-60, K562 and A549 cell, respectively).

The antiviral activity against H1N1 was evaluated by the CPE inhibition assay [21,22]. Confluent MDCK cell monolayers were firstly incubated with influenza virus (A/Puerto Rico/8/34 (H1N1), PR/8) at 37 °C for 1 h. After removing the virus dilution, cells were maintained in infecting media (RPMI 1640, 4 μg/mL of trypsin) containing different concentrations of test compounds at 37 °C. After 48 h incubation at 37 °C, the cells were fixed with 100 μL of 4% formaldehyde for 20 min at room temperature. After removal of the formaldehyde, the cells were stained with 0.1% crystal violet for 30 min. The plates were washed and dried, and the intensity of crystal violet staining for each well was measured in a microplate reader (Bio-Rad, Hercules, CA, USA) at 570 nm. The IC_50_ was calculated as the compound concentration required inhibiting influenza virus yield at 48 h post-infection by 50%. Ribavirin was used as the positive control with an IC_50_ value of 38.8 μM.

The anti-inflammatory effects of compounds were assayed by inhibition of lipopolysaccharide (LPS)-mediated NF-κB transcriptional activity in RAW 264.7 cells [25]. RAW 264.7 cells (2.5 × 10^5^ cells/well) were placed in a 24-well plate. The cells were then stably transfected with pNF-κB-Luc expression plasmid (0.5 μg/well). Transfections were performed using lipofectamine 2000 in accordance with the instructions of the manufacturer (Invitrogen, Carlsbad, NM, USA). Stably transfected cells were pretreated 2 h with the test compounds and stimulated with 0.1 μg/mL of LPS for an additional 4 h. The luciferase assay was performed with the aid of a Steady-Glo Luciferase assay system in accordance with the instructions of the manufacturer (Promega, Madison, WI, USA).

The antimicrobial activities against *E*. *coli*, *E*. *aerogenes*, *P*. *aeruginosa*, *B*. *subtilis*, *S*. *aureus*, and *C*. *albicans* were evaluated by 2-fold dilution method [26]. The tested strains were cultivated on LB broth for bacteria and in YPD broth for *C*. *albicans* at 37 °C. The test compounds were dissolved in DMSO at different concentrations from 100 to 0.78 μg/mL (from 6.25 to 0.025 μg/mL for the positive controls) by the continuous 2-fold dilution methods. The minimum inhibitory concentrations (MICs) were determined in 96-well plates, and each well contains 100 μL of contents composed of 20 μL of inoculums (5 × 10^5^ CFU/mL), test compounds and LB or YPD media. The microtiter plates were incubated at 35 °C for 24 h and were examined for microbes’ growth by turbidity in daylight. The MICs were defined as the lowest concentration at which no visible growth of microbes could be observed. Ciprofloxacin lactate and ketoconazole were used as positive controls for *E*. *coli*, *E*. *aerogenes*, *P*. *aeruginosa*, *B*. *subtilis*, *S*. *aureus*, and *C*. *albicans* with MIC values of 0.05, 0.19, 0.1, 0.39, 3.12 and 0.025 μg/mL, respectively. 

## 4. Conclusions

Five new vinylidene substituted diketopiperazines (DKPs) **1**–**5** were isolated and their structures including absolute configurations were determined. The new compound **3** displayed modest activity and the known analogs **6** and **7** displayed potent activity against H1N1 virus with IC_50_ values of 41.5 ± 4.5, 28.9 ± 2.2 and 6.8 ± 1.5 μM, respectively. The results showed that both *Z*-deh-Phe and Leu or *Z*-deh-Leu substitutions significantly increase the anti-H1N1 activity of DKPs, while *E*-isomerization and hydroxylation of both deh-Phe and deh-Leu moieties, and *N*-methylation reduce the activity. The substitutions of both deh-Phe or deh-Tyr with deh-His and Leu or deh-Leu with *iso*-Leu also reduce the anti-H1N1 activity. In addition, the ^13^C NMR data of (3*Z*,6*S*)-3-benzylidene-6-isobutylpiperazine-2,5-dione (**6**) was reported here for the first time. 

## Supplementary Files

Supplementary File 1 Supplementary Information (PDF, 4268 KB)
