# Supplementary material for: Diketopiperazine Derivatives from the Marine-Derived Actinomycete Streptomyces sp. FXJ7.328
Source: Mar Drugs. 2013 Mar 28;11(4):1035–49. doi: 10.3390/md11041035 (PMC3705386; doi:10.3390/md11041035)
Supplement: Supplementary File 1 — Supplementary Information (PDF, 4268 KB) [file marinedrugs-11-01035-s001.pdf]

Supplementary Information

Table of Contents

The 16S rRNA gene sequences data of *Streptomyces* sp. FXJ7.328 3
The physicochemical data of the known compounds **7–10** 3
Figure S1. The ^{1}H NMR (600 MHz, DMSO-d_6) spectrum of compounds **1** and **2** 5
Figure S2. The ^{13}C NMR (150 MHz, DMSO-d_6) spectrum of compounds **1** and **2** 6
Figure S3. The DEPT (150 MHz, DMSO-d_6) spectrum of compounds **1** and **2** 7
Figure S4. The ^{1}H-^{1}H COSY (600 MHz, DMSO-d_6) spectrum of compounds **1** and **2** 8
Figure S5. The HMQC (600 MHz, DMSO-d_6) spectrum of compounds **1** and **2** 9
Figure S6. The HMBC (600 MHz, DMSO-d_6) spectrum of compounds **1** and **2** 10
Figure S7. The NOESY (600 MHz, DMSO-d_6) spectrum of compounds **1** and **2** 11
Figure S8. The ^{1}H NMR (600 MHz, DMSO-d_6) spectrum of compound **3** 12
Figure S9. The ^{13}C NMR (150 MHz, DMSO-d_6) spectrum of compound **3** 13
Figure S10. The DEPT (150 MHz, DMSO-d_6) spectrum of compound **3** 14
Figure S11. The HMQC (600 MHz, DMSO-d_6) spectrum of compound **3** 15
Figure S12. The ^{1}H-^{1}H COSY (600 MHz, DMSO-d_6) spectrum of compound **3** 16
Figure S13. The HMBC (600 MHz, DMSO-d_6) spectrum of compound **3** 17
Figure S14. The NOESY (600 MHz, DMSO-d_6) spectrum of compound **3** 18
Figure S15. The ^{1}H NMR (600 MHz, DMSO-d_6) spectrum of compound **4** 19
Figure S16. The ^{13}C NMR (150 MHz, DMSO-d_6) spectrum of compound **4** 20
Figure S17. The DEPT (150 MHz, DMSO-d_6) spectrum of compound **4** 21
Figure S18. The ^{1}H-^{1}H COSY (600 MHz, DMSO-d_6) spectrum of compound **4** 22
Figure S19. The HMQC (600 MHz, DMSO-d_6) spectrum of compound **4** 23
Figure S20. The HMBC (600 MHz, DMSO-d_6) spectrum of compound **4** 24
Figure S21. The ^{1}H NMR (600 MHz, DMSO-d_6) spectrum of compound **5** 25
Figure S22. The ^{13}C NMR (150 MHz, DMSO-d_6) spectrum of compound **5** 26
Figure S23. The DEPT (150 MHz, DMSO-d_6) spectrum of compound **5** 27
Figure S24. The ^{1}H-^{1}H COSY (600 MHz, DMSO-d_6) spectrum of compound **5** 28
Figure S25. The HMQC (600 MHz, DMSO-d_6) spectrum of compound **5** 29
Figure S26. The HMBC (600 MHz, DMSO-d_6) spectrum of compound **5** 30
Figure S27. The NOESY (600 MHz, DMSO-d_6) spectrum of compound **5** 31
Figure S28. The ^{1}H NMR (600 MHz, DMSO-d_6) spectrum of compound **6** 32
Figure S29. The ^{13}C NMR (150 MHz, DMSO-d_6) spectrum of compound **6** 33
Figure S30. The DEPT (150 MHz, DMSO-d_6) spectrum of compound **6** 34
Figure S31. The ^{1}H NMR (600 MHz, DMSO-d_6) spectrum of (*S*)-**1** MTPA ester (**1a**) 35
Figure S32. The ^{1}H NMR (600 MHz, DMSO-d_6) spectrum of (*R*)-**1** MTPA ester (**1b**) 36
Figure S33. The ^{1}H NMR (600 MHz, DMSO-d_6) spectrum of (*S*)-**2** MTPA ester (**2a**) 37
Figure S34. The ^{1}H NMR (600 MHz, DMSO-d_6) spectrum of (*R*)-**2** MTPA ester (**2b**) 38
Figure S35. The ^{1}H NMR (600 MHz, DMSO-d_6) spectrum of *p*-bromobenzoate (**1c**) 39

Figure S36. The DEPTQ (150 MHz, DMSO-d_6) spectrum of *p*-bromobenzoate (**1c**) 40
Figure S37. The resolution of mixture of **1** and **2** by chiral column 41
Figure S38. Marfey's method applying for compound **5** 42
Figure S39. C_3 Marfey's method applying for compound **5** 43
Figure S40. Dose-response histograms of antivirus activity for compound **3** and Ribavirin 44
Table S1. Cytotoxic, anti-virus, anti-microbial and anti-inflammatory activities of **1**–**10** 44
Table S2. The HMBC correlations (H → C) for compounds **1**–**5** 45

The 16S rRNA Gene Sequences Data of *Streptomyces* sp. FXJ7.328

TCGAAGAAGAAAGCCGCTTCGGTGGTGGATTAGTGGCGAACGGGTGAGTAACACGTGGG
CAATCTGCCCTGCACTCTGGGACAAGCCCTGGAAACGGGTCTAATACCGGATATGACACG
GGATCGCATGGTCCGTGTCTGGAAAGCTCCGGCGGTGCAGGATGAGCCCGGGCCTATCAC
CTTGTTGGTGGGGTGATGGCCTACCAAGGCGACGACGGGTAGCCGGCCTGAGAGGGCG
ACCGGCCACACTGGGACTGAGACACGGCCCAGACTCCTACGGGAGGCAGCAGTGGGGAA
TATTGCACAATGGGCGCAAGCCTGATGCAGCGACGCCGCGTGAGGGATGACGGCCTTCG
GGTTGTAAACCTCTTTCAGCAGGGAAGAAGCGCGAGTGACGGTACCTGCAGAAGAAGCA
CCGGCTAACTACGTGCCAGCAGCCGCGGTAATACGTAGGGTGCGAGCGTTGTCCGGAATT
ATTGGGCGTAAAGAGCTCGTAGGCGGCTTGTCGCGTCGGATGTGAAAGCCCGGGGCTTAA
CCCCGGGTCTGCATTCGATACGGGCAGGCTAGAGTTCGGCAGGGGAGATTGGAATTCCTG
GTGTAGCGGTGAAATGCGCAGATATCAGGAGGAACACCGGTGGCGAAGGCGGATCTCTG
GGCCGATACTGACGCTGAGGAGCGAAAGCGTGGGGAGCGAACAGGATTAGATACCCTGG
TAGTCCACGCCGTAAACGTTGGGCACTAGGTGTGGGCGGCATTCCACGTCGTCCGTGCCG
CAGCTACGCATTAAGATGCCCCGCCTGGGGAGTACGGCCGCAAGGCTAAAACTCAAAGG
AATTGACGGGGGCCCGCACAAGCGGCGGAGCATGTGGCTTAATTCGACGCAACGCGAAG
AACCTTACCAAGGCTTGACATACACCGGAAAGCCGTAGAGATACGGCCCCCCTTGTGGTC
GGTGTACAGGTGGTGCATGGCTGTCGTCAGCTCGTGTCGTGAGATGTTGGGTTAAGTCCC
GCAACGAGCGCAACCCTT GTCCTGTGTTGCCAGCAACTCCTTTC

The Physicochemical Data of the Known Compounds 7–10

Compound 7: White solid; ^{1}H NMR (600 MHz, C_5H_5N-d_5): δ 11.33 (1H, s, H-1), 10.97 (1H, s, H-4), 6.32 (1H, d, J = 10.4 Hz, H-7), 3.21 (1H, m, H-8), 1.09 (6H, d, J = 6.6 Hz, CH_3-9/10), 7.32 (1H, s, H-11), 7.65 (2H, d, J = 7.6 Hz, H-13/17), 7.33 (2H, t, J = 7.6 Hz, H-14/16), 7.24 (1H, t, J = 7.3 Hz, H-15). ^{13}C NMR (150 MHz, C_5H_5N-d_5): δ 156.9 (C_q, C-2), 126.5 (C_q, C-3), 157.1 (C_q, C-5), 125.1 (C_q, C-6), 124.0 (C_q, C-7), 23.8 (CH, C-8), 20.7 × 2 (CH_3, C-9/10), 113.1 (CH, C-11), 133.0 (C_q, C-12), 127.6 × 2 (CH, C-13/17), 127.9 × 2 (CH, C-14/16), 126.6 (CH, C-15). ESIMS m/z 257 $[M + H]^+$.

Compound 8: Yellow solid; ^{1}H NMR (600 MHz, $CDCl_3$): δ 3.29 (3H, s, NCH_3-1), 8.02 (1H, s, H-4), 5.49 (1H, d, J = 9.66 Hz, H-7), 3.76 (1H, m, H-8), 1.08 (6H, d, J = 6.6 Hz, CH_3-9/10), 6.98 (1H, s, H-11), 7.38 (2H, d, J = 7.5 Hz, H-13/17), 7.41 (2H, t, J = 7.4 Hz, H-14/16), 7.32 (1H, t, J = 7.4 Hz, H-15). ^{13}C NMR (150 MHz, $CDCl_3$): δ 31.1 (CH_3, NCH_3-1), 157.8 (C_q, C-2), 125.8 (C_q, C-3), 157.6 (C_q, C-5), 128.1 (C_q, C-6), 134.8(C_q, C-7), 26.8 (CH, C-8), 24.4 × 2 (CH_3, C-9/10), 115.8(CH, C-11), 133.4 (C_q, C-12), 128.5 × 2 (CH, C-13/17), 129.5 × 2 (CH, C-14/16), 128.6 (CH, C-15). ESIMS m/z 271 $[M + H]^+$.

Compound 9: White solid; $[\alpha]^{25}_D$ −88 (c 0.03, DMSO-d_6), ^{1}H NMR (600 MHz, DMSO-d_6): δ 8.50 (1H, s, NH-1), 9.96 (1H, s, NH-4), 3.78 (1H, t, J = 3.8 Hz, H-6), 2.07 (1H, m, H-7), 0.93 (3H, d, J = 6.8 Hz, CH_3-8), 0.87 (3H, d, J = 6.6 Hz, CH_3-9), 6.66 (1H, s, H-10), 7.45 (2H, d, J = 7.6 Hz, H-12/16), 7.39 (2H, t, J = 7.6 Hz, H-13/15), 7.29 (1H, t, J = 7.4 Hz, H-14). ^{13}C NMR (150 MHz, DMSO-d_6): δ 161.2 (C_q, C-2), 127.1 (C_q, C-3), 167.2 (C_q, C-5), 61.1 (CH, C-6), 34.1 (CH, C-7), 18.7 (CH_3, C-8), 17.5 (CH_3, C-9), 114.9 (CH, C-10), 133.8 (C_q, C-11), 129.6 × 2 (CH, C-12/16), 129.3 × 2 (CH, C-13/15), 128.6 (CH, C-14). ESIMS m/z 245 $[M + H]^+$.

Compound 10: Yellow solid; ^{1}H NMR (600 MHz, $CDCl_3$): δ 3.21 (3H, s, NCH_3-1), 9.67 (1H, s, H-4), 5.47 (1H, d, J = 10.4 Hz, H-7), 3.81 (1H, m, H-8), 1.10 (6H, d, J = 6.7 Hz, CH_3-9/10), 6.46 (1H, s, H-11), 7.52 (2H, d, J = 7.6 Hz, H-13/17), 7.33 (2H, t, J = 7.6 Hz, H-14/16), 7.27 (1H, t, J = 7.4 Hz, H-15). ^{13}C NMR (150 MHz, $CDCl_3$): δ 31.1 (CH_3, NCH_3-1), 156.9 (C_q, C-2), 125.4 (C_q, C-3), 159.9 (C_q, C-5), 128.1 (C_q, C-6), 134.5 (CH, C-7), 26.8 (CH, C-8), 23.4 × 2 (CH_3, C-9/10), 122.4 (CH, C-11), 134.1 (C_q, C-12), 127.9 × 2 (CH, C-13/17), 130.1 × 2 (CH, C-14/16), 128.4 (CH, C-15). ESIMS m/z 271 $[M + H]^+$.

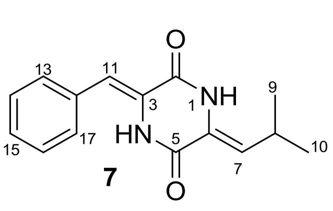

7

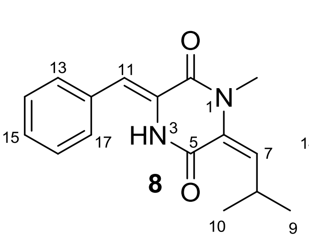

8

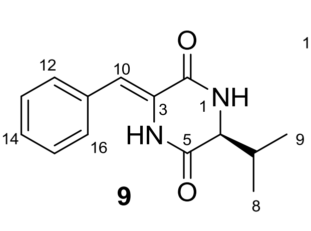

9

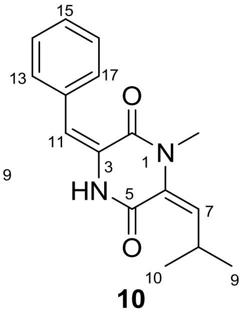

10

Figure S1. The ^{1}H NMR (600 MHz, DMSO-d_6) spectrum of compounds **1** and **2**.

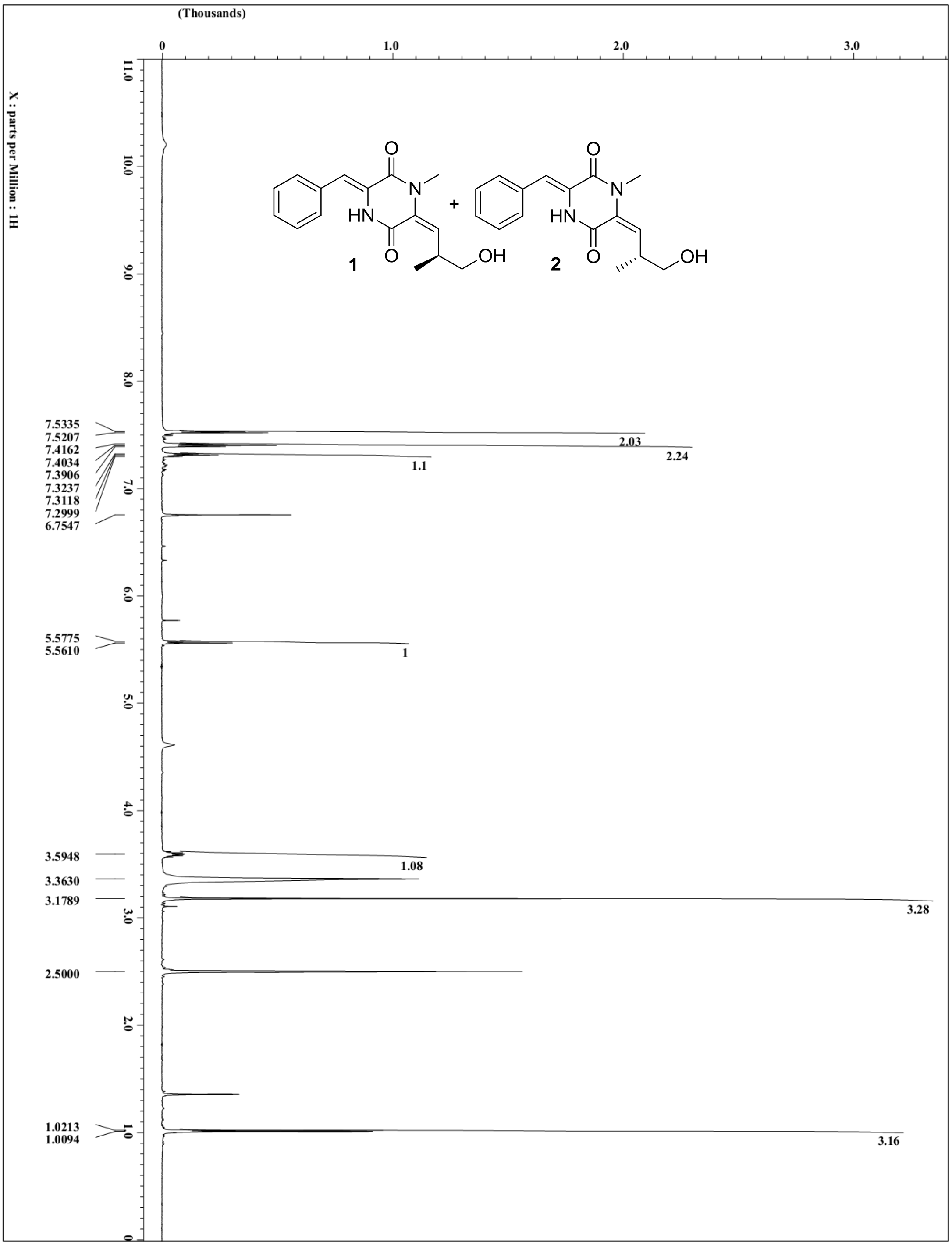

Figure S2. The ^{13}C NMR (150 MHz, DMSO-d_6) spectrum of compounds **1** and **2**.

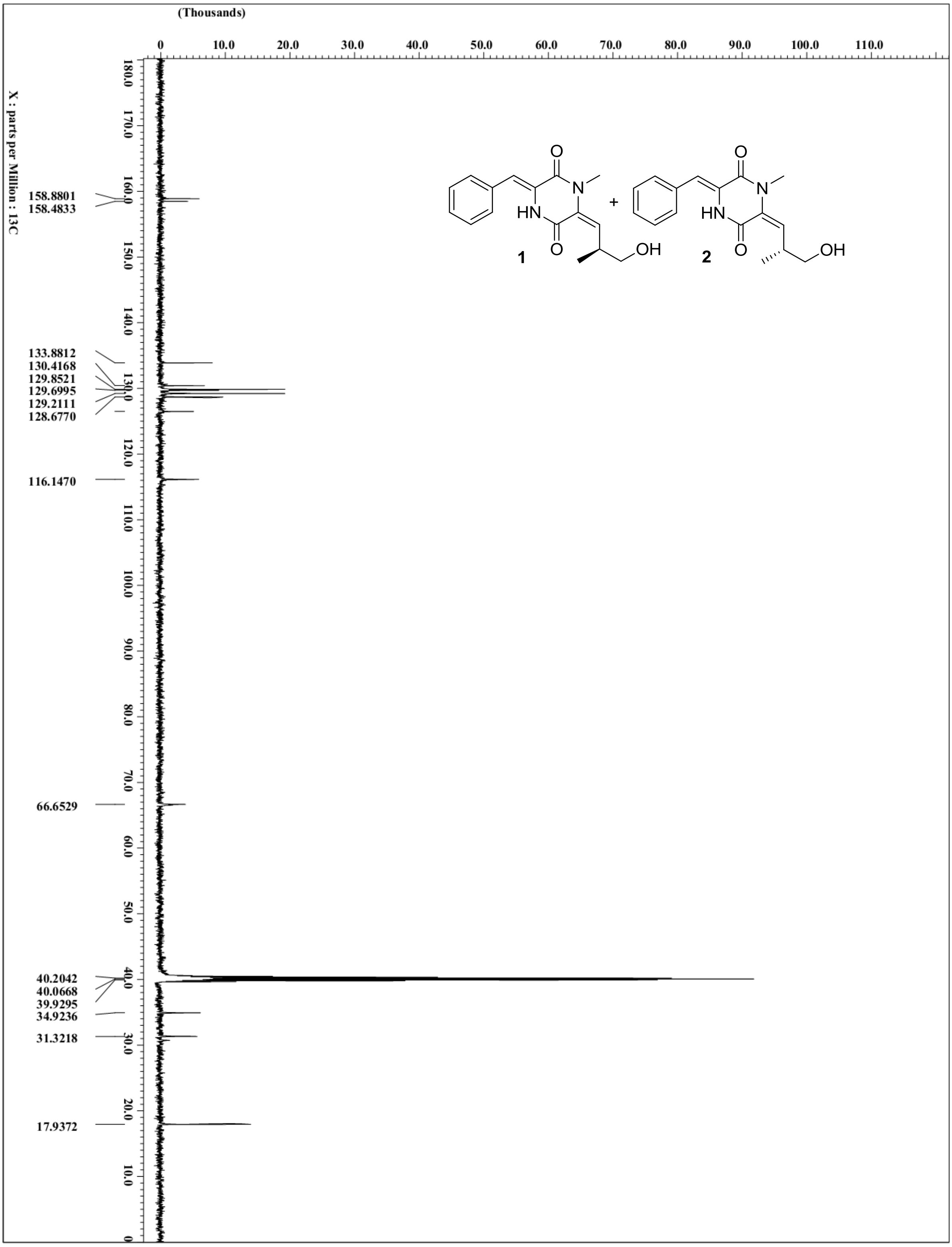

Figure S3. The DEPT (150 MHz, DMSO-d_6) spectrum of compounds **1** and **2**.

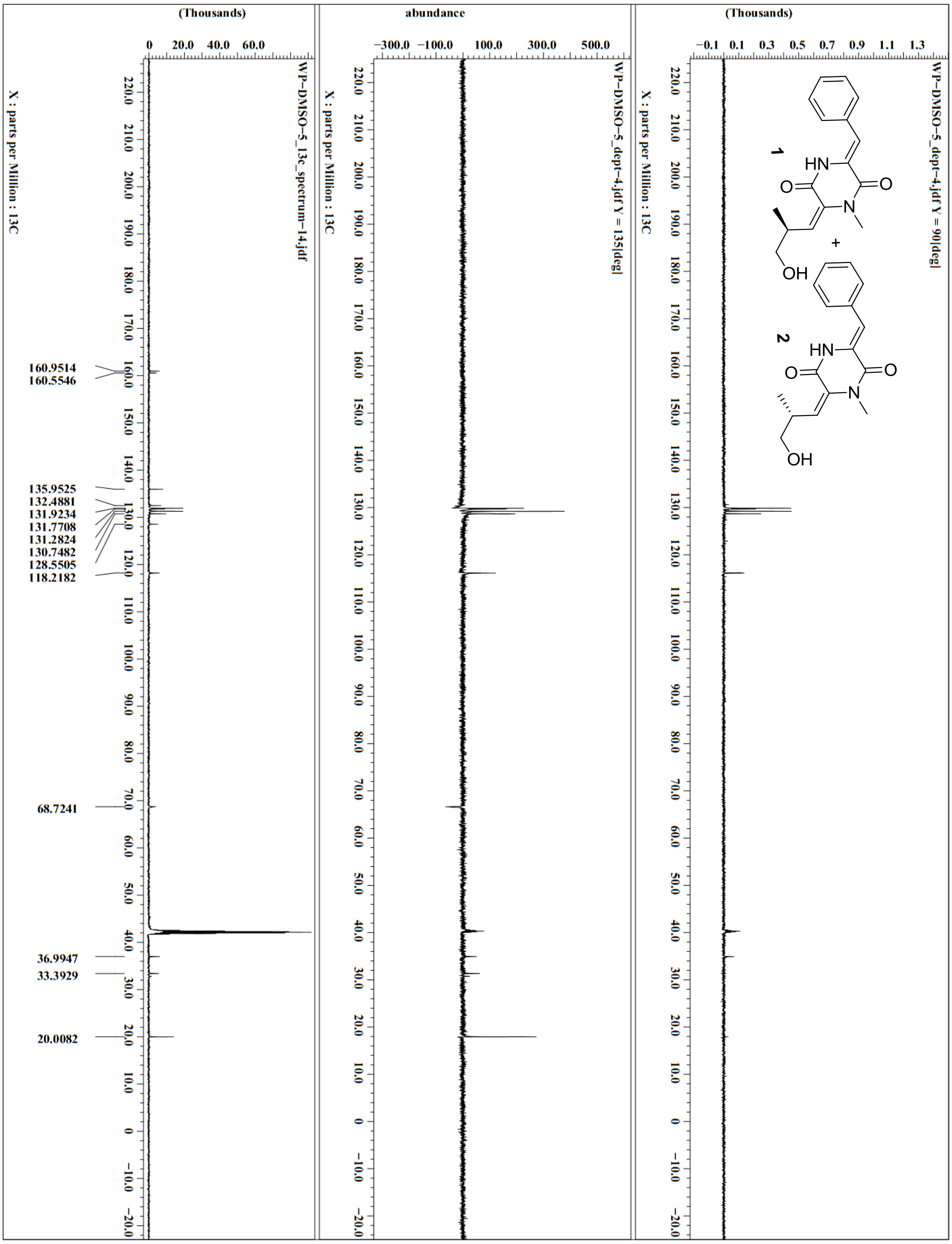

Figure S4. The ^{1}H-^{1}H COSY (600 MHz, DMSO-d_6) spectrum of compounds **1** and **2**.

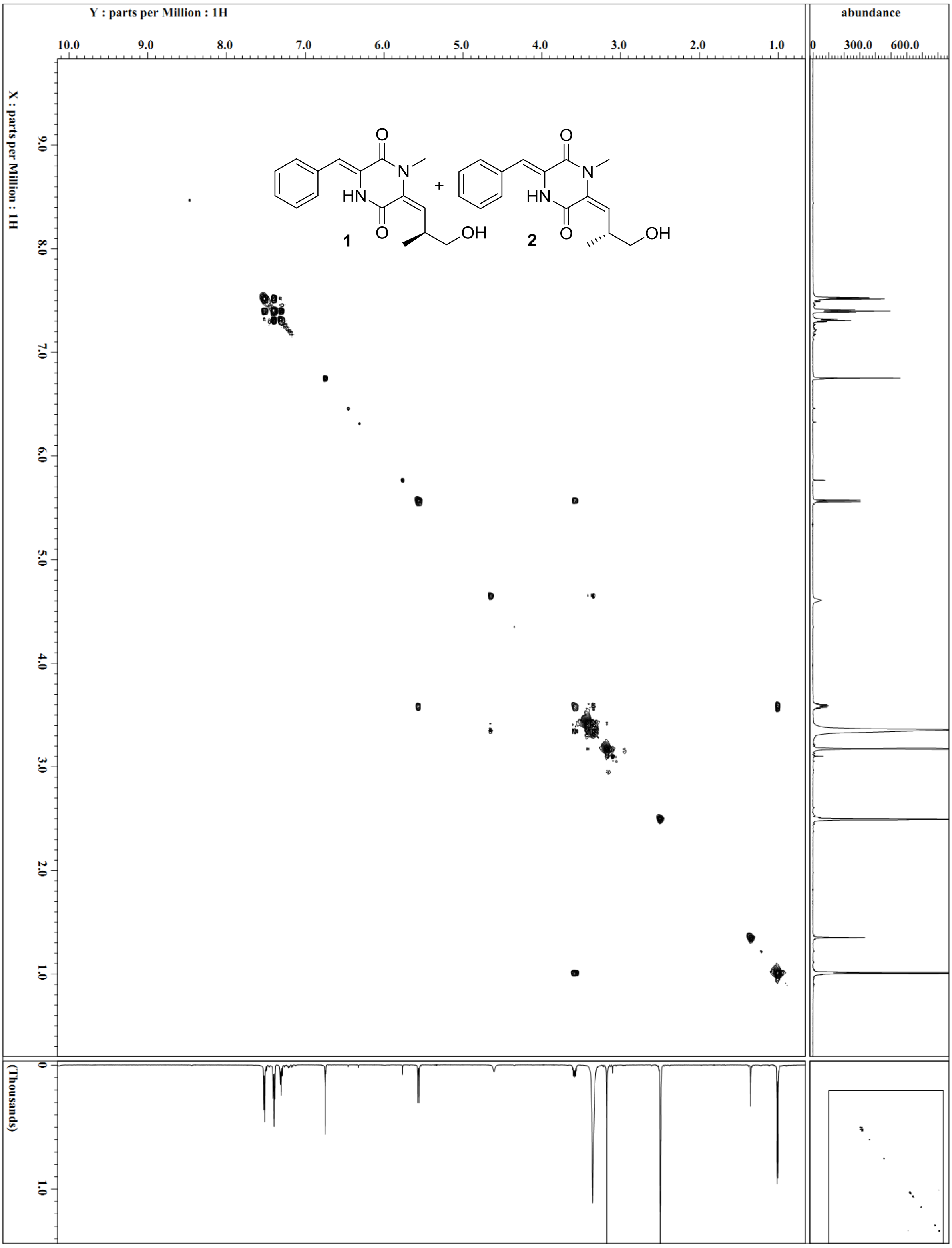

Figure S5. The HMQC (600 MHz, DMSO-d_6) spectrum of compounds **1** and **2**.

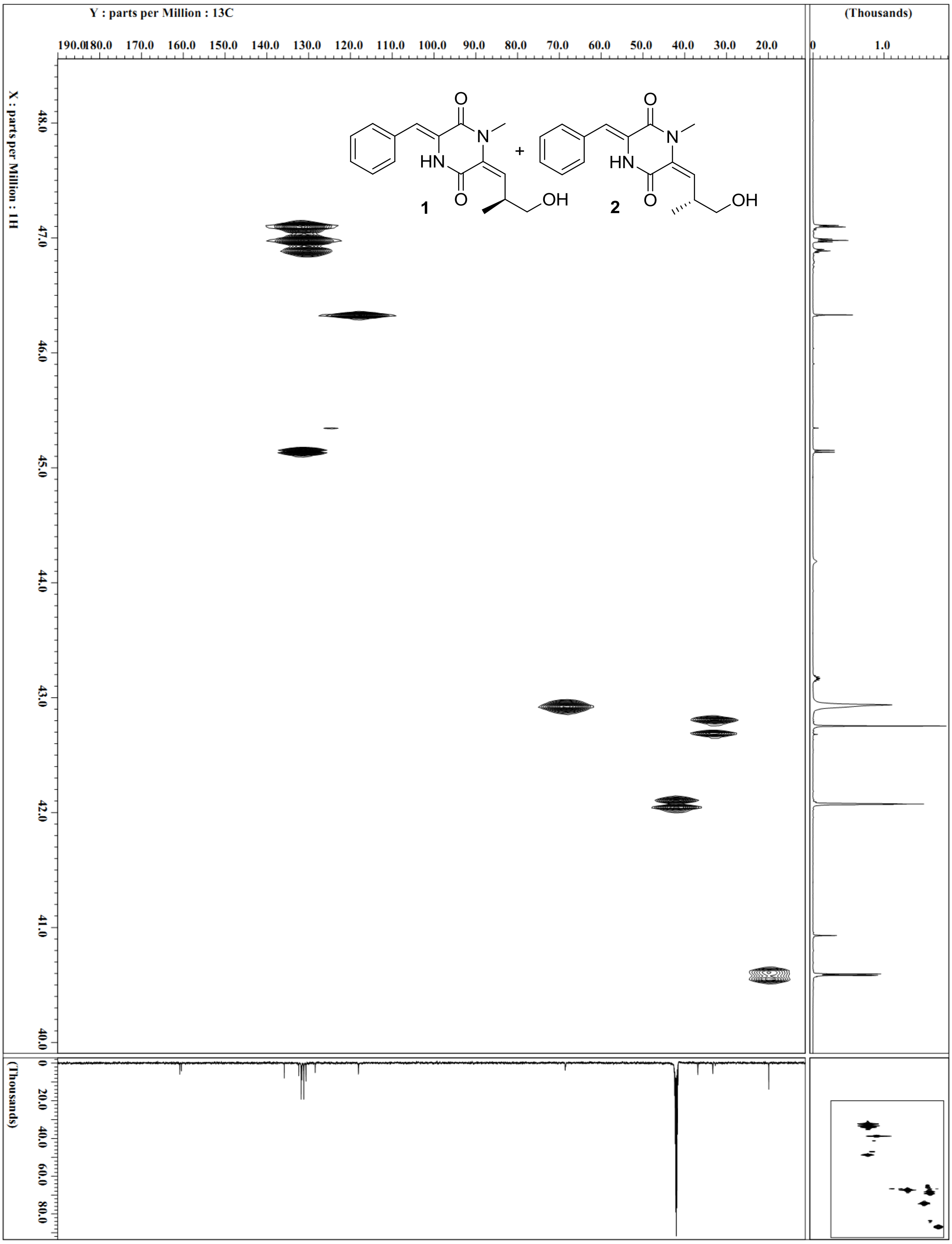

Figure S6. The HMBC (600 MHz, DMSO-d_6) spectrum of compounds **1** and **2**.

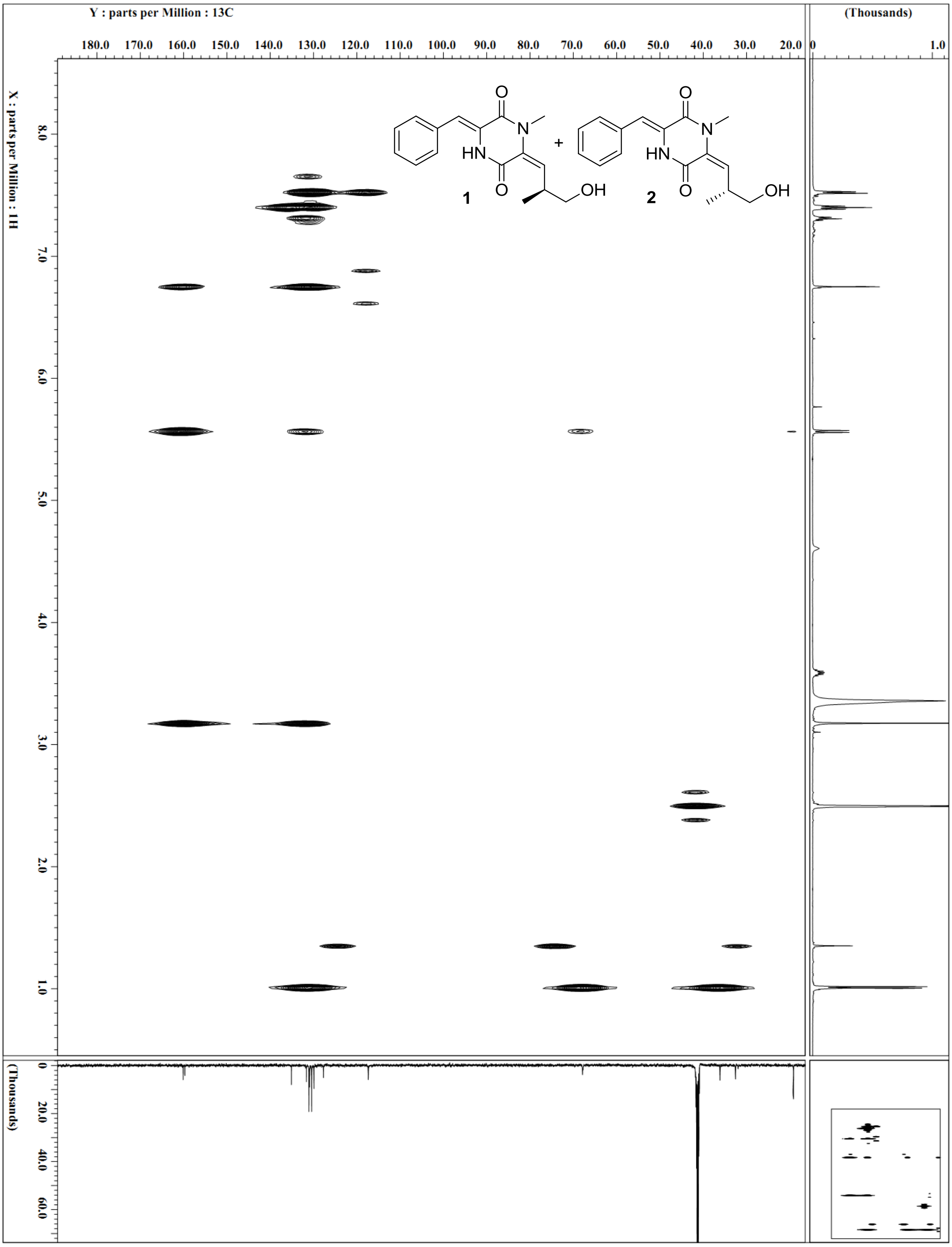

Figure S7. The NOESY (600 MHz, DMSO-d_6) spectrum of compounds **1** and **2**.

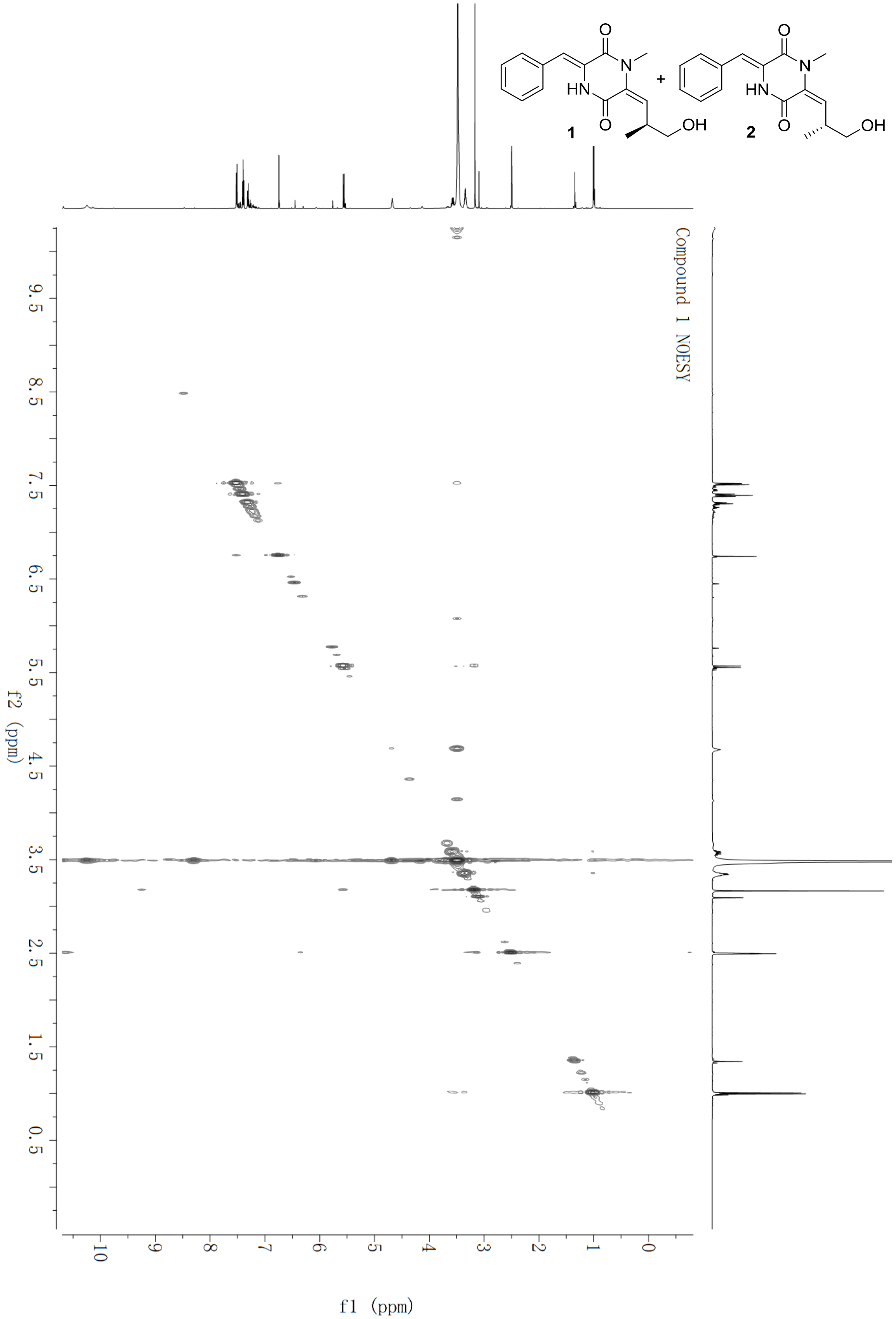

Figure S8. The ^{1}H NMR (600 MHz, DMSO-d_6) spectrum of compound **3**.

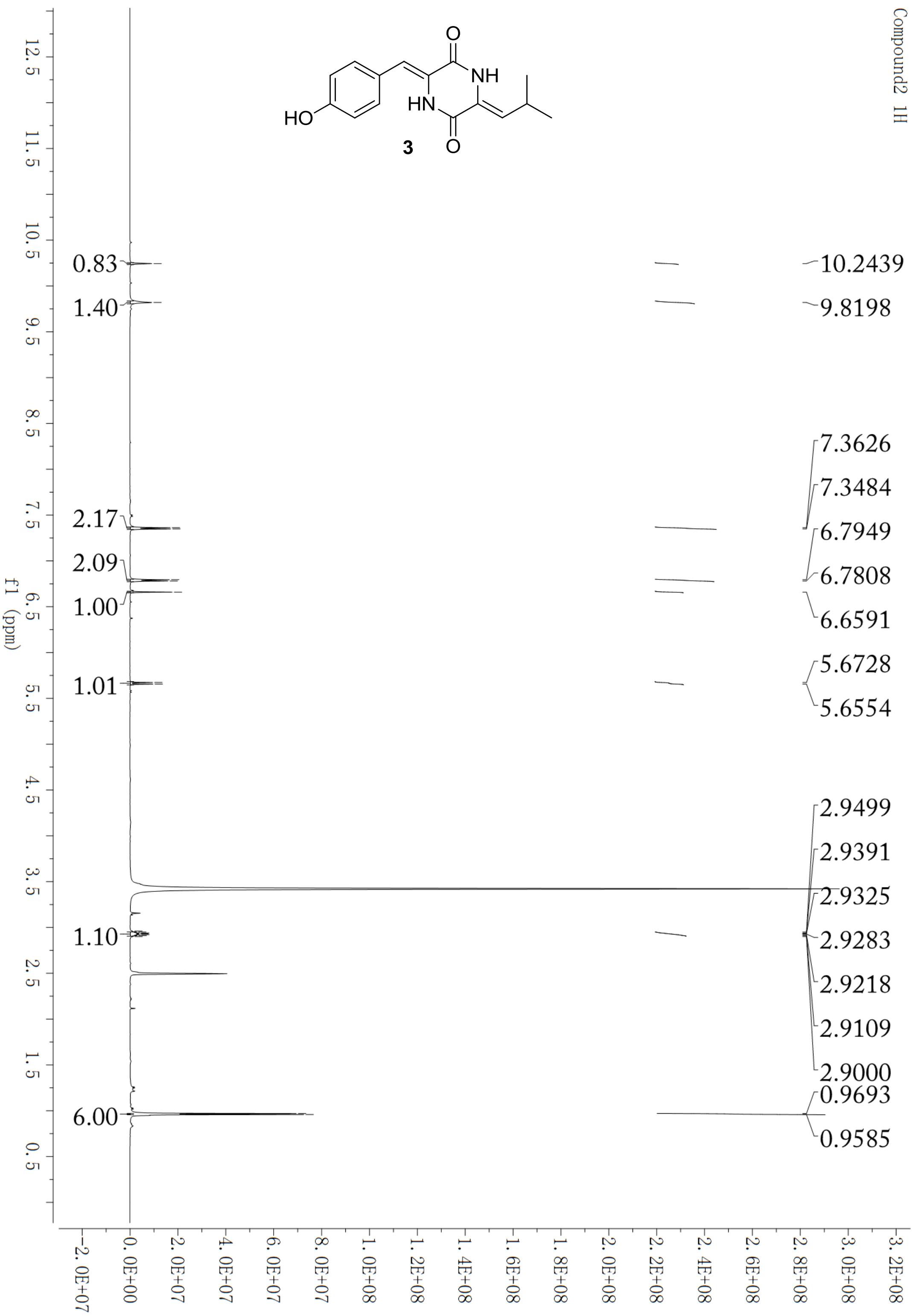

Figure S9. The ^{13}C NMR (150 MHz, DMSO-d_6) spectrum of compound **3**.

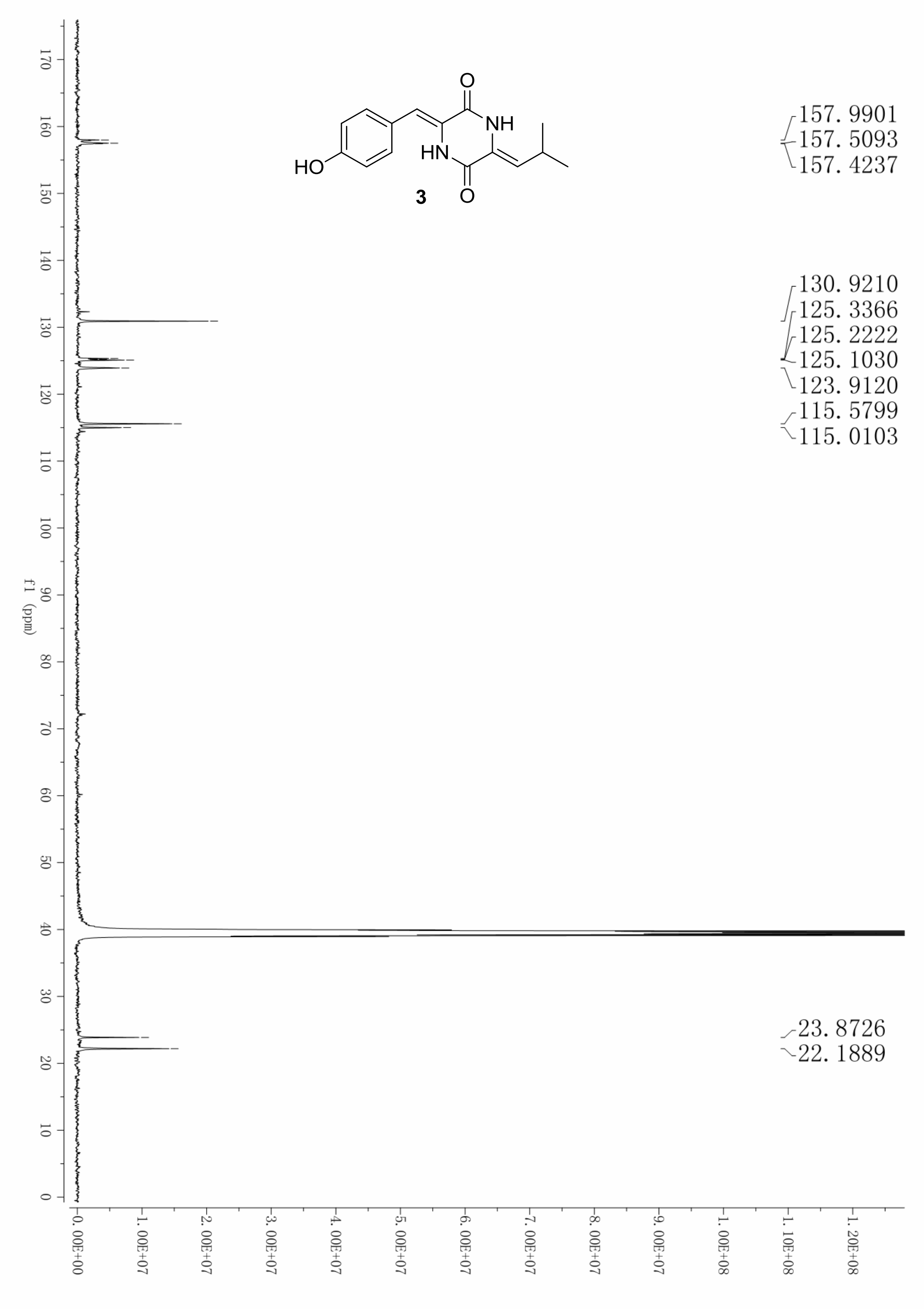

Figure S10. The DEPT (150 MHz, DMSO-d_6) spectrum of compound **3**.

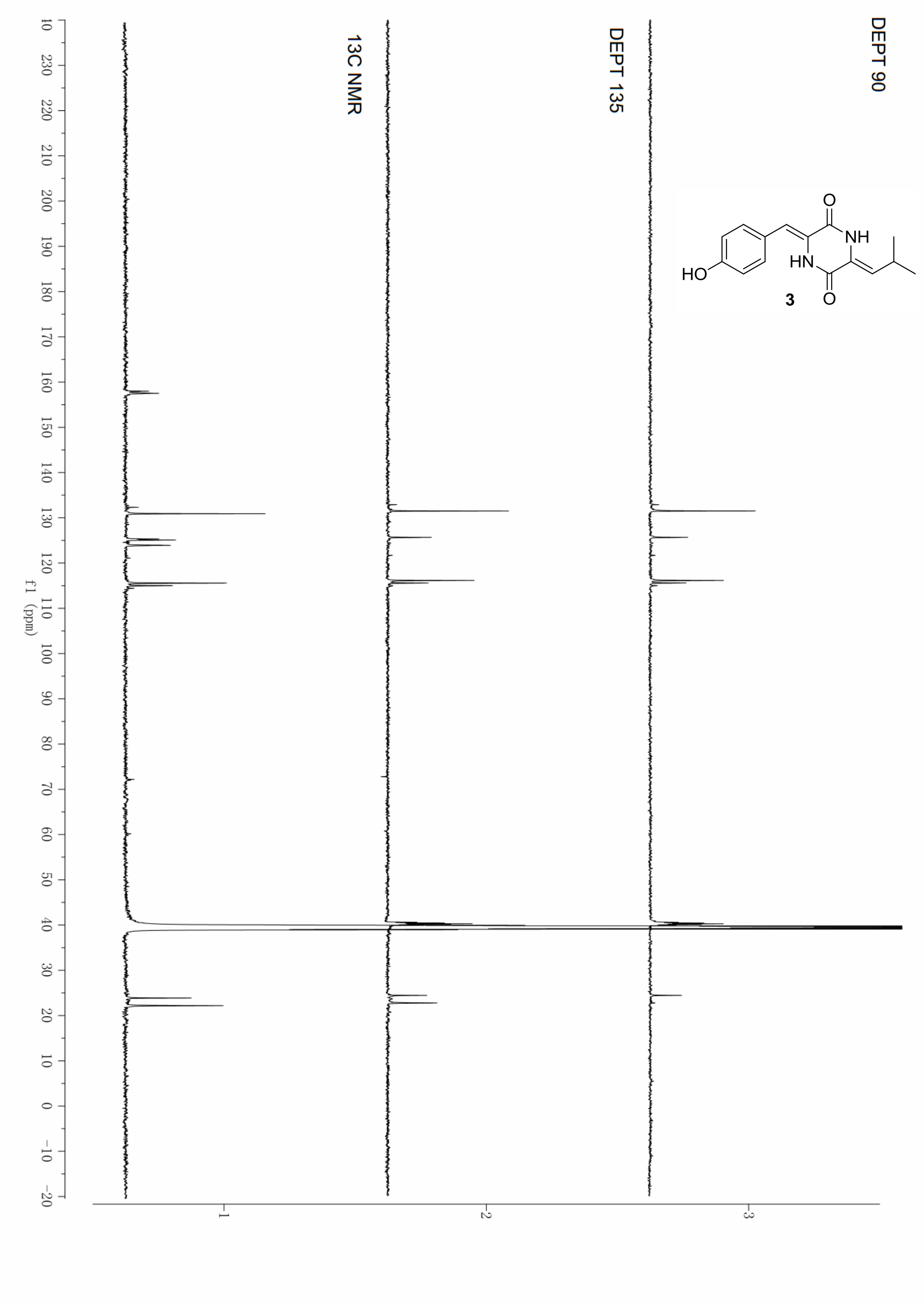

Figure S11. The HMQC (6000 MHz, DMSO-d_6) spectrum of compound **3**.

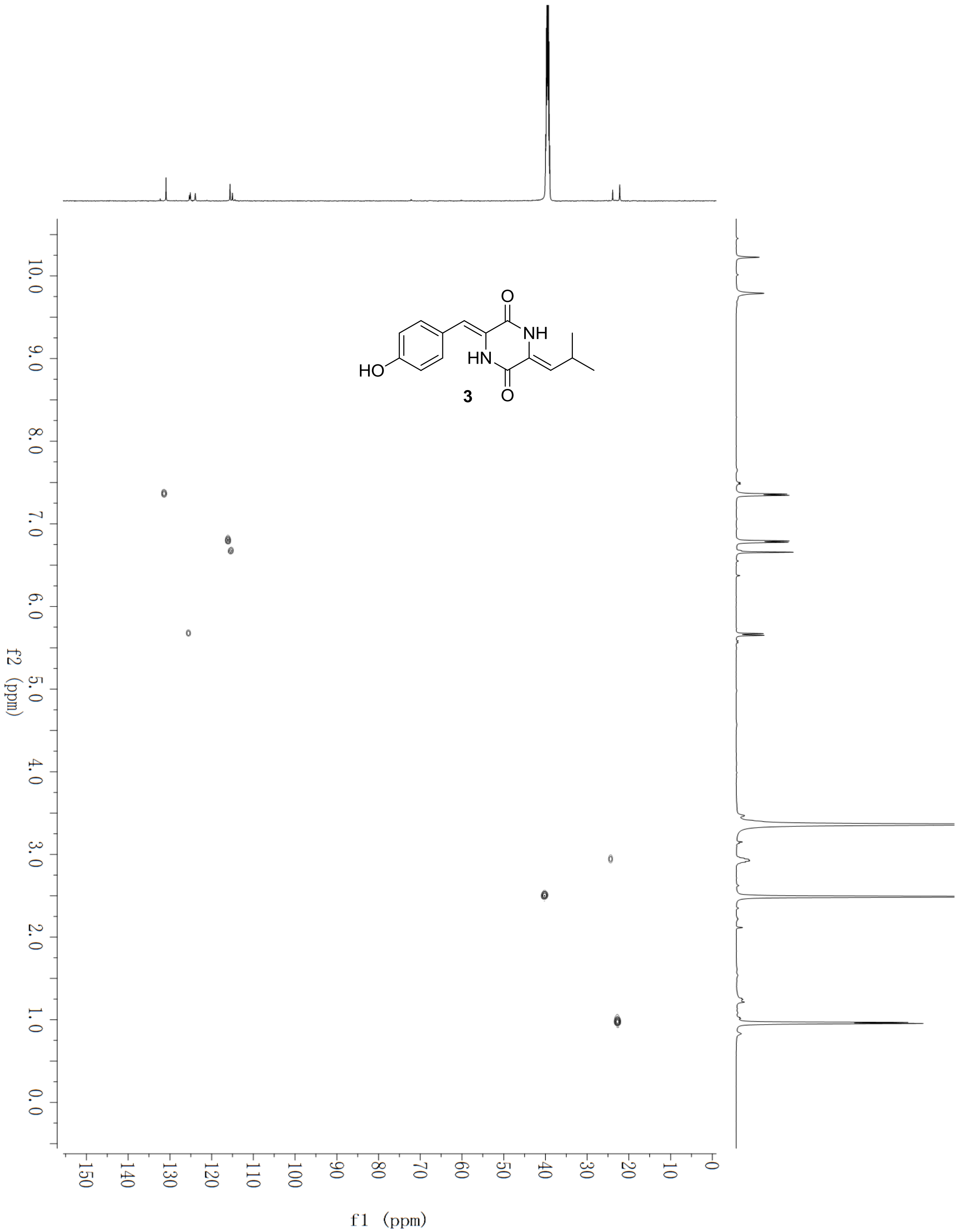

Figure S12. The ^{1}H-^{1}H COSY (600 MHz, DMSO-d_6) spectrum of compound **3**.

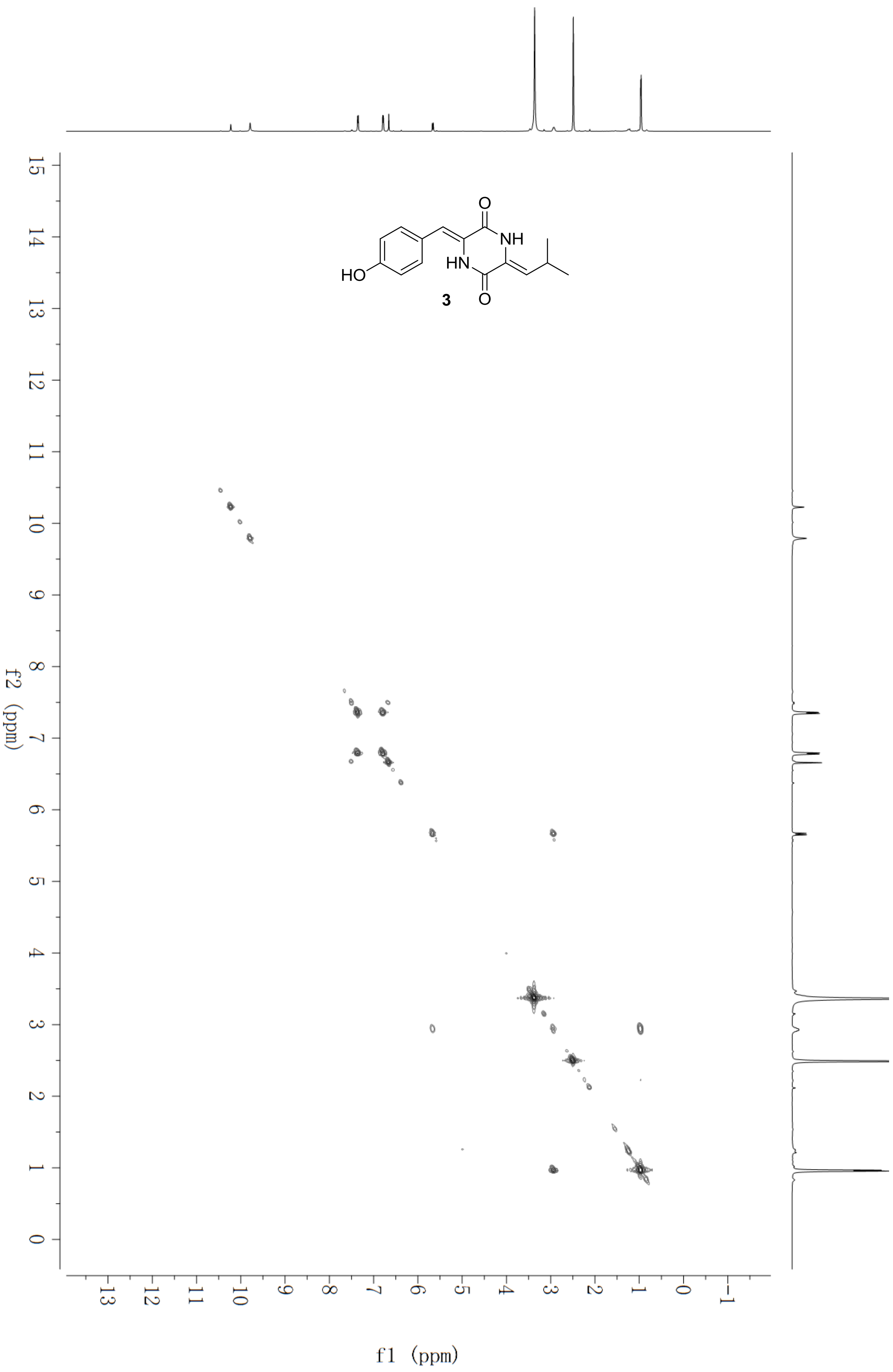

Figure S13. The HMBC (600 MHz, DMSO-d_6) spectrum of compound **3**.

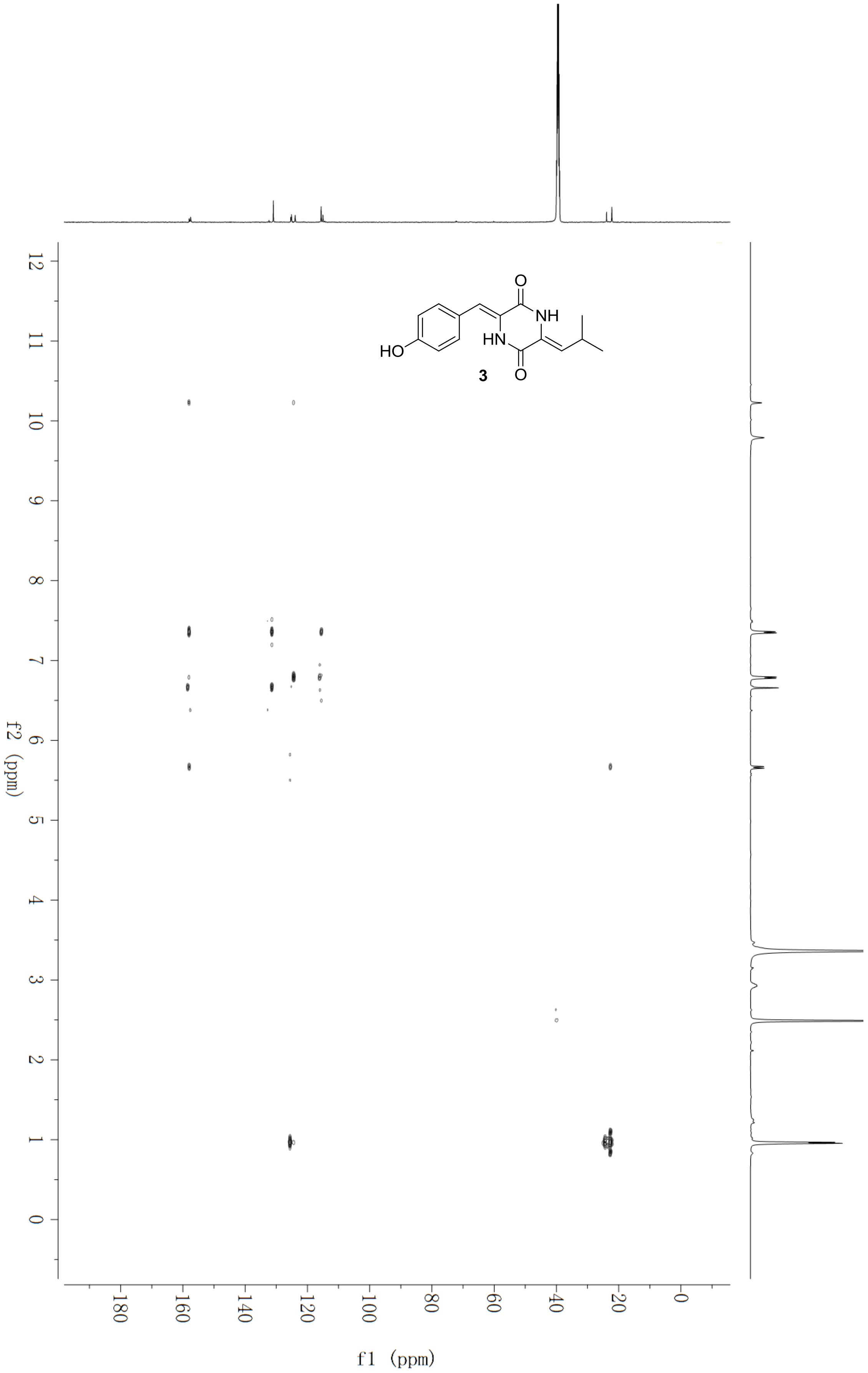

Figure S14. The NOESY (600 MHz, DMSO-d_6) spectrum of compound **3**.

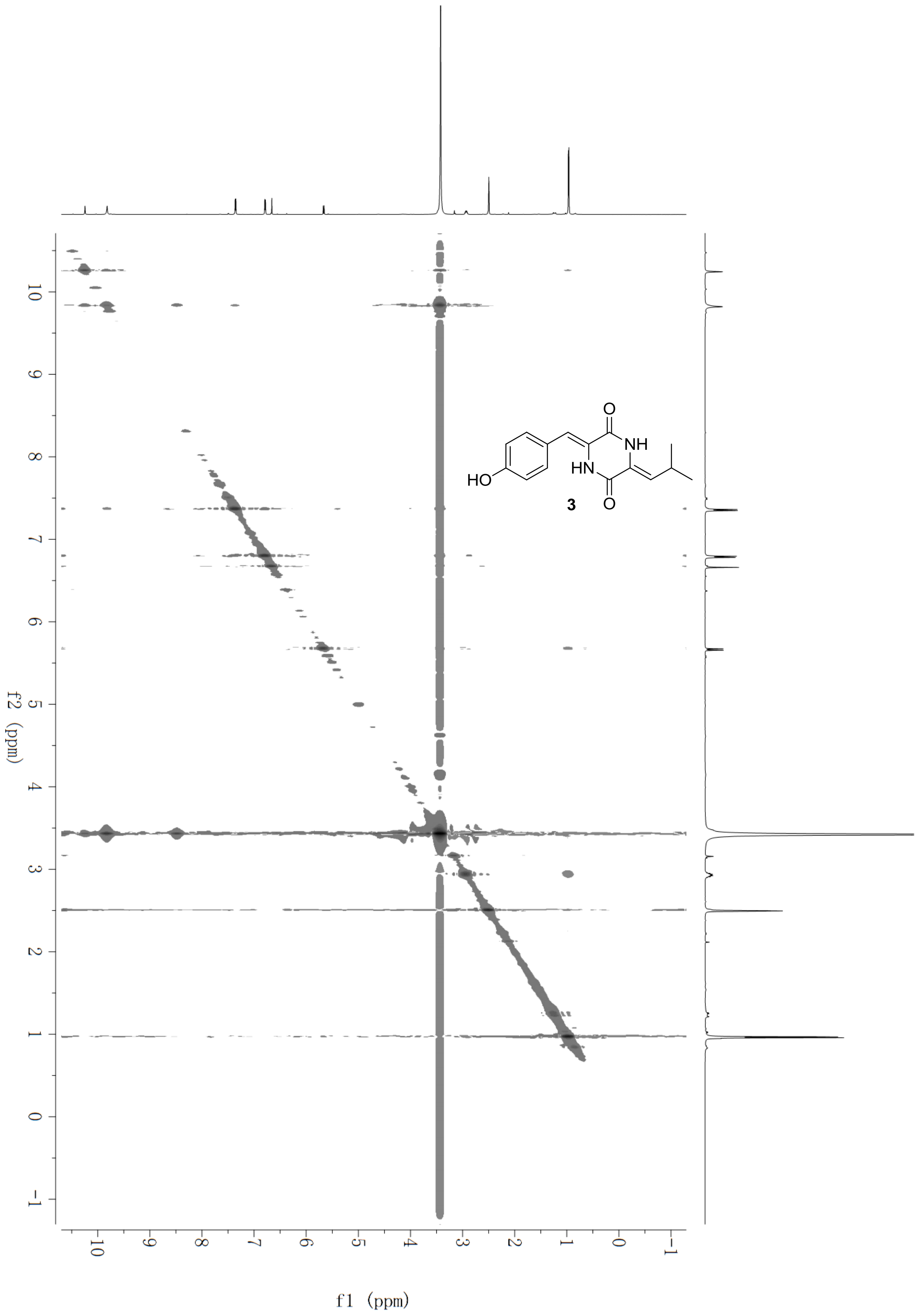

Figure S15. The ^{1}H NMR (600 MHz, DMSO-d_6) spectrum of compound **4**.

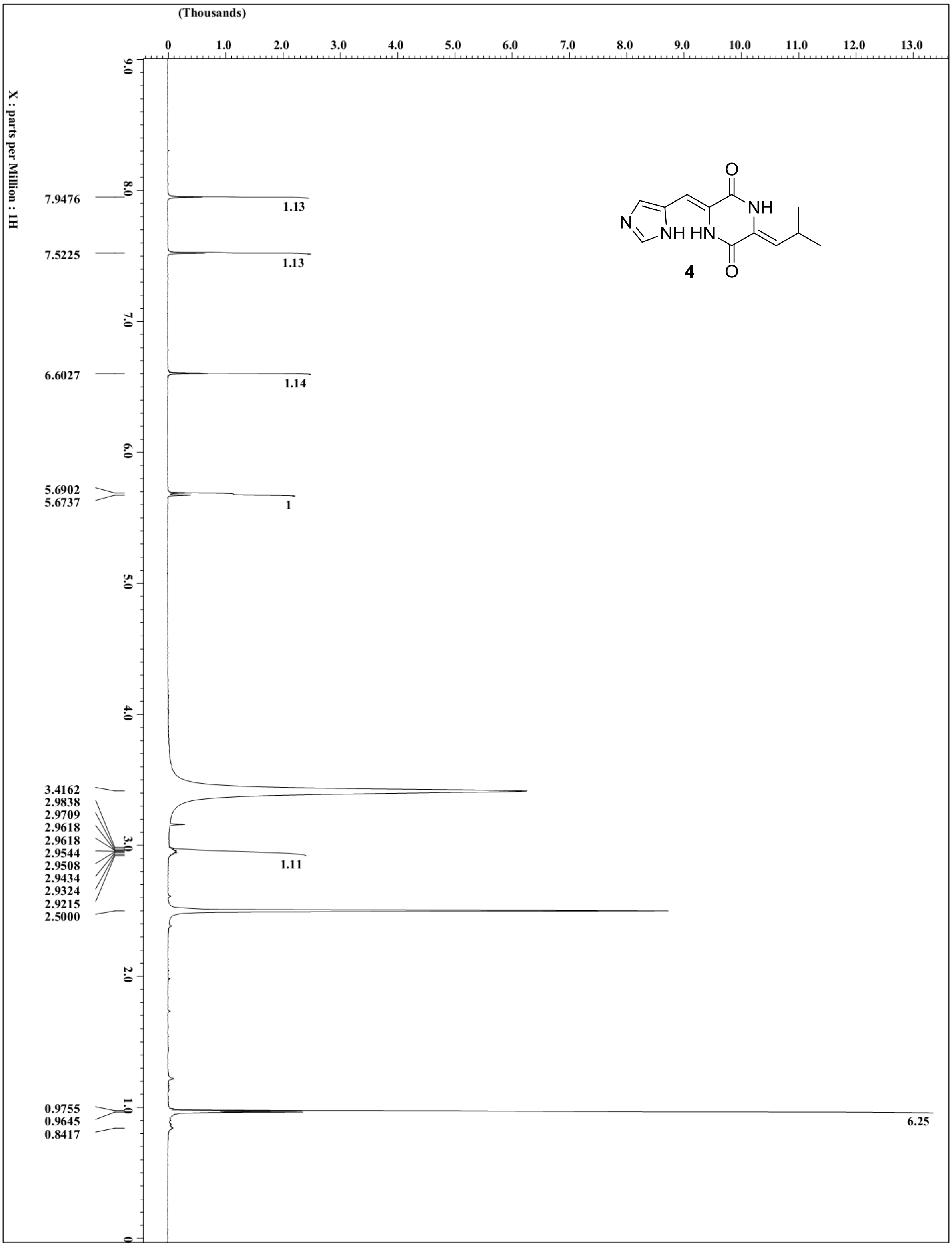

Figure S16. The ^{13}C NMR (150 MHz, DMSO-d_6) spectrum of compound **4**.

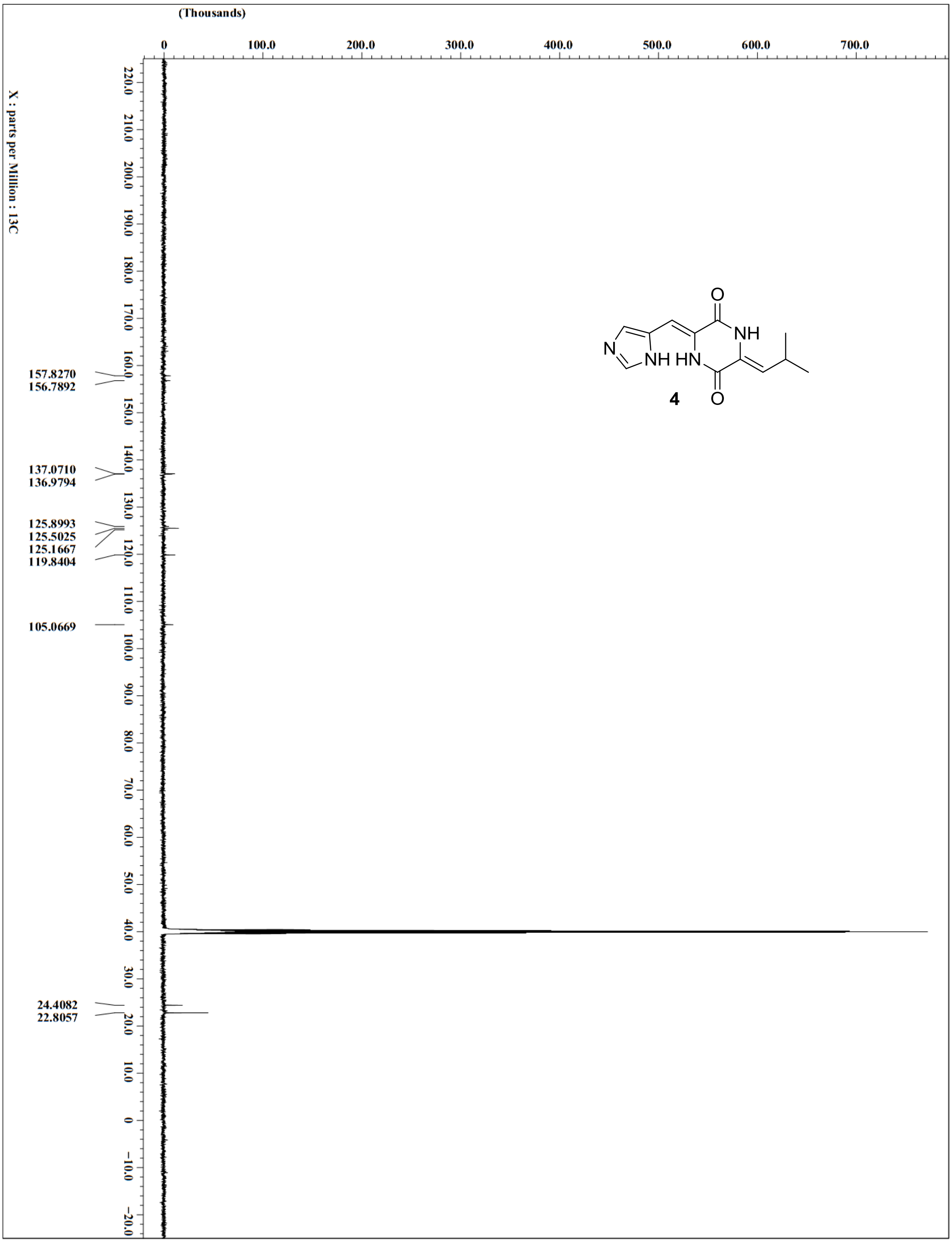

Figure S17. The DEPT (150 MHz, DMSO-d_6) spectrum of compound **4**.

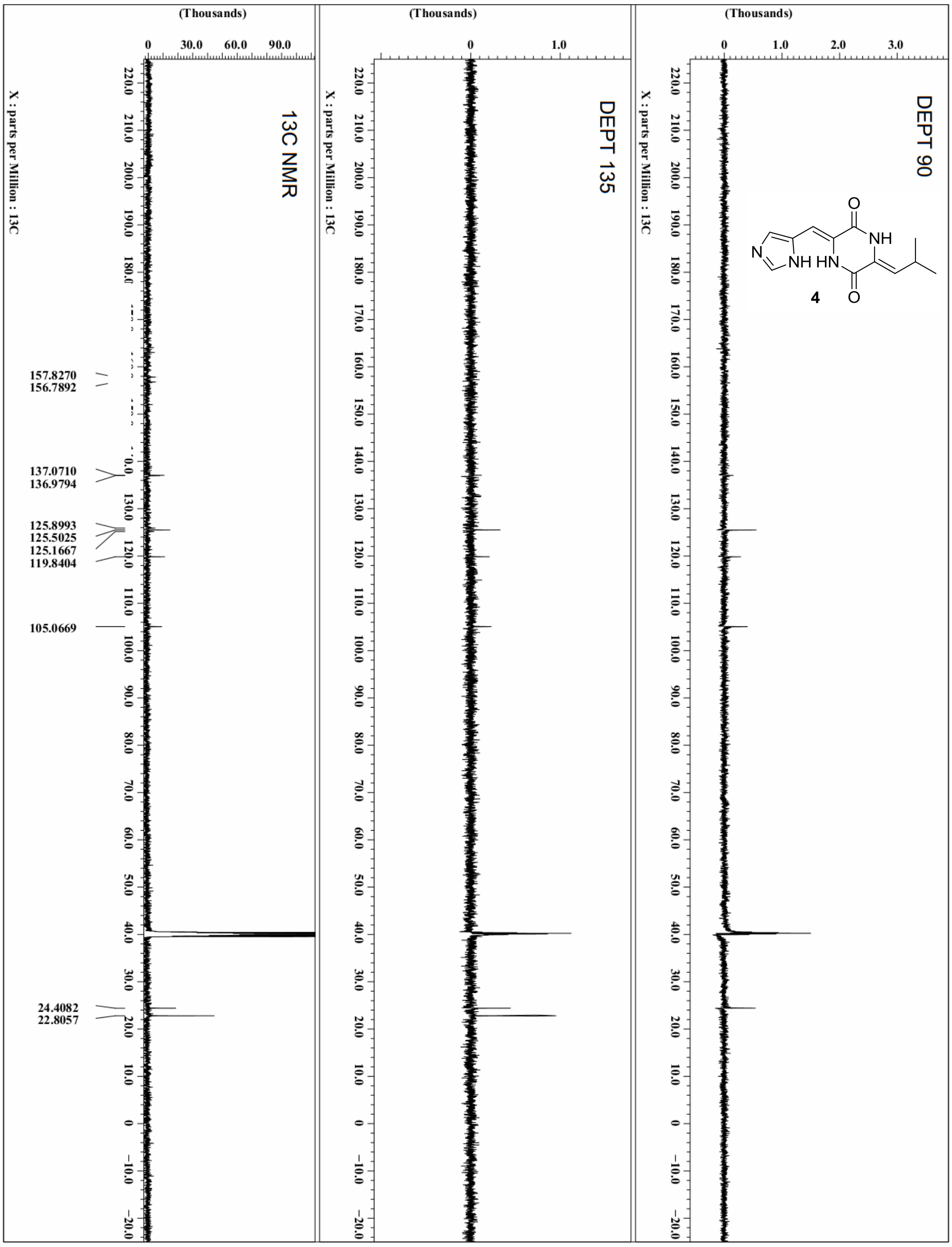

Figure S18. The ^{1}H-^{1}H COSY (600 MHz, DMSO-d_6) spectrum of compound **4**.

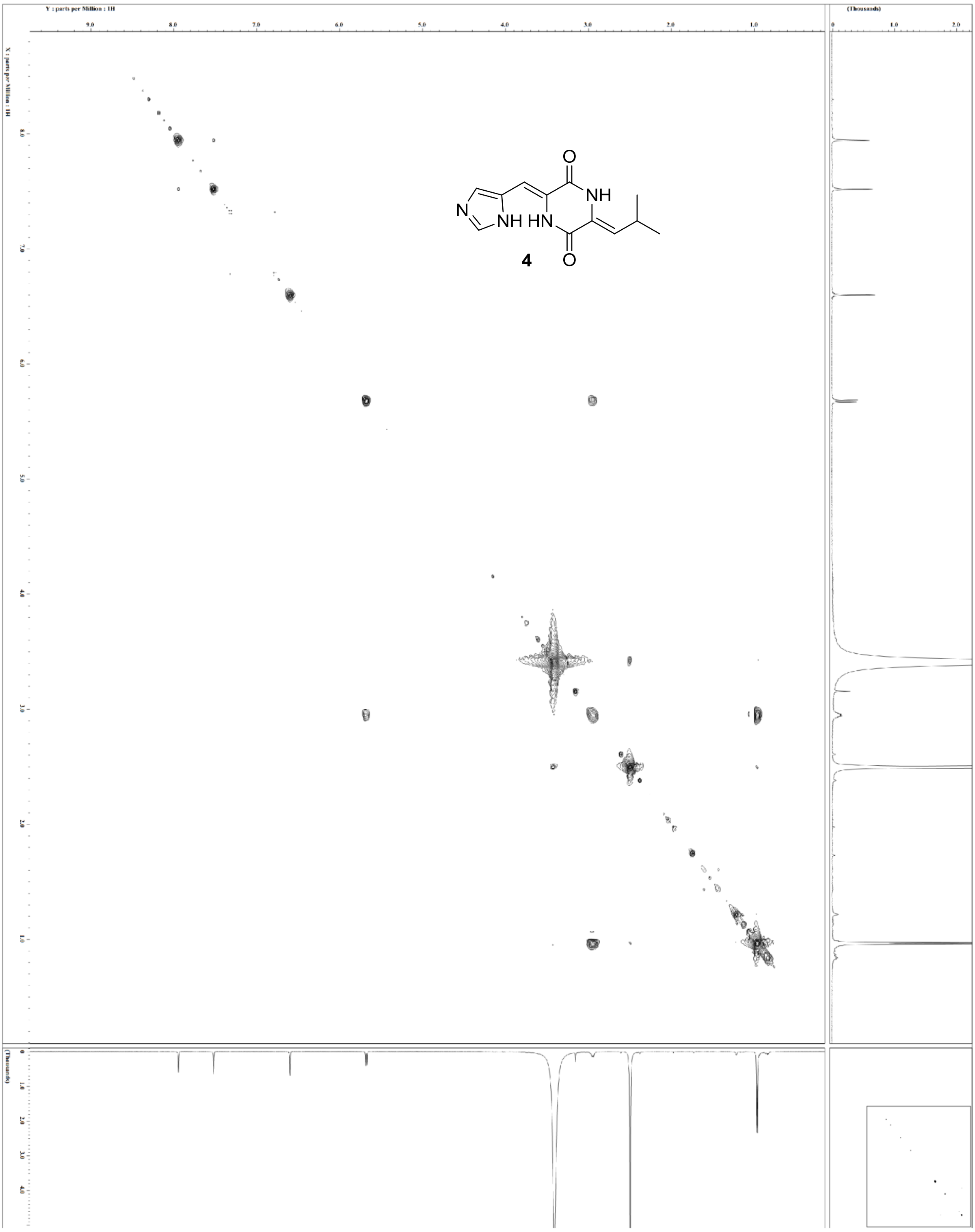

Figure S19. The HMQC (600 MHz, DMSO-d_6) spectrum of compound **4**.

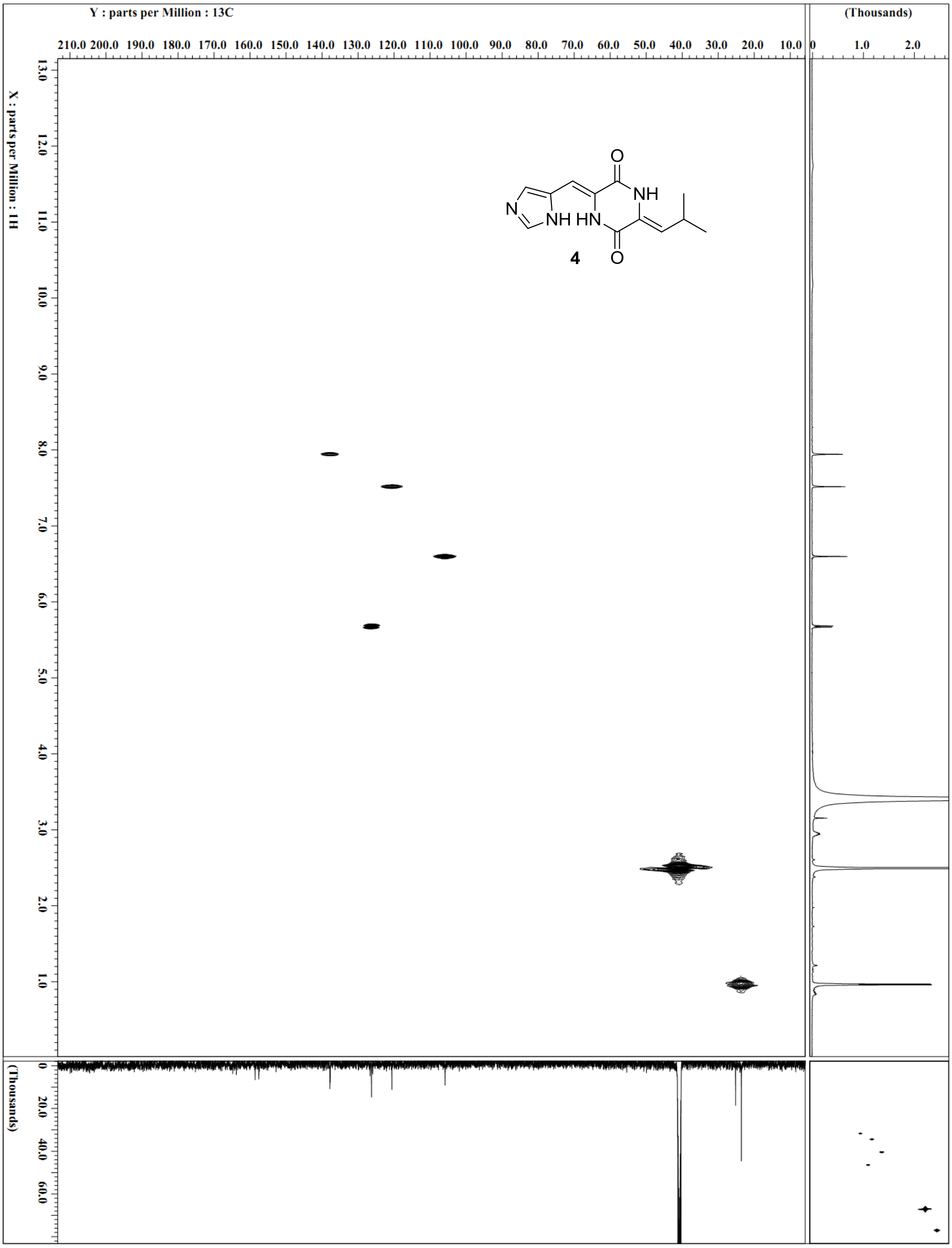

Figure S20. The HMBC (600 MHz, DMSO-d_6) spectrum of compound **4**.

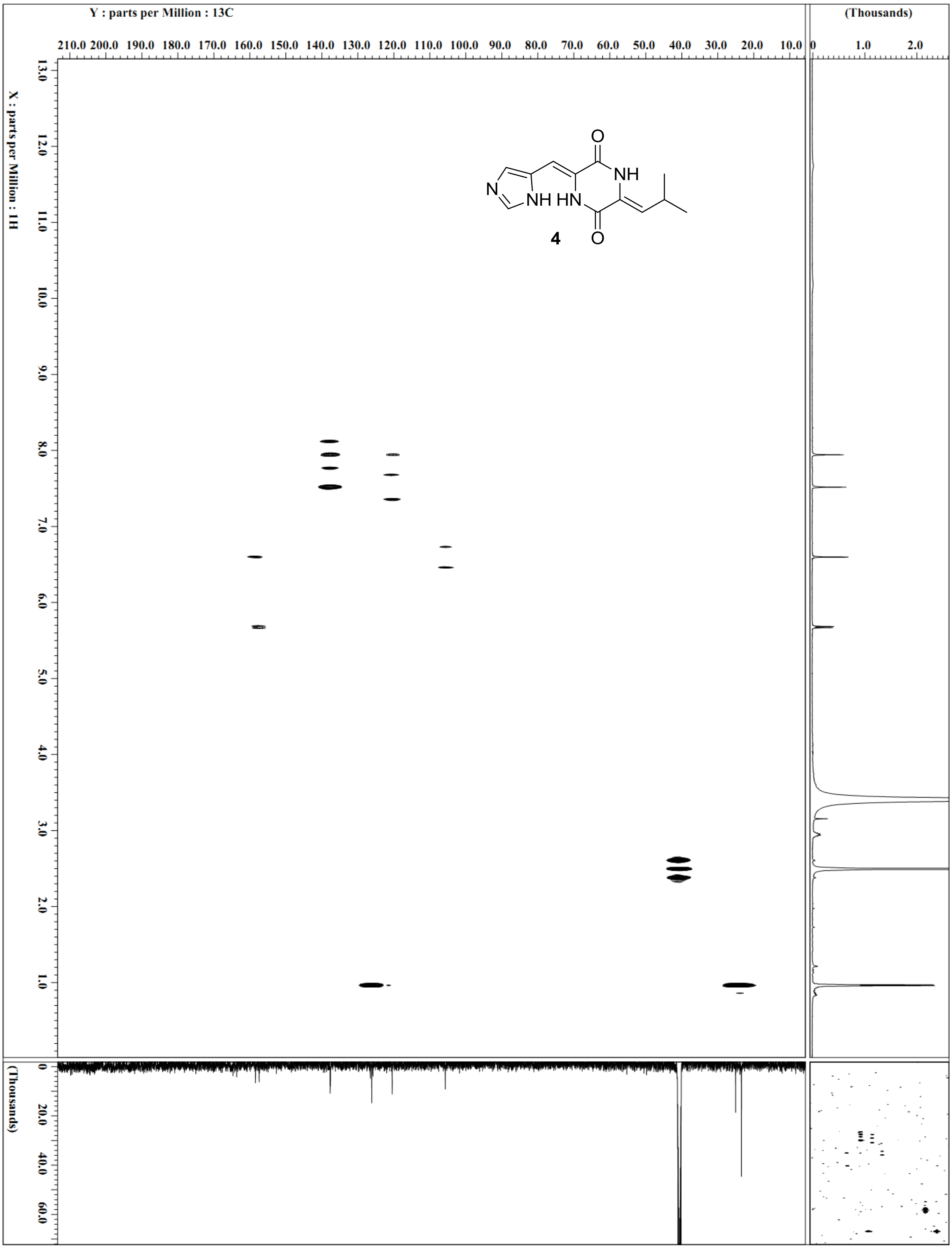

Figure S21. The ^{1}H NMR (600 MHz, DMSO-d_6) spectrum of compound **5**.

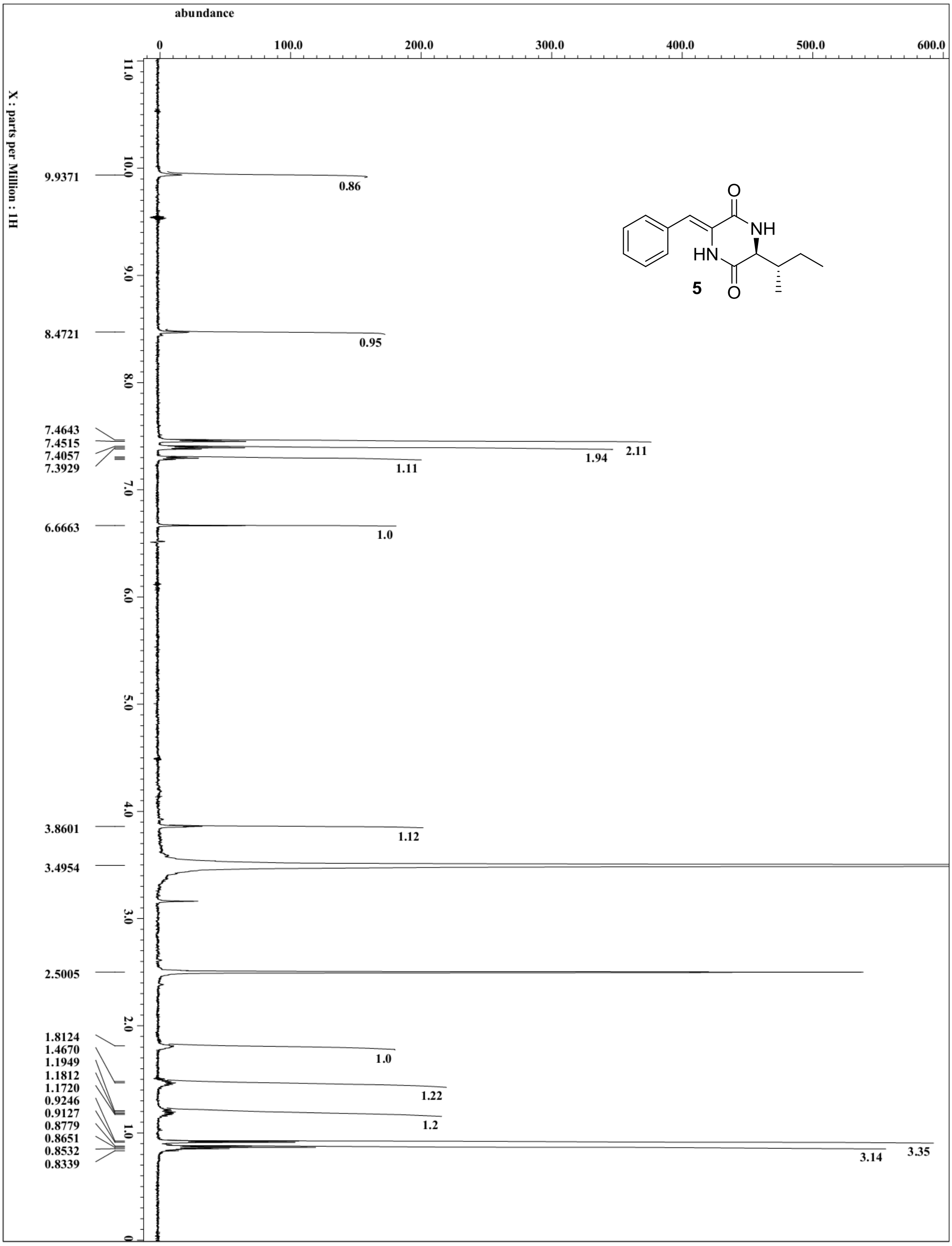

Figure S22. The ^{13}C NMR (150 MHz, DMSO-d_6) spectrum of compound **5**.

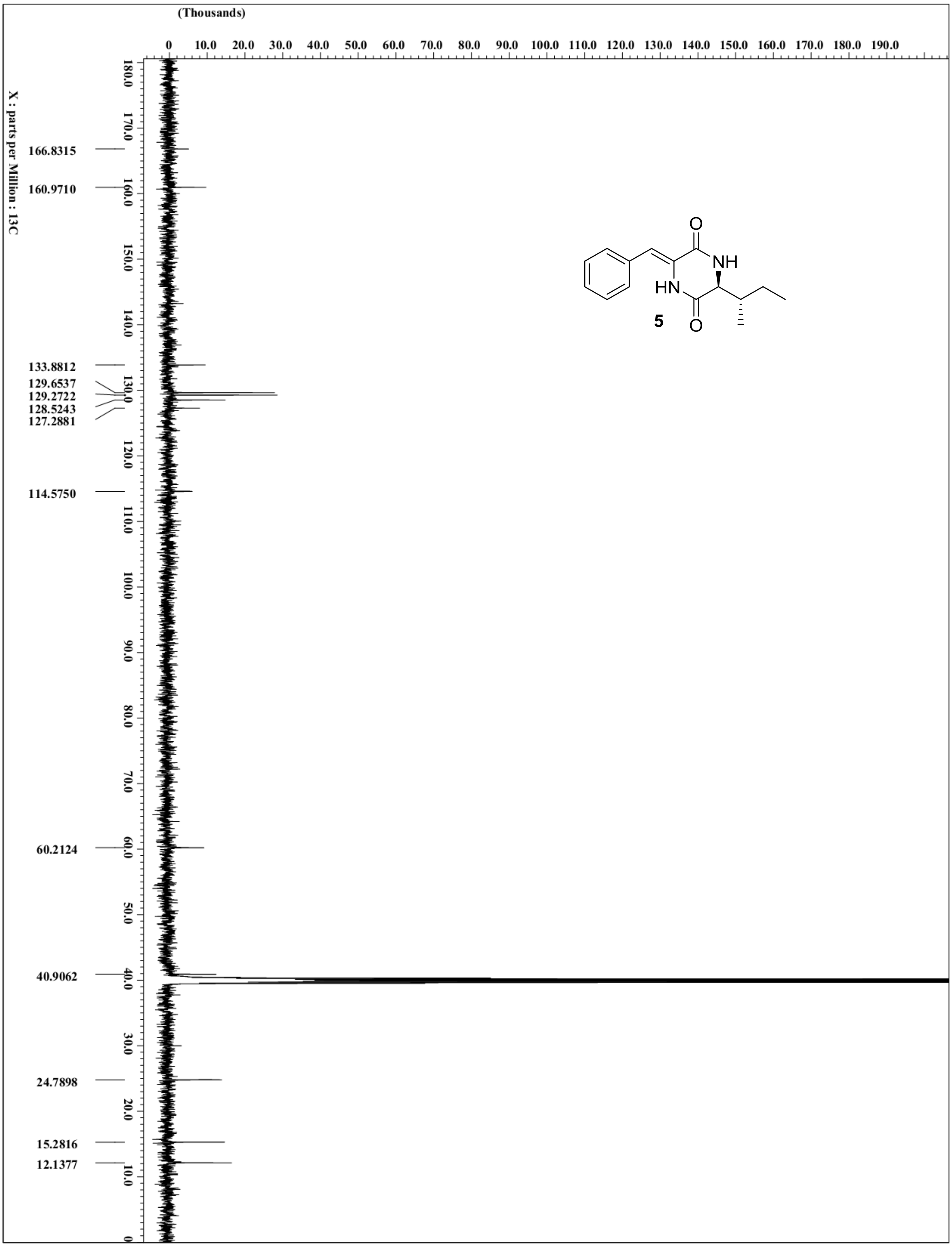

Figure S23. The DEPT (150 MHz, DMSO-d_6) spectrum of compound **5**.

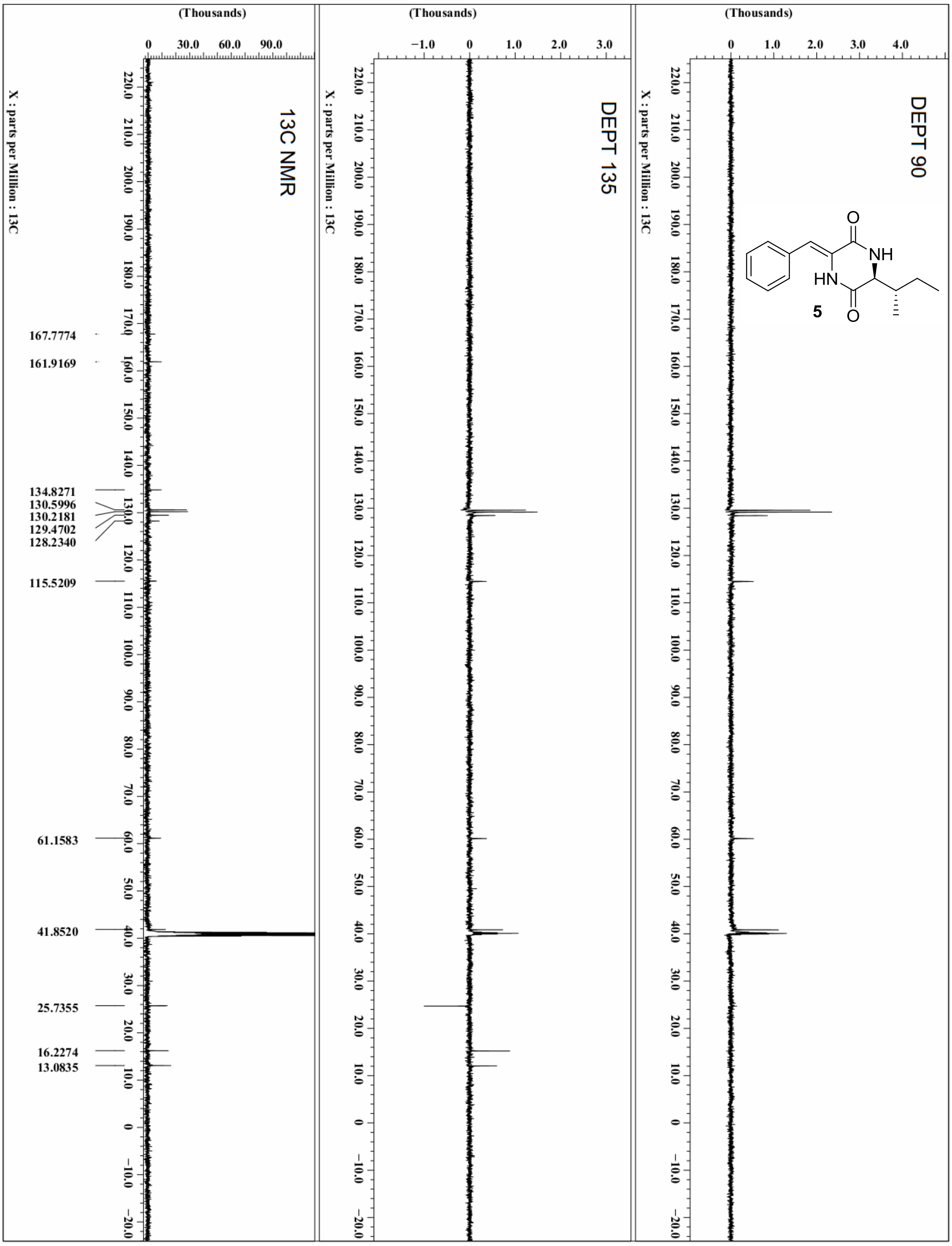

Figure S24. The ^{1}H-^{1}H COSY (600 MHz, DMSO-d_6) spectrum of compound **5**.

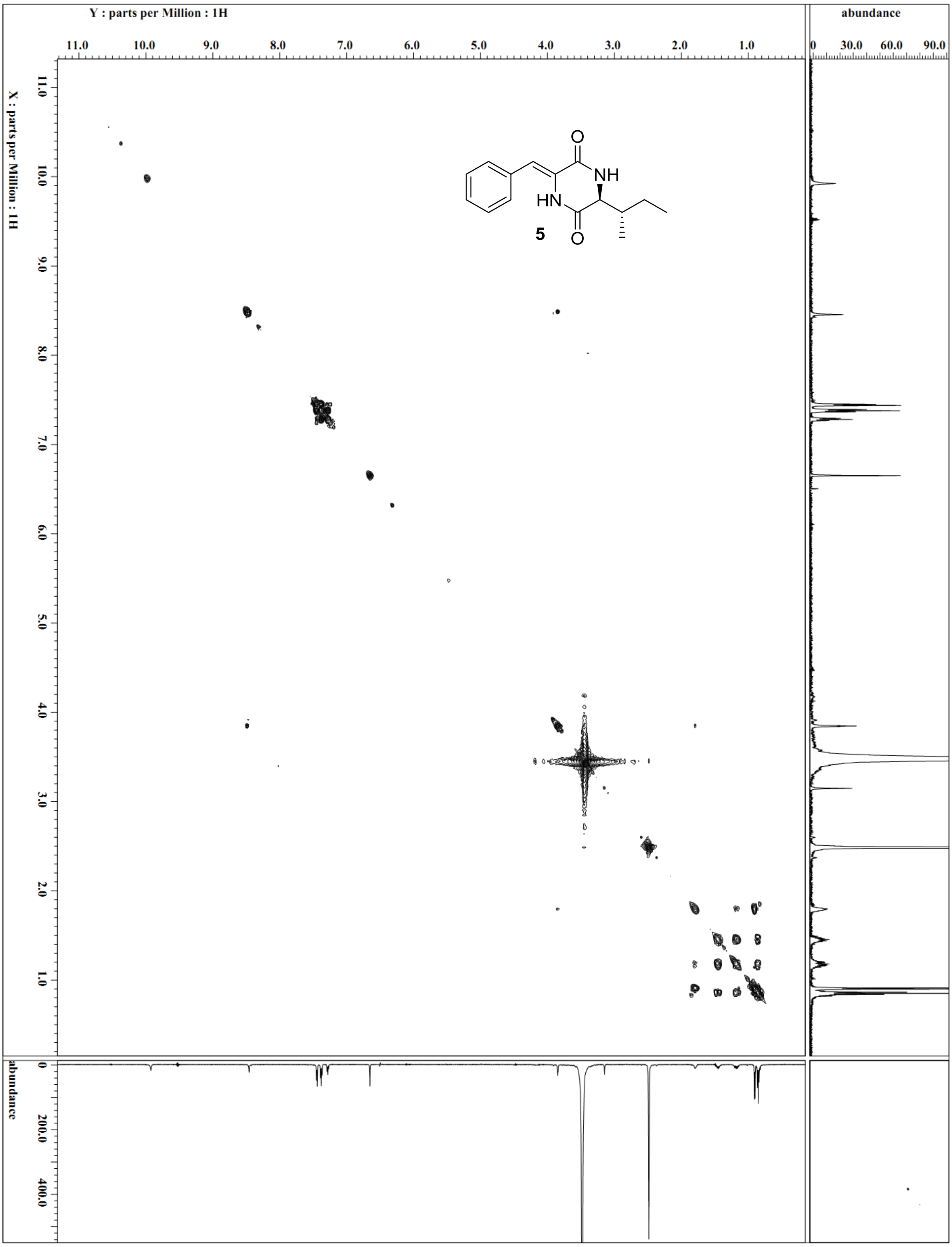

Figure S25. The HMQC (600 MHz, DMSO-d_6) spectrum of compound **5**.

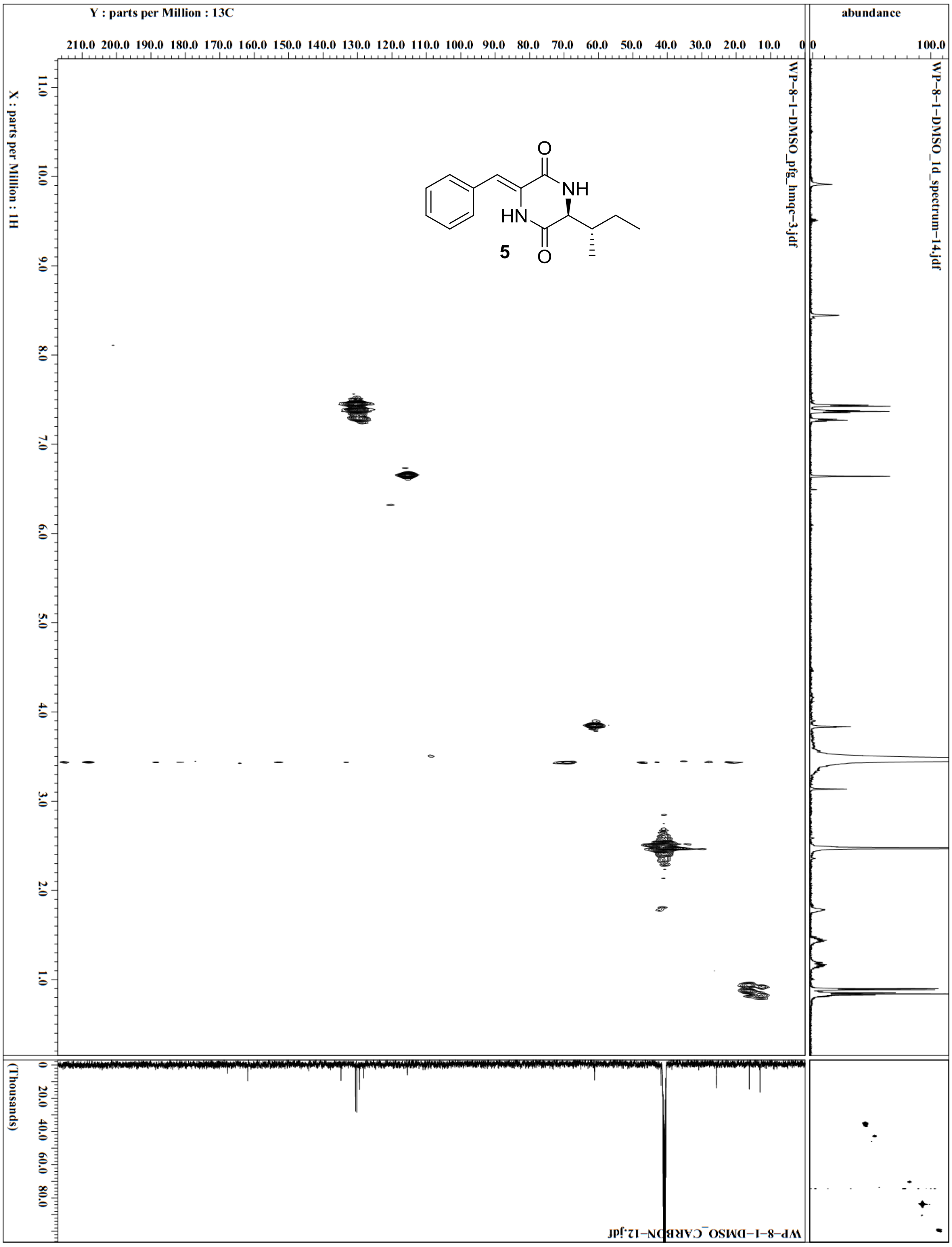

Figure S26. The HMBC (600 MHz, DMSO-d_6) spectrum of compound **5**.

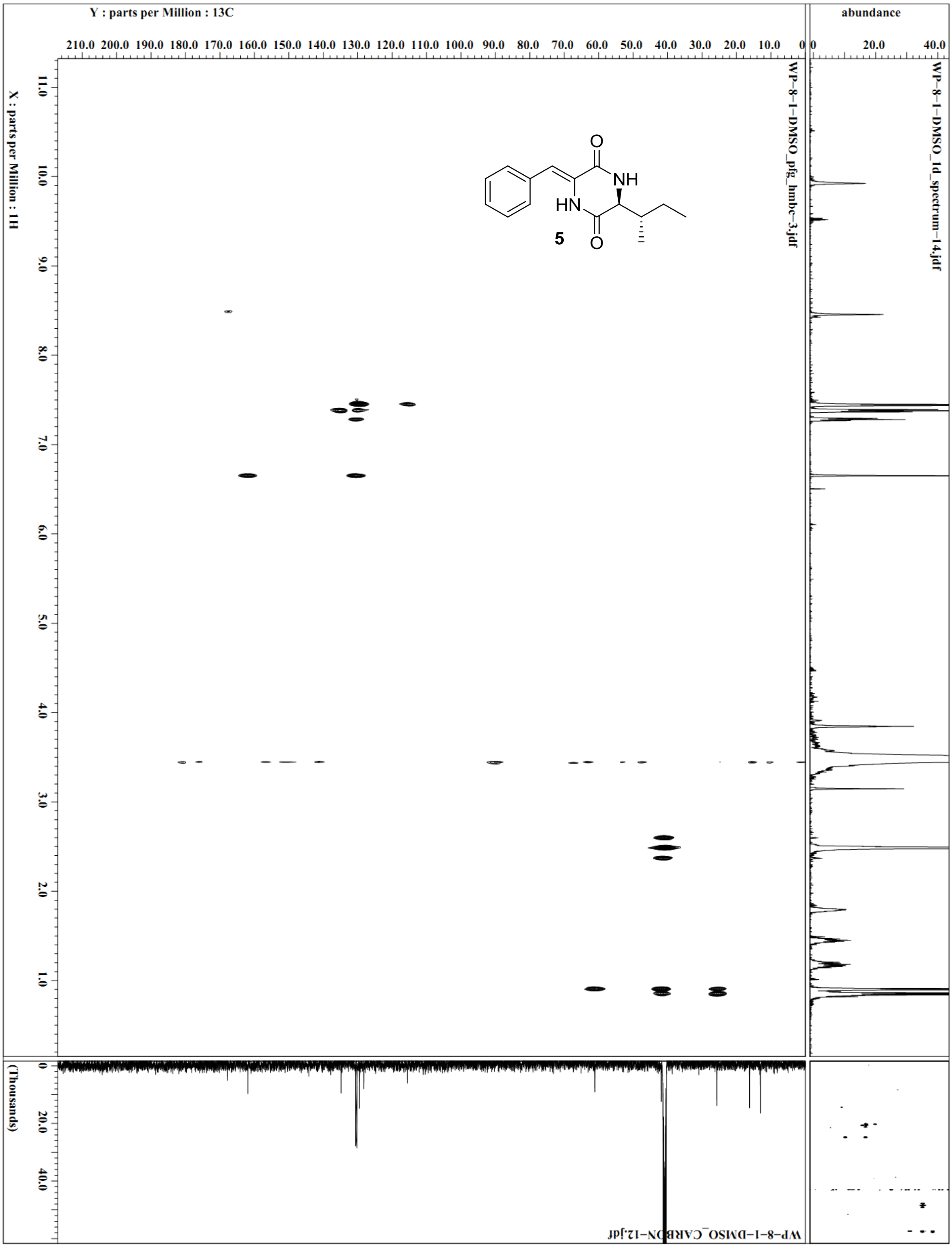

Figure S27. The NOESY (600 MHz, DMSO-d_6) spectrum of compound **5**.

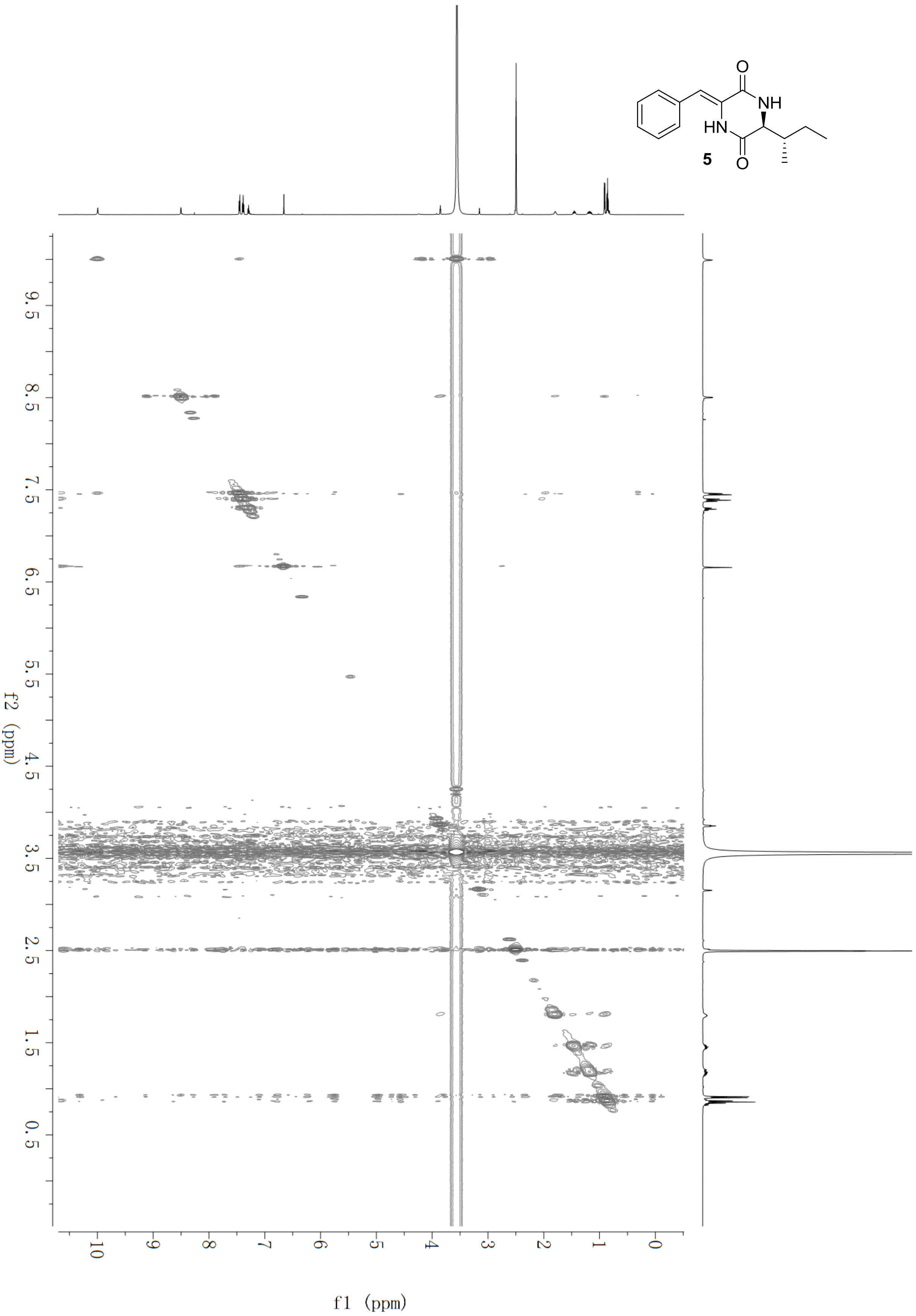

Figure S28. The ^{1}H NMR (600 MHz, DMSO-d_6) spectrum of compound **6**.

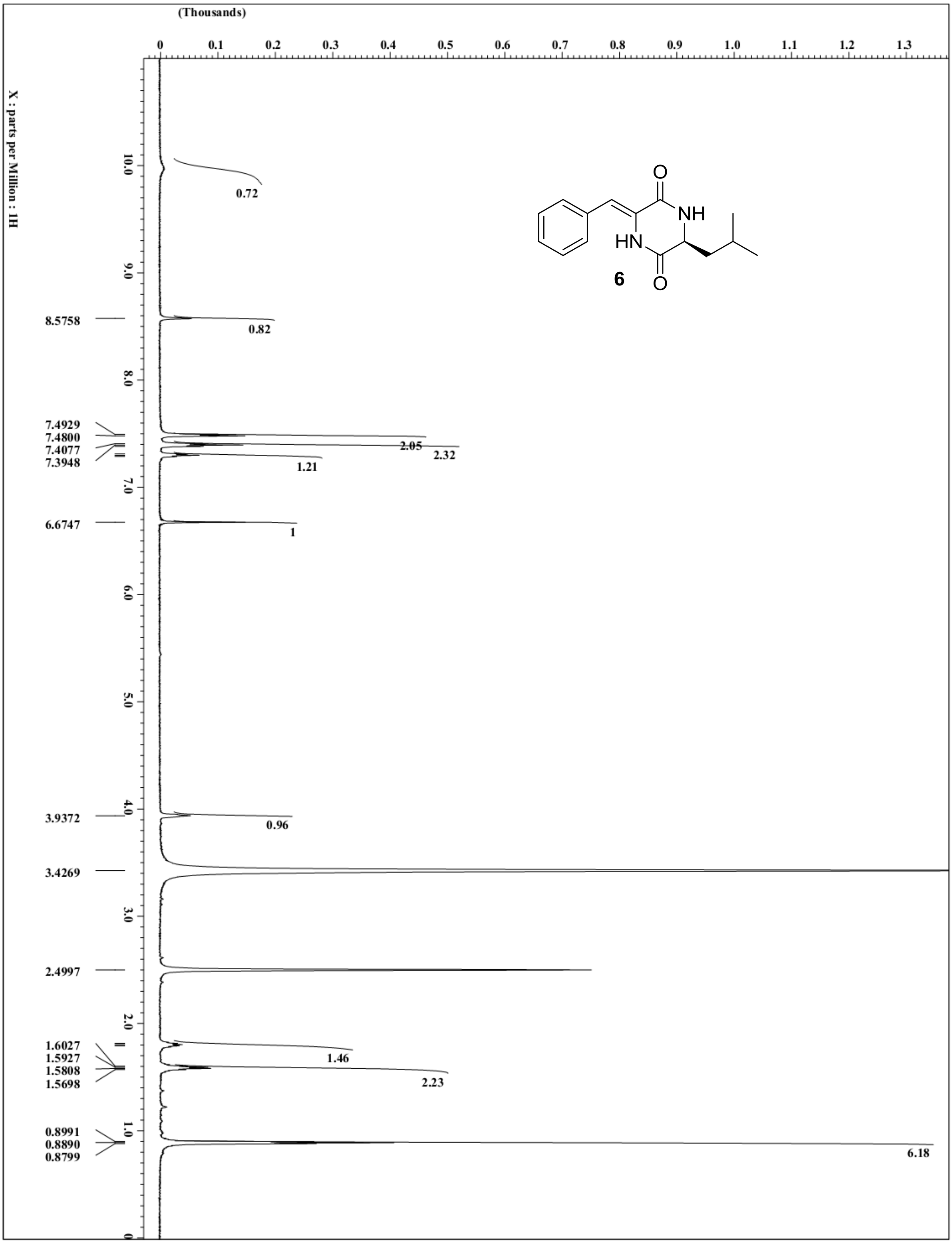

Figure S29. The ^{13}C NMR (150 MHz, DMSO-d_6) spectrum of compound **6** in DMSO-d_6.

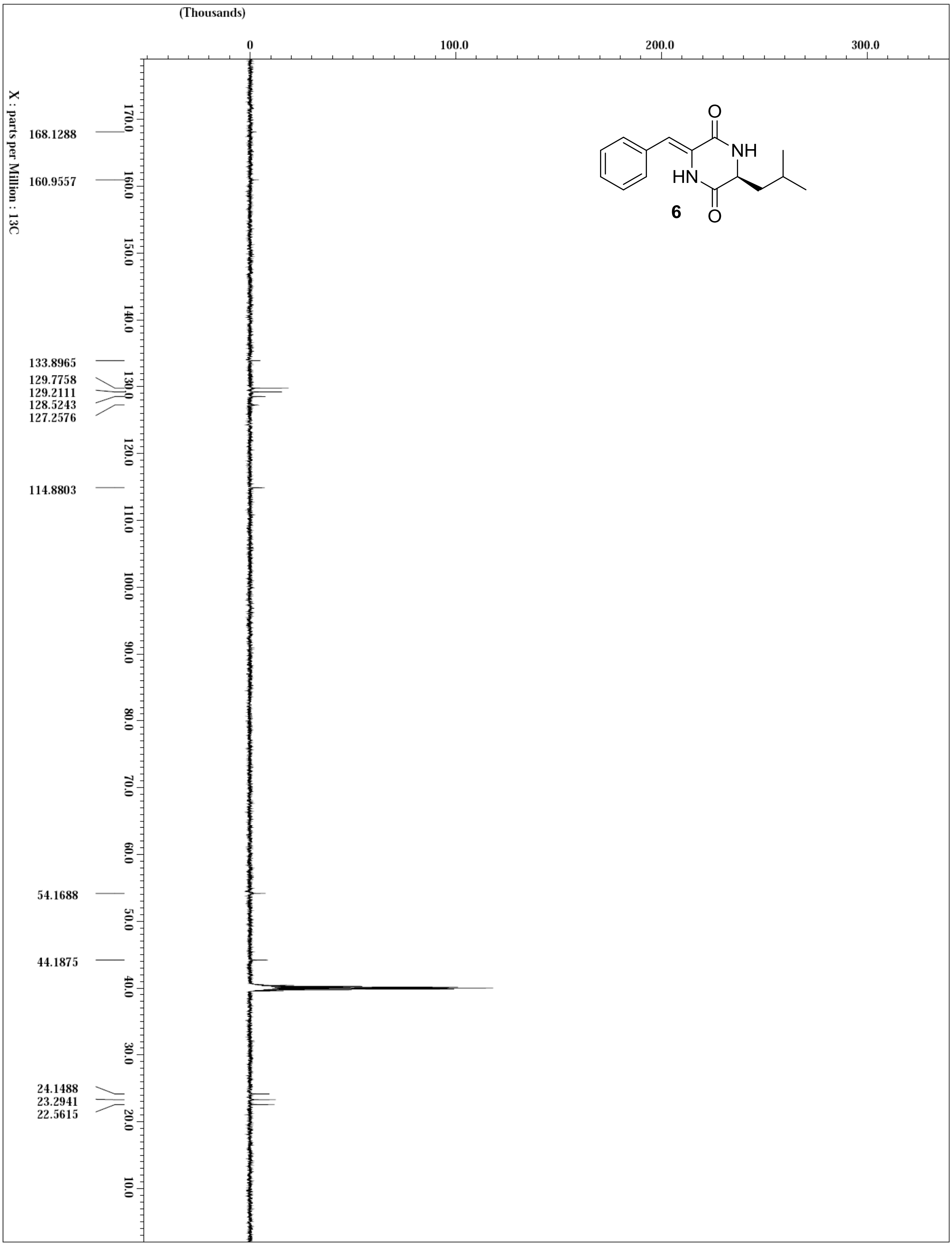

Figure S30. The DEPT (150 MHz, DMSO-d_6) spectrum of compound **6**.

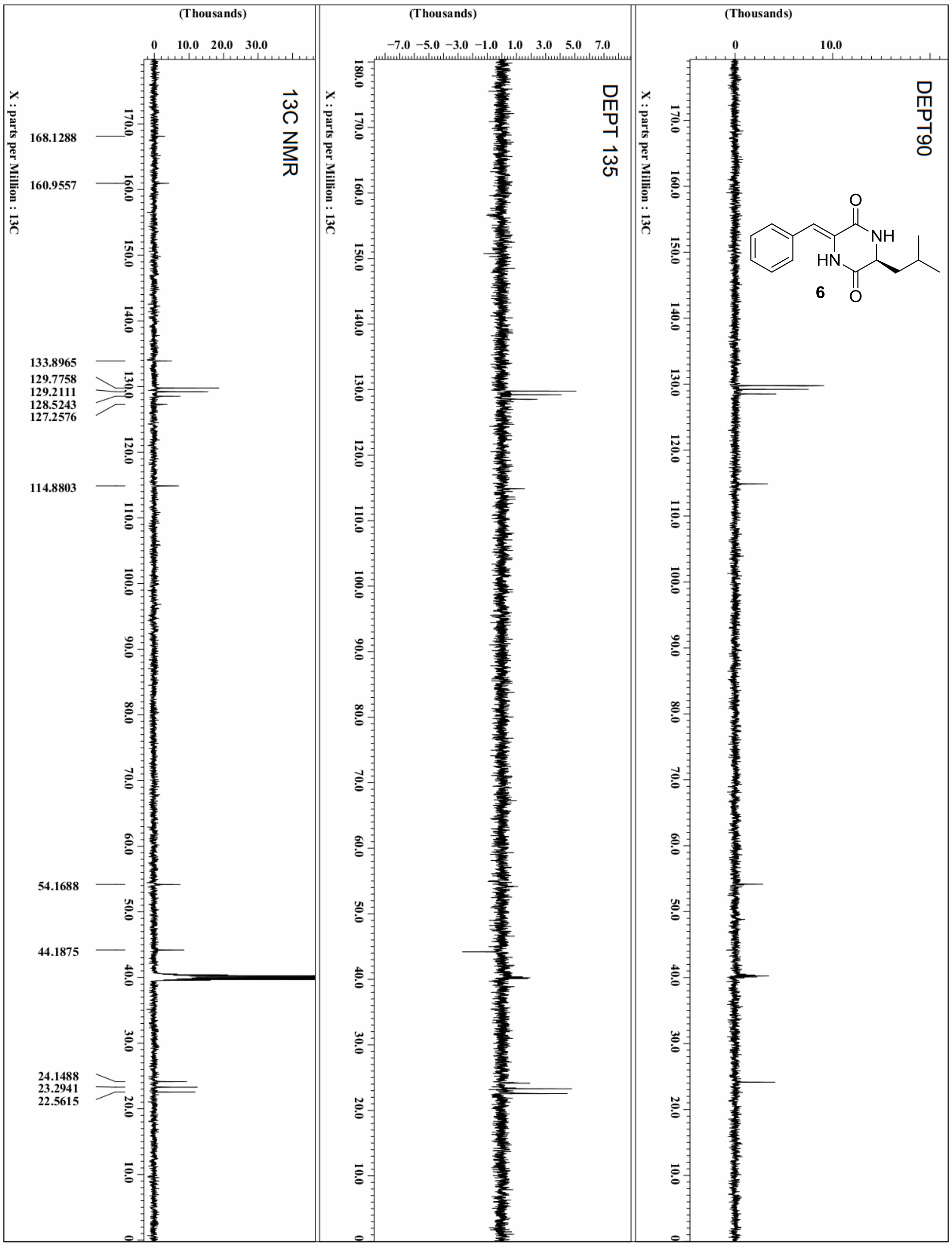

Figure S31. The ^{1}H NMR (600 MHz, DMSO-d_6) spectrum of (*S*)-**1** MTPA ester (**1a**).

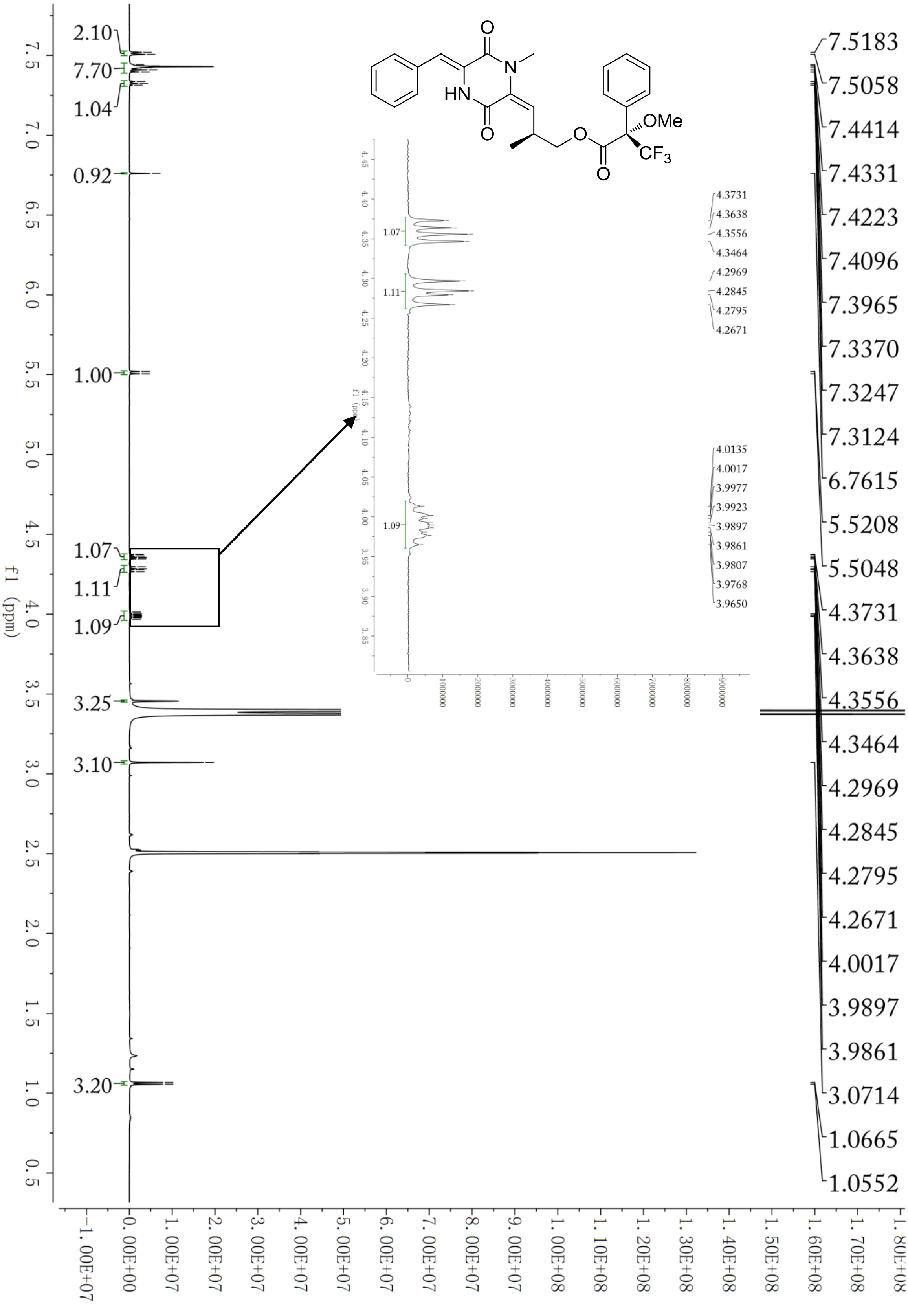

Figure S32. The ^{1}H NMR (600 MHz, DMSO-d_6) spectrum of (*R*)-**1** MTPA ester (**1b**) in DMSO-d_6.

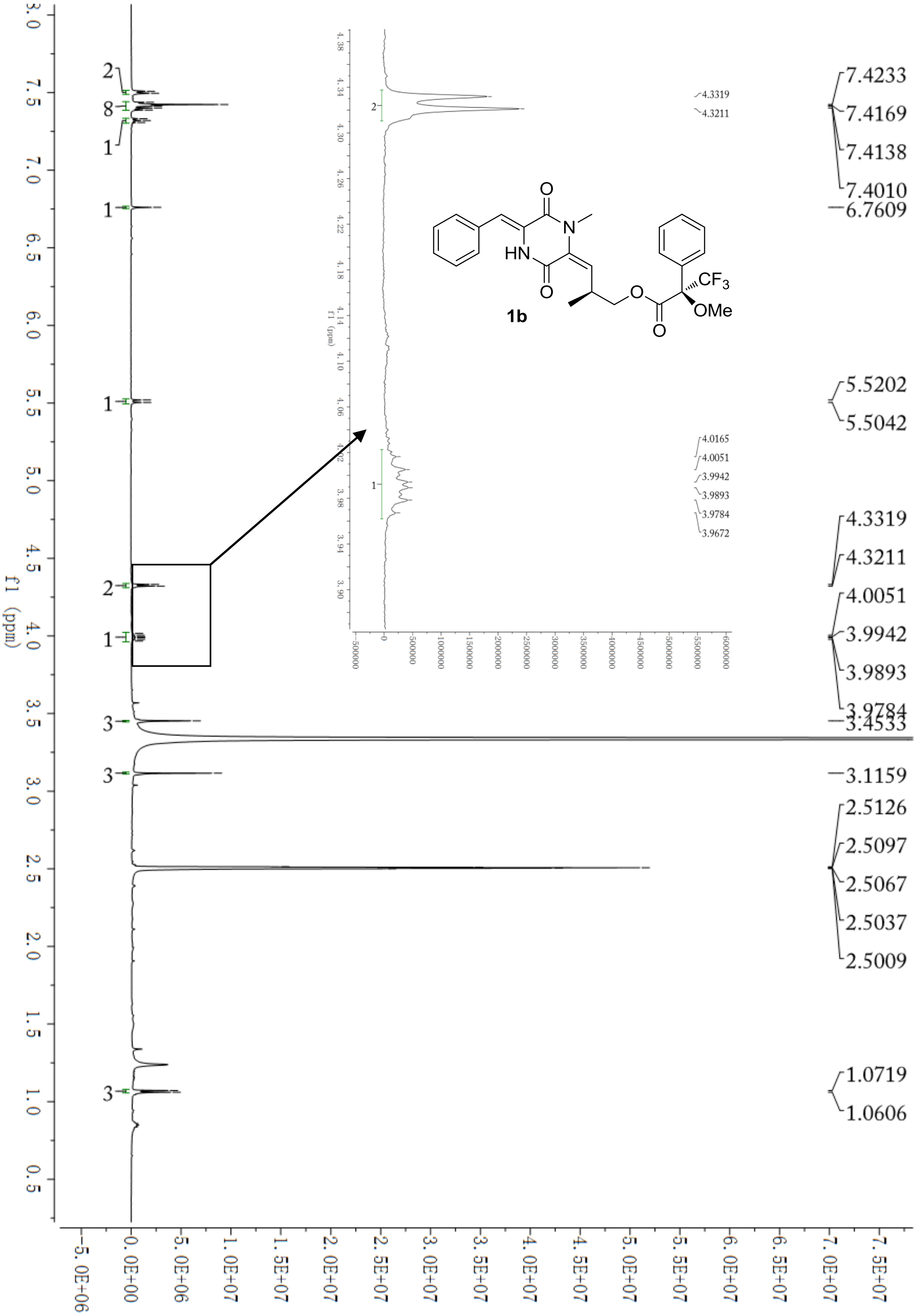

Figure S33. The ^{1}H NMR (600 MHz, DMSO-d_6) spectrum of (*S*)-**2** MTPA ester (**2a**).

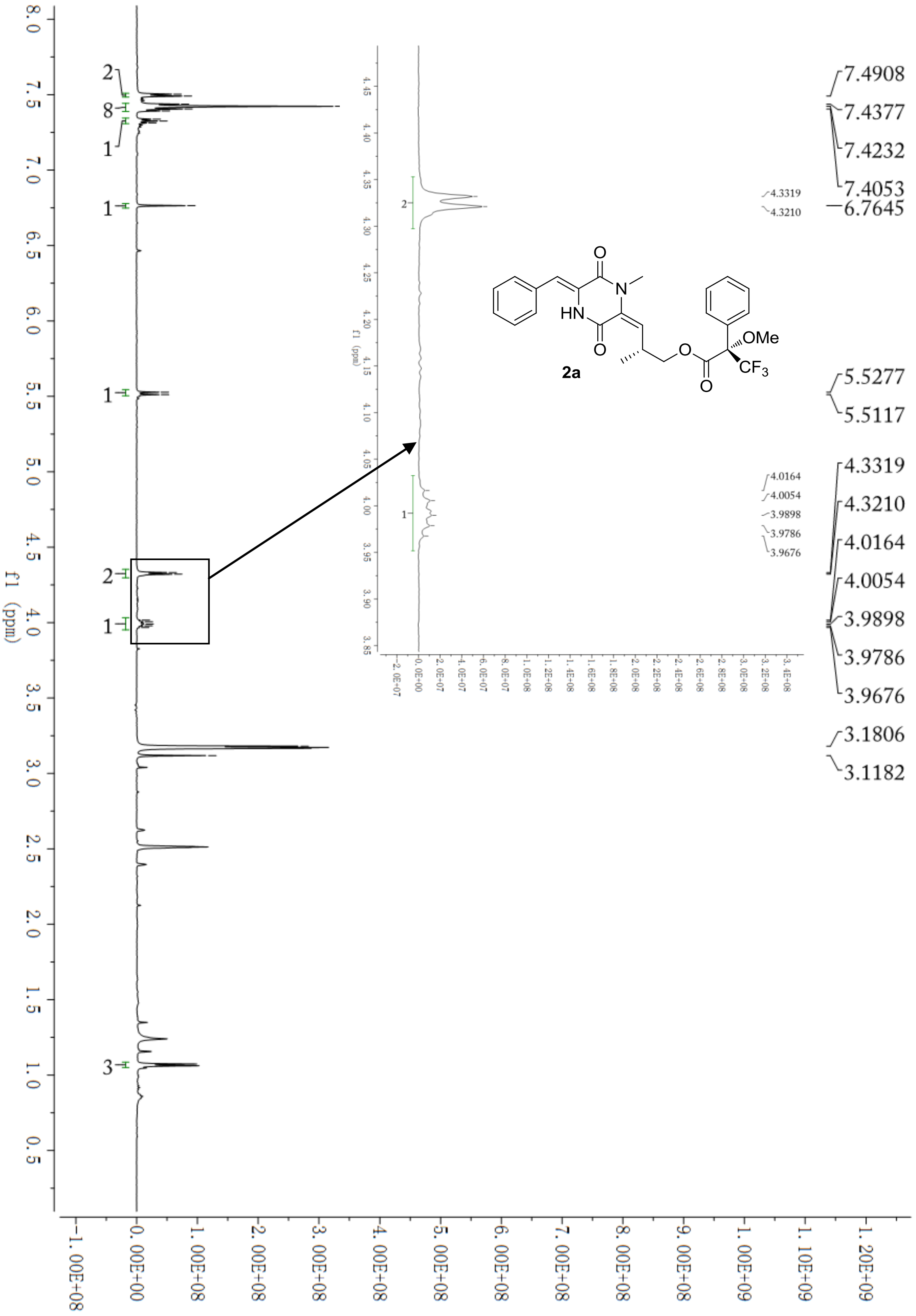

Figure S34. The ^{1}H NMR (600 MHz, DMSO-d_6) spectrum of (*R*)-**2** MTPA ester (**2b**) in DMSO-d_6.

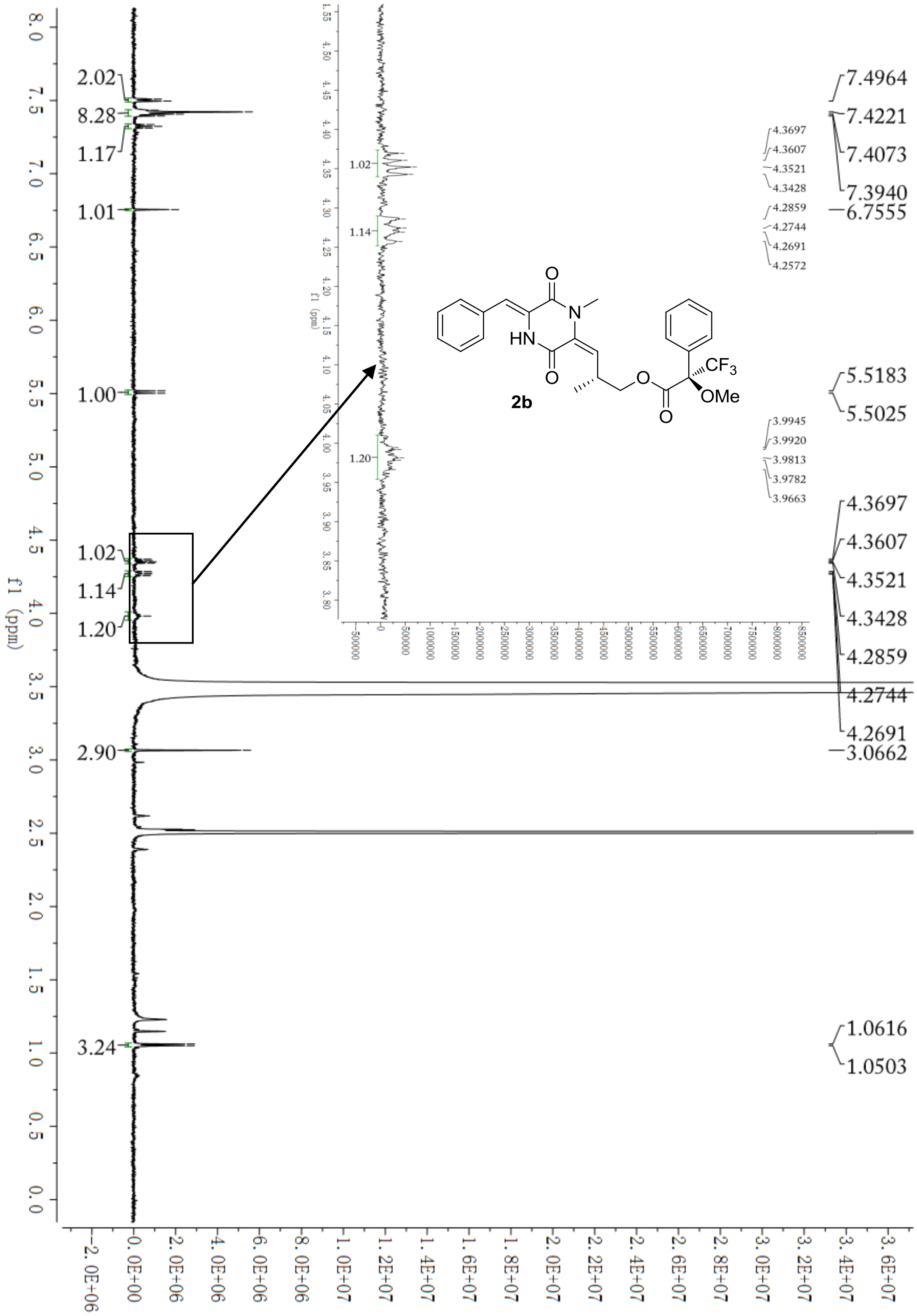

Figure S35. The ^{1}H NMR (600 MHz, DMSO-d_6) spectrum of *p*-bromobenzoate (**1c**).

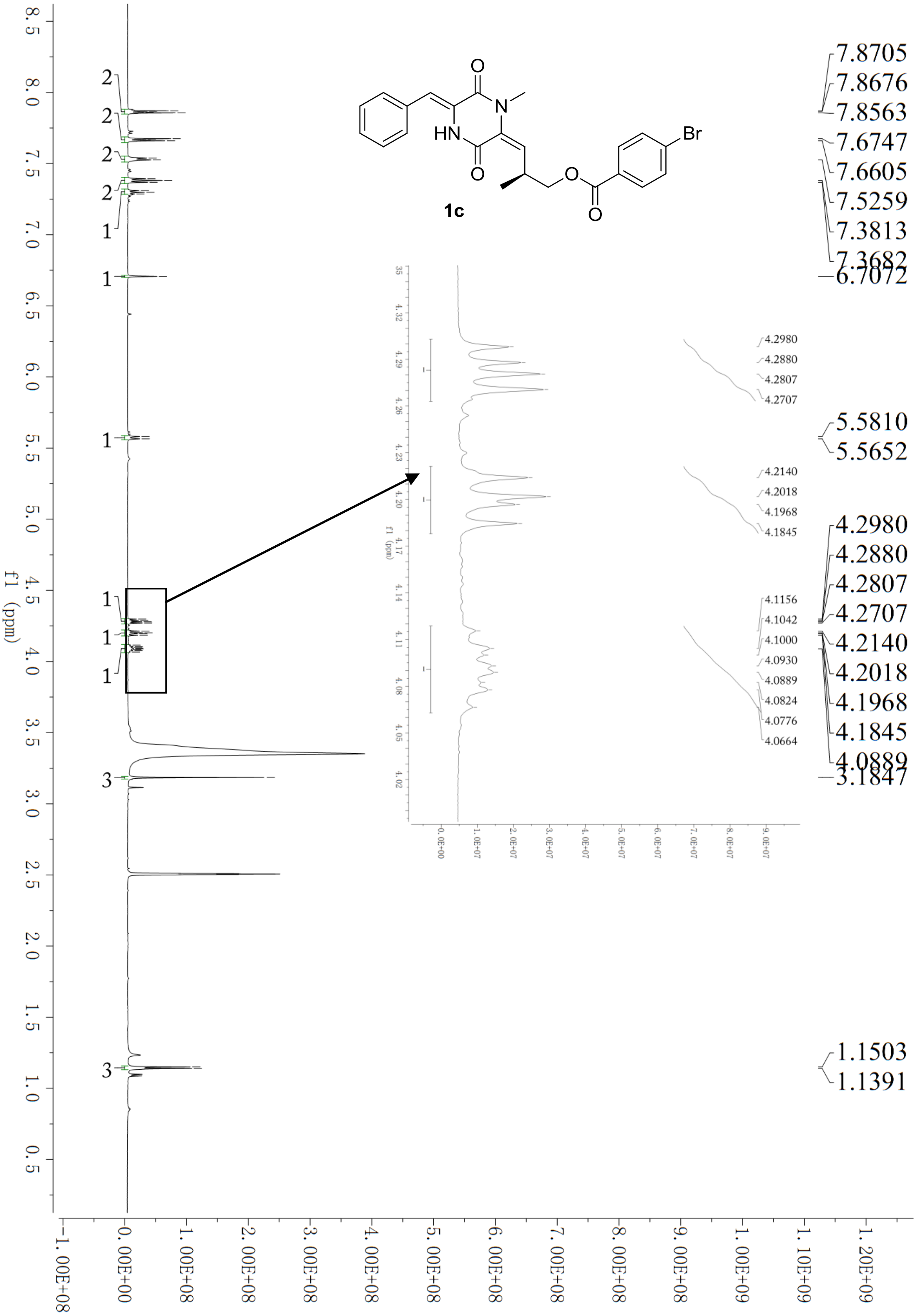

Figure S36. The DEPTQ (150 MHz, DMSO-d_6) spectrum of *p*-bromobenzoate (**1c**).

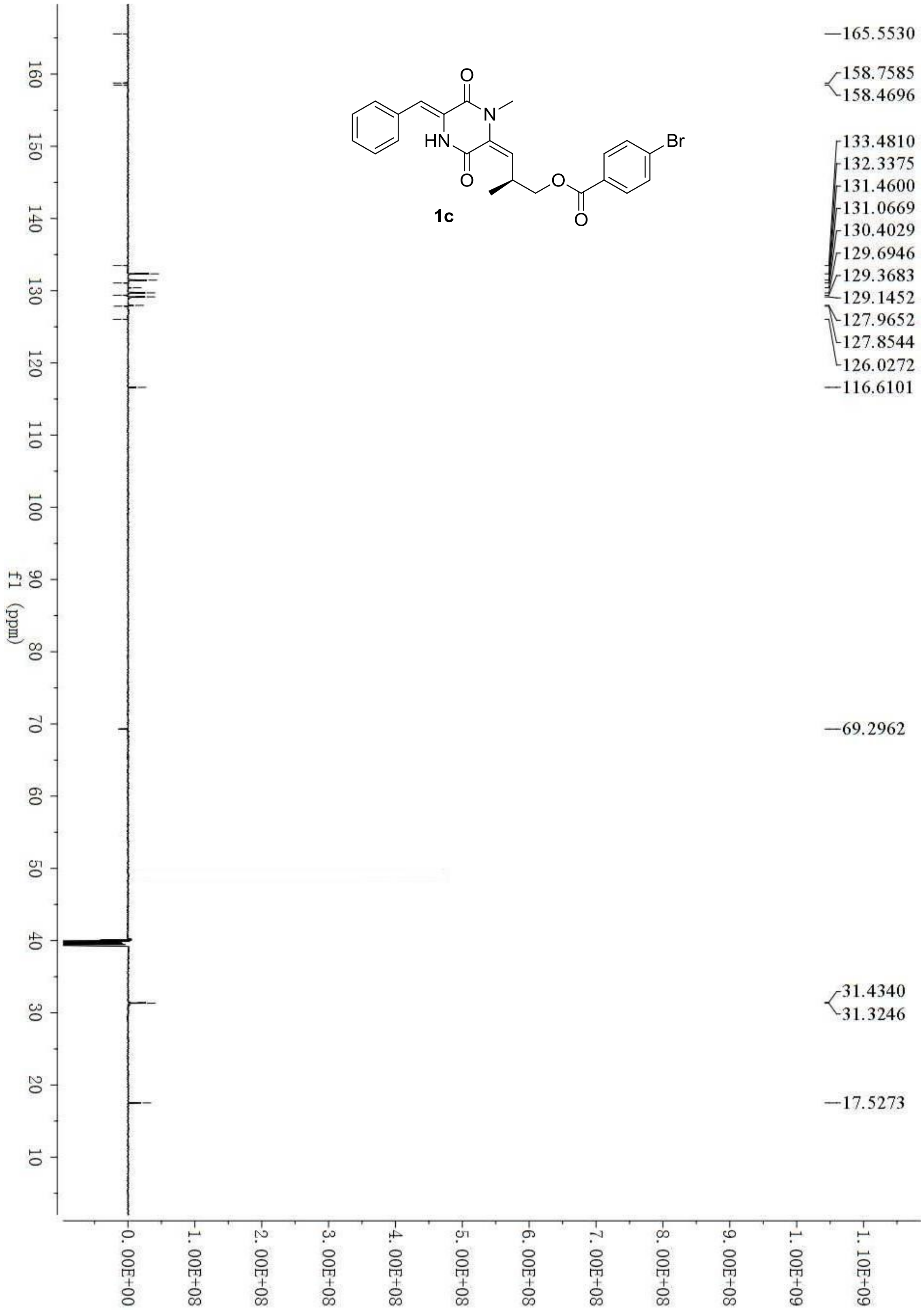

Figure S37. The resolution of the racemic mixture of **1**/**2** by chiral column (CHIRALPAK AY, 4.6 × 150 mm, 0.5 mL/min).

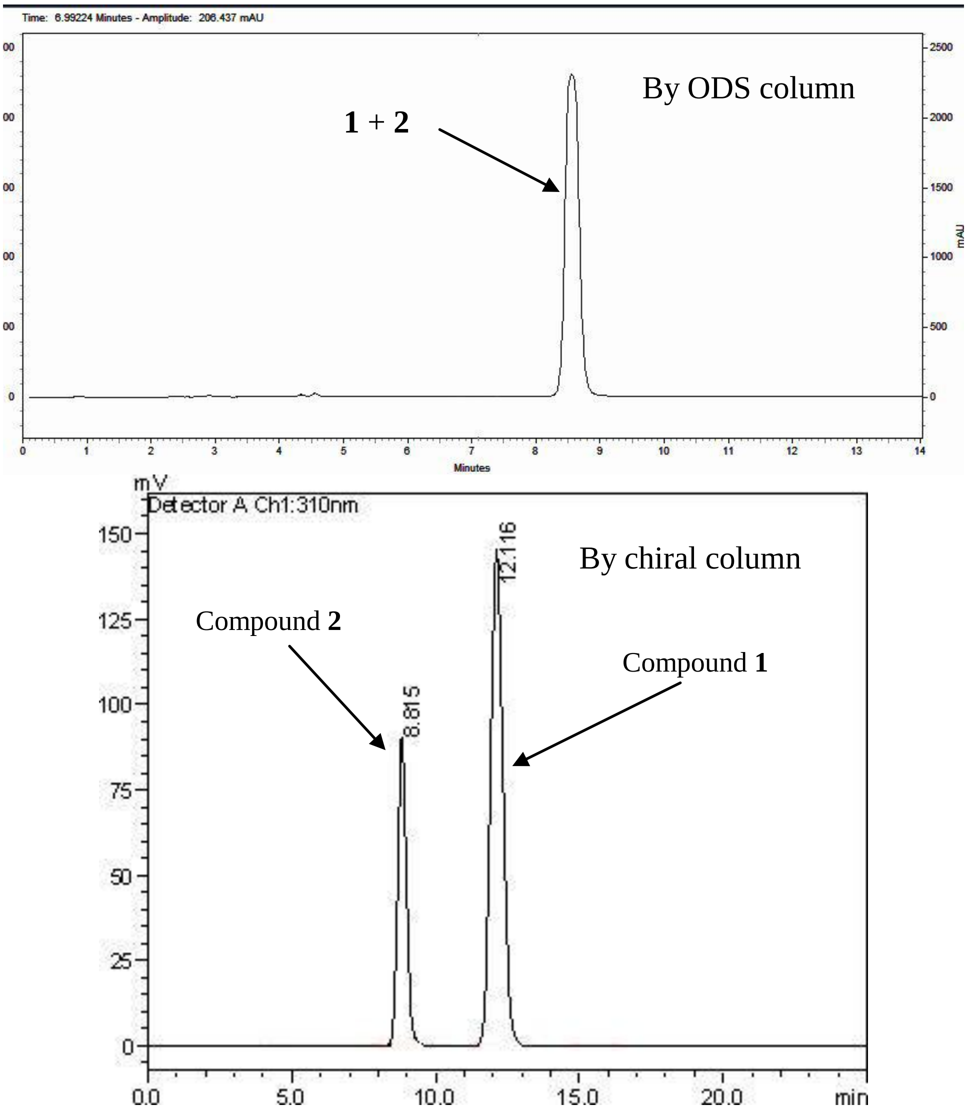

Figure S38. Marfey's method applying for compound **5** (ODS column; Solvents: (A) water + 0.2% TFA, (B) MeCN; linear gradient: 0 min, 75% A + 25% B; 40 min, 40% A + 60% B; 45 min, 100% B; temperature, 30 ℃; flow rate, 1 mL/min; UV detection at λ_{max} 340 nm; FDAA, 16.0 min).

Co-injection of FDAA derivatives of standard D-Ile with standard D-*allo*-Ile by ODS column

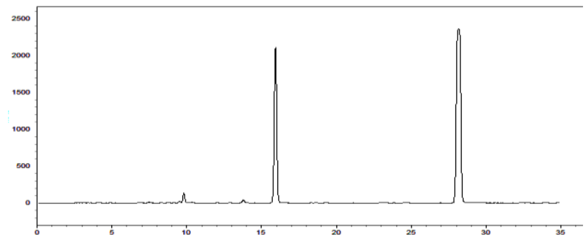

Co-injection of FDAA derivatives of standard L-Ile with standard L-*allo*-Ile by ODS column

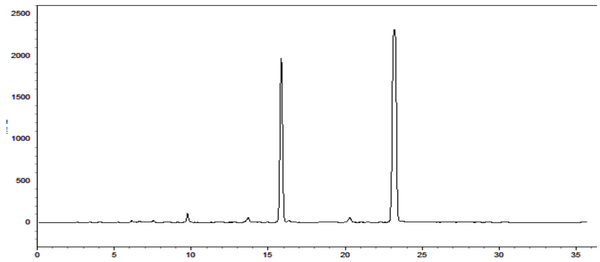

FDAA derivatives of the acid hydrolysate of **5** by ODS column

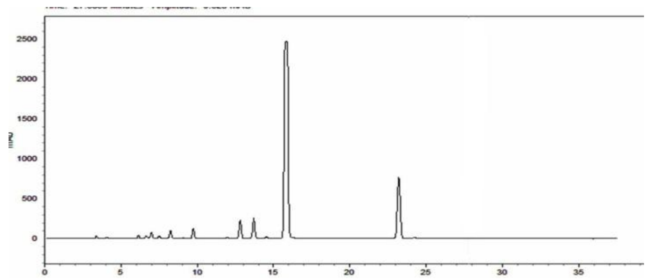

Co-injection of FDAA derivatives of the acid hydrolysates of **5** with standard L-Ile and L-*allo*-Ile by ODS column

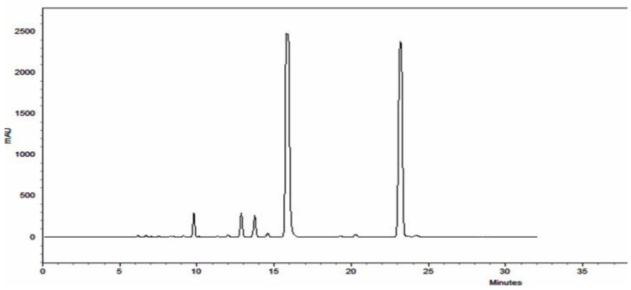

Figure S39. C_3 Marfey's method applying for compound **5** (C_3 column; The column was developed with a linear gradient of 15%–60% MeOH/water (+isocratic 5% of a 1% formic acid solutionin MeCN, over 55 min; temperature, 50 °C; flow rate, 1 mL/min; UV detection at λ_{max} 340 nm; FDAA, 17.95 min).

Co-injection of FDAA derivatives of standard L-Ile with standard L-*allo*-Ile by C_3 column

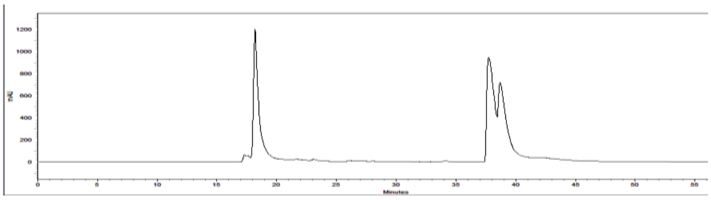

FDAA derivatives of standard L-*allo*-Ile by C_3 column

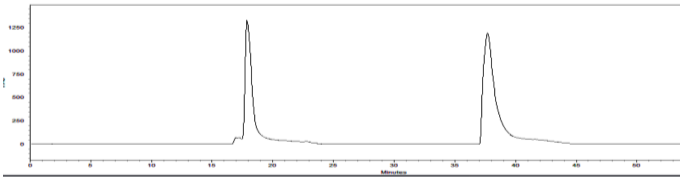

FDAA derivatives of standard L-Ile by C_3 column

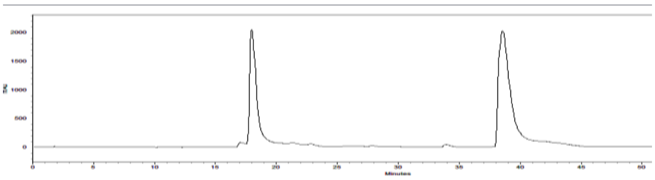

FDAA derivatives of the acid hydrolysate of **5** by C_3 column

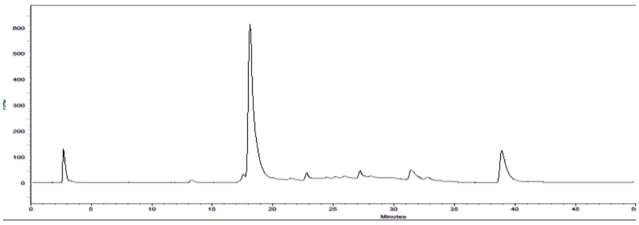

Co-injection of FDAA derivatives of the acid hydrolysates of **5** with standard L-Ile by C_3 column

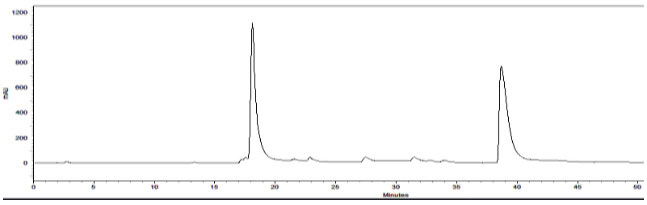

Figure S40. Dose-response histograms of antivirus activity for compound **3** and ribavirin ($p > 0.05$).

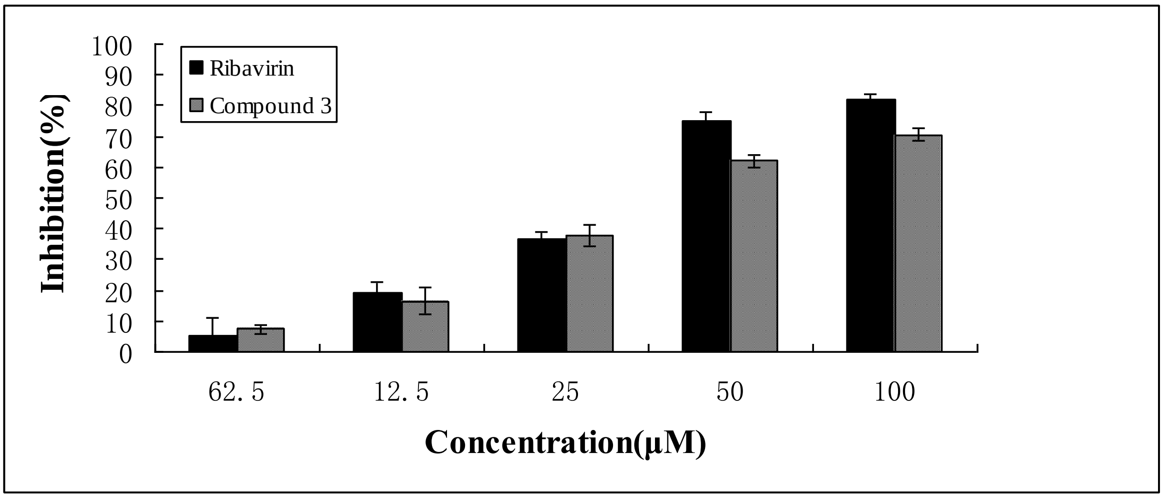

Table S1. Cytotoxic, anti-virus, anti-microbial and anti-inflammatory activities of **1**–**10**.

**Compounds**	**MIC (μg/mL)**						**IC$_{50}$ (μM)**			**IC$_{50}$ (μM)**	**IC$_{50}$ (μM)**
	E. coli	***E. aerogenes***	***P. aeruginosa***	***B. subtilis***	***S. aureus***	***C. albicans***	**HL-60**	**K562**	**A549**	**H1N1**	**RAW 264.7**
**1**	>100	>100	>100	>100	>100	>100	>100	>100	>100	75.5 ± 2.2	>10
**2**	>100	>100	>100	>100	>100	>100	>100	>100	>100	>50	>10
**3**	>100	>100	>100	>100	>100	>100	>100	>100	>100	41.1 ± 4.5	>10
**4**	>100	>100	>100	>100	>100	>100	>100	>100	>100	62.6 ± 3.9	>10
**5**	>100	>100	>100	>100	>100	>100	>100	>100	>100	106.5 ± 4.2	>10
**6**	>100	>100	>100	>100	>100	>100	ND [a]	ND [a]	ND [a]	28.9 ± 2.2	ND [a]
**7**	>100	>100	>100	>100	>100	>100	ND [a]	ND [a]	ND [a]	6.8 ± 1.5	ND [a]
**8**	>100	>100	>100	>100	>100	>100	ND [a]	ND [a]	ND [a]	94.5 ± 3.0	ND [a]
**9**	>100	>100	>100	>100	>100	>100	ND [a]	ND [a]	ND [a]	113.8 ± 4.9	ND [a]
**10**	>100	>100	>100	>100	>100	>100	ND [a]	ND [a]	ND [a]	156.6 ± 4.0	ND [a]
Ciprofloxacin	0.05	0.19	0.1	0.39	3.12	ND [a]	ND [a]	ND [a]	ND [a]	ND [a]	ND [a]
Ketoconazole	ND [a]	ND [a]	ND [a]	ND [a]	ND [a]	0.024	ND [a]	ND [a]	ND [a]	ND [a]	ND [a]
PDTC	ND [a]	ND [a]	ND [a]	ND [a]	ND [a]	ND [a]	ND [a]	ND [a]	ND [a]	ND [a]	<0.1
Adriamycin	ND [a]	ND [a]	ND [a]	ND [a]	ND [a]	ND [a]	0.652	0.645	0.080	ND [a]	ND [a]
Ribavirin	ND [a]	ND [a]	ND [a]	ND [a]	ND [a]	ND [a]	ND [a]	ND [a]	ND [a]	38.8 ± 1.5	ND [a]

[a] Not detected.

Table S2. The HMBC correlations (H → C) for compounds **1**–**5**.

**Proton (position)**	**Carbon (position)**			
	**1 and 2**	**3**	**4**	**5**
1	2, 6	3, 5		5
2				
3				
4		2, 6		
5				
6				
7	5, 6, 9, 10	5, 6, 9, 10	5, 8	
8	7, 9, 10			
9	7, 10	7, 8	7, 8	7, 8
10	7, 8, 9	7, 8	7, 8	6, 7, 8
11	2, 13	2, 13/17	2, 12, 13	2, 13
12				
13/17	11, 15	11, 15	12, 15	11, 15
14/16	12, 15	12, 15		12, 15
15	13/17		12, 13	13
